# Supplementary material for: Genome-wide characterization of 54 urinary metabolites reveals molecular impact of kidney function
Source: Nat Commun. 2025 Jan 2;16:325. doi: 10.1038/s41467-024-55182-1 (PMC11696681; doi:10.1038/s41467-024-55182-1)

Supplementary Information

Genome-wide characterization of 54 urinary metabolites reveals molecular impact of kidney function

Valo Erkka, Richmond Anne, Mutter Stefan, Dahlström Emma H, Campbell Archie, Porteous David J, Wilson James F, FinnDiane Study Group, Groop Per-Henrik, Hayward Caroline, Sandholm Niina

Supplementary Table 1. Characteristics of the study cohorts.
Supplementary Table 2. Previously reported traits for the lead variants from the GWAS catalog.
Supplementary Table 3. MAGMA tissue enrichment analysis, tissues with p<0.01.
Supplementary Table 4. Mendelian randomization analysis outcomes and exposures.
Supplementary Table 5. FinnDiane physicians and nurses participating in the collection of the FinnDiane study subjects.

Supplementary Figure 1. Heritability estimates for the urinary metabolites.
Supplementary Figure 2. LocusZoom plots of the COJO lead signals.
Supplementary Figure 3. Study specific effect estimates for urinary metabolite associations with between study heterogeneity.
Supplementary Figure 4. METAL effect estimates at the COJO lead variants for the associated metabolite and creatinine.
Supplementary Figure 5. *AGTX2* expression in the kidneys.
Supplementary Figure 6. Tyrosine GWAS signal vs. *SLC6A18* and *SLC6A19* kidney eQTL signal.
Supplementary Figure 7. Regional association with 3-aminoisobutyrate and blood and kidney eQTL target genes
Supplementary Figure 8. Two-sample Mendelian Randomization results scatter plots for association of eGFR with urinary metabolites
Supplementary Figure 9. Effect of covariate adjustment on the metabolite associations in FinnDiane.
Supplementary Figure 10. Participant selection criteria in FinnDiane, VIKING and Generation Scotland studies.

Supplementary Table 1

Characteristics of the study cohorts.

	**FinnDiane (N=3,244)**	**GS (N=2,743)**	**VIKING (N=2,024)**
**Age (years)**			
Mean (SD)	37.7 (12.2)	55.8 (10.5)	49.8 (15.2)
Median [Min, Max]	36.7 [13.8, 78.4]	57 [18, 93]	49.7 [18, 92]
**Sex**			
Male	1632 (50.3%)	1373 (50.05%)	811 (40.1%)
Female	1612 (49.7%)	1370 (49.95%)	1213 (59.9%)
**BMI (kg/m²)**			
Mean (SD)	25.1 (3.53)	27.0 (5.0)	27.4 (4.88)
Median [Min, Max]	24.7 [15.0, 46.5]	26.3 [15.9, 71.3]	26.7 [17.0,50.9]
Missing	63 (1.9%)	7 (0.25%)	4 (0.2%)
**Diabetes**			
Type 1 diabetes	3244 (100%)	18 (0.065%)	0 (0%)
Type 2 diabetes	0 (0%)	170 (6.2%)	36 (1.8%)
No	0 (0%)	2500 (91%)	1988 (98.2%)
**eGFR (mL/min/1.73m²)**			
Mean (SD)	98.2 (25.1)	87.7 (16.9)	93.1 (16.6)
Median [Min, Max]	102 [10.2, 190]	87 [17, 167]	94.3 [30.6, 132]
Missing	5 (0.2%)	67 (2.4%)	16 (0.8%)
**CKD category**			
CKD1	2267 (69.9%)	1151 (42%)	1231 (60.8%)
CKD2	712 (21.9%)	1432 (52%)	722 (35.7%)
CKD3	185 (5.7%)	90 (3.3%)	55 (2.7%)
CKD4	59 (1.8%)	3 (0.01%)	0 (0%)
CKD5	16 (0.5%)	0 (0%)	0 (0%)
Missing	5 (0.2%)	67 (2.4%)	16 (0.8%)
**Albuminuria status**			
Normal AER	2233 (68.8%)	NA	NA
Moderate albuminuria	441 (13.6%)	NA	NA
Severe albuminuria	427 (13.2%)	NA	NA
Missing	143 (4.4%)	2743 (100%)	2024 (100%)

Supplementary Table 2

Previously reported traits for the lead variants from the GWAS catalog. Previously associated traits in the GWAS catalog (window size=-/+500kb, $r^2 > 0.8$, and $p < 5e-08$) for the lead variants (n=30) associated with urinary metabolites. The 'Previously reported urinary/blood/other traits' columns lists previously associated variants with the reported trait (p-value annotation). The previously reported traits are classified as urinary, blood or other by searching key words.

Chr:Pos	Rsid	Metabolite	Previously associated urinary metabolite traits	Previously associated blood metabolite traits	Previously associated other traits
1:11940483	rs4846068	Formate			rs11588551<br>Blood protein levels *(KIAA2013, 6538_90_3)*<br>Serum levels of protein KIAA2013<br>rs198384<br>Sex hormone-binding globulin levels<br>Sex hormone-binding globulin levels adjusted for BMI
1:48690229	rs10788884	Xylose	rs10788884<br>Urine mannose levels in chronic kidney disease	rs926979<br>Serum uric acid levels	rs926979<br>Urate levels
1:151904146	rs2999545	3-Hydroxyisovalerate	rs7541453<br>Urine X-24363 levels in chronic kidney disease	rs1060870<br>Serum metabolite levels *(3-hydroxylaurate)*<br>Serum metabolite levels *(3-hydroxyoctanoate)*<br>rs10788817<br>Serum metabolite levels *(3-hydroxydecanoate)*<br>rs2279501<br>Serum metabolite levels *(3-hydroxysebacate)*<br>rs7541453<br>Serum metabolite levels *(indoleacetylglutamine)*	rs1038747<br>Plasma X-21607 levels in chronic kidney disease<br>rs10788817<br>3-hydroxylaurate levels<br>Metabolite peak levels (QI2718)<br>Tetradecadienoate (14:2) levels<br>rs10888445<br>Beta-hydroxyisovalerate levels<br>rs11204914<br>Carnitine C9 levels<br>Metabolite peak levels (QI6204)<br>Metabolite peak levels (QI6252)<br>rs2130525<br>Glycine conjugate of C10H14O2 (1) levels<br>rs2338201<br>Dodecadienoate (12:2) (X-21343) levels<br>Dodecadienoate (12:2) levels<br>Tetradecadienoate (14:2) (X-12442) levels<br>rs2999541<br>Cis-3,4-methyleneheptanoylcarnitine levels<br>rs2999545<br>Pimeloylcarnitine/3-methyladipoylcarnitine (C7-DC) levels

Chr:Pos	Rsid	Metabolite	Previously associated urinary metabolite traits	Previously associated blood metabolite traits	Previously associated other traits
					rs6685187<br>X-12442–5,8-tetradecadienoate levels<br>rs6698740<br>Beta-hydroxyisovalerate levels<br>rs7523082<br>Metabolite peak levels (QI9845)<br>rs7542137<br>3-hydroxysebacate levels<br>Beta-hydroxyisovalerate levels<br>Plasma cis-4-decenoate (10:1n6)* levels in chronic kidney disease
2:135598913	rs17322446	1-Methylnicotinamide	rs10496732<br>Urine quinolinate levels in chronic kidney disease		rs17322446<br>1-methylnicotinamide levels<br>N1-methyl-2-pyridone-5-carboxamide levels<br>rs17698630<br>Walking pace<br>rs1942052<br>Quinolinate levels<br>rs41449851<br>FEV1<br>rs76328456<br>Quinolinate levels
2:211540507	rs1047891	Glycine	rs1047891<br>Urinary metabolite levels in chronic kidney disease *(2-methylbutyrylglycine)*<br>Urinary metabolite levels in chronic kidney disease *(3-methylcrotonylglycine)*<br>Urinary metabolite levels in chronic kidney disease *(3-methylglutaconate)*<br>Urinary metabolite levels in chronic kidney disease *(3-methylglutarate/2-methylglutarate)*<br>Urinary metabolite levels in chronic kidney disease *(3-methylglutarylcarnitine (2))*<br>Urinary metabolite levels in chronic kidney disease *(X - 16567)*<br>Urinary metabolite levels in chronic kidney disease *(X - 24402)*<br>Urinary metabolite levels in chronic	rs1047891<br>Blood urea nitrogen levels<br>Serum metabolite concentrations in chronic kidney disease *(Glycine)*<br>Serum metabolite levels *(3-methylglutaconate)*<br>Serum metabolite levels *(3-methylglutarylcarnitine (2))*<br>Serum metabolite levels *(3b-hydroxy-5-cholenoic acid)*<br>Serum metabolite levels *(N-acetylglycine)*<br>Serum metabolite levels *(N-palmitoylglycine)*<br>Serum metabolite levels *(betaine)*<br>Serum metabolite levels *(cinnamoylglycine)*<br>Serum metabolite levels *(creatine)*<br>Serum metabolite levels *(gamma-glutamylglycine)*<br>Serum metabolite levels *(glycine)*	rs1047891<br>1-ribosyl-imidazoleacetate (X-11334) levels<br>2-butenoylglycine levels<br>2R,3R-dihydroxybutyrate levels<br>3-hydroxybutyroylglycine levels<br>3-methylglutaconate levels<br>3-methylglutarylcarnitine (2) levels<br>5-methylthioadenosine (mta) levels<br>Alanine aminotransferase levels<br>Alanine levels<br>Alanine transaminase levels<br>Apolipoprotein A1 levels<br>Appendicular lean mass<br>Betaine levels<br>Body surface area<br>Butyrylglycine levels<br>Ceramide (d17:1/24:1) levels<br>Cholesterol levels in medium HDL<br>Cholesterol to total lipids ratio in IDL<br>Cholesterol to total lipids ratio in small LDL<br>Cholesteryl ester levels in HDL

Chr:Pos	Rsid	Metabolite	Previously associated urinary metabolite traits	Previously associated blood metabolite traits	Previously associated other traits
			kidney disease *(hexanoylglycine)*<br>Urinary metabolite levels in chronic kidney disease *(isobutyrylglycine)*<br>Urinary metabolite levels in chronic kidney disease *(isocaproylglycine)*<br>Urinary metabolite levels in chronic kidney disease *(isovalerylglycine)*<br>Urinary metabolite levels in chronic kidney disease *(tigloylglycine)*<br>Urinary metabolite modules (eigenmetabolites) in chronic kidney disease *(2-methylbutyrylglycine, 3-methylcrotonylglycine, indoleacetylglutamine, indoleacetylglycine, isobutyrylglycine, isovalerylglutamine, isovalerylglycine, tigloylglycine, X-17688)*<br>Urinary metabolite modules (eigenmetabolites) in chronic kidney disease *(3-hydroxy-3-methylglutarate, 3-methylglutaconate, 3-methylglutarate, 2-methylglutarate, 3-methylglutarylcarnitine)*<br>Urinary metabolite modules (eigenmetabolites) in chronic kidney disease *(hexanoylglycine, isocaproylglycine, X-16567, X-24402)*<br>Urine 2-butenoylglycine levels in chronic kidney disease<br>Urine 2-methylbutyrylglycine levels in chronic kidney disease<br>Urine 3-hydroxyoctanoyl glycine levels in chronic kidney disease<br>Urine 3-methylcrotonylglycine levels in chronic kidney disease<br>Urine 3-methylglutaconate levels in chronic kidney disease<br>Urine 3-methylglutarylcarnitine (2) levels in chronic kidney disease<br>Urine 4-methylhexanoylglycine levels in chronic kidney disease	Serum metabolite levels *(homoarginine)*<br>Serum metabolite levels *(isobutyrylglycine)*<br>Serum metabolite levels *(isovalerylglycine)*<br>Serum metabolite levels *(propionylglycine)*<br>Serum metabolite levels *(serine)*<br>Serum uric acid levels<br>rs715<br>Serum metabolite levels *(Creatine)*<br>Serum metabolite levels *(Glycine)*	Cholesteryl ester levels in medium HDL<br>Chronic elevation of alanine aminotransferase (cALT) levels<br>Chronic kidney disease<br>Cinnamoylglycine levels<br>Cis-3,4-methyleneheptanoylglycine levels<br>Citrate levels<br>Citrate levels (UKB data field 23473)<br>Concentration of HDL particles<br>Concentration of medium HDL particles<br>Creatine levels<br>Creatinine levels<br>Creatinine levels (UKB data field 23478)<br>Diastolic blood pressure<br>Eosinophil counts<br>Estimated glomerular filtration rate<br>Estimated glomerular filtration rate (creatinine)<br>Estimated glomerular filtration rate (creatinine) *(conditioned on rs72944180)*<br>Estimated glomerular filtration rate (cystatin c)<br>Estimated glomerular filtration rate *(EA)*<br>Estimated glomerular filtration rate in diabetes<br>Estimated glomerular filtration rate in non-diabetics<br>Fat-free mass<br>Fat-free mass *(female)*<br>Free androgen index<br>Free cholesterol levels in medium HDL<br>Gamma-glutamylglycine levels<br>Glomerular filtration rate (creatinine)<br>Glutarylcarnitine (c5-dc) levels<br>Glycine levels<br>Glycine levels (UKB data field 23462)<br>Glycine levels in chronic kidney disease<br>Glycosyl-N-(2-hydroxynervonoyl)-sphingosine (d18:1/24:1(2OH)) levels<br>HDL cholesterol<br>HDL cholesterol levels<br>HDL cholesterol levels in current drinkers<br>HDL cholesterol levels x alcohol consumption (drinkers vs non-drinkers) interaction (2df)<br>HDL cholesterol levels x alcohol consumption (drinkers vs non-drinkers) interaction (2df) *(EA)*<br>HDL cholesterol levels x alcohol consumption (regular vs non-regular drinkers) interaction (2df)<br>HDL cholesterol levels x alcohol consumption (regular vs non-regular drinkers) interaction (2df) *(EA)*

Chr:Pos	Rsid	Metabolite	Previously associated urinary metabolite traits	Previously associated blood metabolite traits	Previously associated other traits
			Urine 5-hydroxymethyl-2-furoylcarnitine* levels in chronic kidney disease<br>Urine X-17325 levels in chronic kidney disease<br>Urine X-17367 levels in chronic kidney disease<br>Urine X-24465 levels in chronic kidney disease<br>Urine glycine conjugate of C6H10O2 (1)* levels in chronic kidney disease<br>Urine glycine levels in chronic kidney disease<br>Urine hexanoylglycine levels in chronic kidney disease<br>Urine indolepropionylglycine levels in chronic kidney disease<br>Urine isobutyrylglycine levels in chronic kidney disease<br>Urine isocaproylglycine levels in chronic kidney disease<br>Urine isovalerylglycine levels in chronic kidney disease<br>Urine phenylpropionylglycine levels in chronic kidney disease<br>Urine tigloylglycine levels in chronic kidney disease<br>rs715<br>Urinary metabolites *(3.555 ppm/2.547 ppm)*<br>Urinary metabolites *(glycine/threonine)*<br>Urine 2-methylserine levels in chronic kidney disease<br>Urine 3-methylglutarate/2-methylglutarate levels in chronic kidney disease<br>Urine X-24406 levels in chronic kidney disease<br>Urine cis-3,4-methyleneheptanoylglycine levels in chronic kidney disease<br>Urine trans-2-hexenoylglycine levels in chronic kidney disease		Height<br>Hexanoylglycine levels<br>High density lipoprotein cholesterol levels<br>Histidine levels<br>Histidine levels (UKB data field 23463)<br>Homoarginine levels<br>Isobutyrylglycine levels<br>Isovalerylglycine levels<br>Lipoprotein (a) levels<br>Liver enzyme levels (alkaline phosphatase)<br>Lymphocyte count<br>Macular telangiectasia type 2<br>Mean corpuscular hemoglobin<br>Mean corpuscular volume<br>Mean platelet volume<br>Metabolite levels *(Glycine)*<br>Metabolite peak levels (QI6688)<br>Metabolite peak levels (QI6816) *(African American or Afro-Caribbean)*<br>Metabolite peak levels (QI6823)<br>Metabolite peak levels (QI6840)<br>Metabolite peak levels (QI9202)<br>Metabolite peak levels (QI9368)<br>Metabolite peak levels (QI9376)<br>Methylglutarylcarnitine levels<br>N-acetylaspartic acid levels *(African American or Afro-Caribbean)*<br>N-acetylglycine levels<br>N-palmitoylglycine levels<br>NMMA levels<br>Neutrophil count<br>Phospholipids to total lipids ratio in IDL<br>Phospholipids to total lipids ratio in very small VLDL<br>Plasma 2,3-dihydroxy-5-methylthio-4-pentenoate (DMTPA)* levels in chronic kidney disease<br>Plasma 3-methylcrotonylglycine levels in chronic kidney disease<br>Plasma 3-methylglutaconate levels in chronic kidney disease<br>Plasma 3-methylglutarylcarnitine (2) levels in chronic kidney disease<br>Plasma N-acetylglycine levels in chronic kidney disease<br>Plasma N-palmitoylglycine levels in chronic kidney disease<br>Plasma X-25982 levels in chronic kidney disease<br>Plasma betaine levels in chronic kidney disease<br>Plasma choline levels in chronic kidney disease<br>Plasma gamma-glutamylglycine levels in chronic kidney disease<br>Plasma glycine levels in chronic kidney disease<br>Plasma hexanoylglycine levels in chronic kidney disease

Chr:Pos	Rsid	Metabolite	Previously associated urinary metabolite traits	Previously associated blood metabolite traits	Previously associated other traits
			Urine trans-3,4-methyleneheptanoylglycine levels in chronic kidney disease		Plasma homoarginine levels in chronic kidney disease
					Plasma homocysteine levels (post-methionine load test)
					Plasma propionylglycine levels in chronic kidney disease
					Plasma tigloylglycine levels in chronic kidney disease
					Platelet count
					Propionylglycine levels
					Protein quantitative trait loci (liver) *(GLDC)*
					Pyroglutamine levels
					Red blood cell count
					Red cell distribution width
					Red meat related metabolomic signature
					Serine levels
					Serum 25-Hydroxyvitamin D levels
					Serum alkaline phosphatase levels
					Serum creatinine levels
					Serum levels of protein SHMT2
					Serum total protein level
					Sex hormone-binding globulin levels
					Sex hormone-binding globulin levels adjusted for BMI
					Sex hormone-binding globulin levels in postmenopausal women
					Sex hormone-binding globulin levels in premenopausal women
					Systolic blood pressure
					Systolic blood pressure (standard GWA)
					Threonine levels
					Total bilirubin levels
					Total lipid levels in medium HDL
					Trans-2-hexenoylglycine levels
					Urate levels
					Urate levels *(women)*
					Urinary albumin excretion
					Urinary albumin excretion (no hypertensive medication)
					Urinary albumin-to-creatinine ratio
					Urinary albumin-to-creatinine ratio *(EA)*
					Urinary potassium to creatinine ratio
					Urinary sodium to creatinine ratio
					Weight
					White blood cell count
					X-17325 levels
					X-17367 levels
					X-21467 levels
					X-24757 levels
					X-24795 levels
					rs715
					Arginine levels

Chr:Pos	Rsid	Metabolite	Previously associated urinary metabolite traits	Previously associated blood metabolite traits	Previously associated other traits
					Aspartate levels<br>Betaine levels<br>Betaine levels in individuals undergoing cardiac evaluation<br>Body mass index<br>Body mass index (MTAG)<br>Body mass index or knee osteoarthritis (pleiotropy)<br>Calcium levels<br>Cholelithiasis<br>Coronary artery disease or fibrinogen levels (pleiotropy)<br>Creatine levels<br>Creatinine levels<br>Eosinophil counts<br>Eosinophil percentage of granulocytes<br>Eosinophil percentage of white cells<br>Fibrinogen *(EA)*<br>Fibrinogen levels<br>Fibrinogen levels or factor VII levels (pleiotropy)<br>Fibrinogen levels or factor VII levels or factor XI levels or tissue plasminogen activator levels (pleiotropy)<br>Fibrinogen levels or plasminogen activator inhibitor 1 levels (pleiotropy)<br>Fibrinogen levels or tissue plasminogen activator levels (pleiotropy)<br>Gamma-glutamylglycine levels<br>Glomerular filtration rate<br>Glutamine degradant levels<br>Glutaroyl carnitine levels<br>Glycine levels<br>Height<br>Histidine levels<br>Ischemic stroke or fibrinogen levels (pleiotropy)<br>Macular telangiectasia type 2<br>Mean corpuscular hemoglobin<br>Mean corpuscular volume<br>Metabolite levels *(glycine)*<br>N-acetylglycine levels<br>Neutrophil count<br>Phenylalanine levels<br>Plasma 3-hydroxybutyroylglycine** levels in chronic kidney disease<br>Plasma cis-3,4-methyleneheptanoylglycine levels in chronic kidney disease<br>Plasma free amino acid levels (adjusted for twenty other PFAAs) *(Gly)*<br>Platelet distribution width<br>Plateletcrit<br>Propionylglycine levels

Chr:Pos	Rsid	Metabolite	Previously associated urinary metabolite traits	Previously associated blood metabolite traits	Previously associated other traits
					Pyroglutamine levels<br>Red blood cell count<br>Red cell distribution width<br>Serine levels<br>Threonine levels<br>Tyrosine levels<br>Urate levels<br>Venous thromboembolism or fibrinogen levels (pleiotropy)<br>X-08988 levels<br>X-16570 levels<br>X-17325 levels<br>X-25420 levels
2:241793545	rs55649245	4-Deoxyerythronic acid			rs55649245<br>2R,3R-dihydroxybutyrate levels<br>Plasma 2R,3R-dihydroxybutyrate levels in chronic kidney disease<br>X-23639 levels
2:241813788	rs10933641	4-Deoxyerythronic acid	rs6748734<br>Urinary metabolites *(1.086 ppm)*		rs4344931<br>Height<br>rs4675869<br>X-23639 levels<br>rs4675874<br>X-12556 levels<br>rs6739772<br>Hip circumference adjusted for BMI *(EA)*<br>Hip circumference adjusted for BMI *(EA, women)*<br>rs6752657<br>Lung function (FVC)
3:182758040	rs4859267	3-Hydroxyisovalerate			rs11928508<br>Lymphocyte count<br>rs2270968<br>Hydroxyisovaleroyl carnitine levels<br>Parkinson’s disease
4:88213884	rs6811902	Propylene Glycol			rs10026092<br>Protein quantitative trait loci (liver) *(HSD17B13)*<br>rs11735092<br>Height<br>Phospholipids to total lipids ratio in small HDL<br>Platelet count<br>Total testosterone levels<br>rs13133311

Chr:Pos	Rsid	Metabolite	Previously associated urinary metabolite traits	Previously associated blood metabolite traits	Previously associated other traits
					Free androgen index<br>Protein quantitative trait loci (liver) *(HSD17B13)*<br>rs13141053<br>Liver enzyme levels (gamma-glutamyl transferase)<br>Triglyceride levels x long total sleep time interaction (2df test)<br>Triglyceride levels x short total sleep time interaction (2df test)<br>rs13142655<br>Aspartate aminotransferase levels<br>rs13150068<br>Phospholipids to total lipids ratio in large HDL<br>Platelet count<br>Sex hormone-binding globulin levels<br>Sex hormone-binding globulin levels adjusted for BMI<br>Sex hormone-binding globulin levels in postmenopausal women<br>rs6811902<br>Low testosterone levels<br>Sex hormone-binding globulin levels<br>rs6831888<br>Total testosterone levels<br>rs6849526<br>Platelet count<br>rs7694379<br>Alanine transaminase levels in high alcohol intake<br>Aspartate aminotransferase platelet ratio index in high alcohol intake<br>Aspartate transaminase levels in high alcohol intake<br>Platelet count<br>Protein quantitative trait loci (liver) *(HSD17B13)*<br>Sex hormone-binding globulin levels<br>Sex hormone-binding globulin levels adjusted for BMI
5:1188285	rs11133665	Glycine, Histidine, Threonine, Tryptophan, Tyrosine	rs11133665<br>Urinary metabolite levels in chronic kidney disease *(3-hydroxykynurenine)*<br>Urinary metabolite levels in chronic kidney disease *(kynurenine)*<br>Urinary metabolite levels in chronic kidney disease *(phenylalanine)*<br>Urinary metabolite levels in chronic kidney disease *(tryptophan)*<br>Urinary metabolite levels in chronic kidney disease *(tyrosine)*<br>Urinary metabolites *(histidine/τ-methylhistidine)*		rs111306756<br>1-methylhistidine levels<br>rs11133665<br>1-methylhistidine levels<br>Methionine sulfone levels<br>Plasma methionine sulfone levels in chronic kidney disease<br>rs11750211<br>3-methoxytyrosine levels<br>Estimated glomerular filtration rate (creatinine)

Chr:Pos	Rsid	Metabolite	Previously associated urinary metabolite traits	Previously associated blood metabolite traits	Previously associated other traits
			Urine 6-bromotryptophan levels in chronic kidney disease<br>Urine N-acetyl-1-methylhistidine* levels in chronic kidney disease<br>Urine asparagine levels in chronic kidney disease<br>Urine histidine levels in chronic kidney disease<br>Urine kynurenine levels in chronic kidney disease<br>Urine methionine sulfoxide levels in chronic kidney disease<br>Urine phenylalanine levels in chronic kidney disease<br>Urine tryptophan levels in chronic kidney disease<br>Urine tyrosine levels in chronic kidney disease<br>rs11750211<br>Urine 3-hydroxykynurenine levels in chronic kidney disease		
5:34993215	rs11744796	3-Aminoisobutyrate			rs10941225<br>Menarche (age at onset)<br>rs11744796<br>3-aminoisobutyrate levels<br>rs37440<br>Protein quantitative trait loci (liver) *(AGXT2)*
5:35003112	rs468327	3-Aminoisobutyrate	rs468327<br>Urinary metabolites (H-NMR features) *(3.0275, Aminoisobutyrate)*		
5:35037115	rs37369	3-Aminoisobutyrate	rs37369<br>Urinary metabolite levels in chronic kidney disease *(3-aminoisobutyrate)*<br>Urinary metabolite levels in chronic kidney disease *(5-aminovalerate)*<br>Urinary metabolite levels in chronic kidney disease *(X - 12096)*<br>Urinary metabolite levels in chronic kidney disease *(X - 12097)*<br>Urinary metabolite levels in chronic	rs37369<br>Serum metabolite levels *(3-aminoisobutyrate)*	rs37369<br>3-aminoisobutyrate levels<br>Beta-aminoisobutyric acid levels *(Hispanic)*<br>DMGV levels<br>Dimethylarginine (sdma + adma) levels<br>Dimethylguanido valerate levels in chronic kidney disease<br>Metabolite peak levels (QI10969) *(African American or Afro-Caribbean)*<br>Plasma 3-aminoisobutyrate levels in chronic kidney disease<br>Plasma X-12117 levels in chronic kidney disease<br>Plasma X-24518 levels in chronic kidney disease

Chr:Pos	Rsid	Metabolite	Previously associated urinary metabolite traits	Previously associated blood metabolite traits	Previously associated other traits
			kidney disease *(X - 12117)*<br>Urinary metabolite levels in chronic kidney disease *(X - 24387)*<br>Urinary metabolite levels in chronic kidney disease *(X - 24518)*<br>Urinary metabolite modules (eigenmetabolites) in chronic kidney disease *(3-aminoisobutyrate, 5-aminovalerate, X-12096, X-12097, X-12117, X-24518)*<br>Urinary metabolites (H-NMR features) *(1.1975, Aminoisobutyrate)*<br>Urinary metabolites (H-NMR features) *(1.2025, Aminoisobutyrate)*<br>Urinary metabolites (H-NMR features) *(2.6075, Aminoisobutyrate)*<br>Urinary metabolites (H-NMR features) *(2.6125, Aminoisobutyrate)*<br>Urinary metabolites (H-NMR features) *(2.6275, Aminoisobutyrate)*<br>Urinary metabolites (H-NMR features) *(3.0975, Aminoisobutyrate)*<br>Urinary metabolites (H-NMR features) *(3.1075, Aminoisobutyrate)*<br>Urinary metabolites *(1.171 ppm/1.973 ppm)*<br>Urinary metabolites *(3-Aminoisobutyrate concentration)*<br>Urinary metabolites *(3-aminoisobutyrate)*<br>Urine 3-aminoisobutyrate levels in chronic kidney disease<br>Urine 5-aminovalerate levels in chronic kidney disease<br>Urine N2,N2-dimethylguanosine levels in chronic kidney disease<br>Urine X-12096 levels in chronic		Plasma dimethylarginine (SDMA + ADMA) levels in chronic kidney disease<br>Protein quantitative trait loci (liver) *(AGXT2)*<br>Serum dimethylarginine levels (asymmetric/symetric ratio)<br>Symmetric dimethylarginine levels<br>Symmetric dimethylarginine levels in chronic kidney disease<br>Symmetrical dimethylarginine levels<br>X-12117 levels<br>X-24518 levels

Chr:Pos	Rsid	Metabolite	Previously associated urinary metabolite traits	Previously associated blood metabolite traits	Previously associated other traits
			kidney disease<br>Urine X-12097 levels in chronic kidney disease<br>Urine X-12125 levels in chronic kidney disease<br>Urine X-24387 levels in chronic kidney disease<br>Urine dimethylarginine (SDMA + ADMA) levels in chronic kidney disease		
5:35039437	rs2279651	3-Aminoisobutyrate			rs2279651<br>X-24518 levels<br>rs6892674<br>3-aminoisobutyrate levels<br>Protein quantitative trait loci (liver) *(AGXT2)*
5:36219710	rs138373837	3-Aminoisobutyrate			rs138373837<br>Alanine levels<br>Alanine levels (UKB data field 23460)<br>Lysine levels<br>Pyruvate levels<br>Sex hormone-binding globulin levels<br>X-12681 levels
5:150702299	rs61067578	Glycine			rs67910448<br>Glutamine degradant levels
5:150708711	rs147000073	Glycine	rs149235996<br>Urine glycine levels in chronic kidney disease		rs146767775<br>Glutamine degradant levels<br>rs147000073<br>Estimated glomerular filtration rate (creatinine)<br>rs149235996<br>Acetylcarnitine levels<br>Carnitine levels<br>rs77010315<br>Carnitine levels<br>Hexanoylcarnitine levels (Biocrates platform)<br>Octanoylcarnitine levels<br>Propionylcarnitine levels<br>X-11381 levels<br>X-24801 levels
6:111492119	rs241768	Tyrosine			rs1216019<br>Tryptophan levels

Chr:Pos	Rsid	Metabolite	Previously associated urinary metabolite traits	Previously associated blood metabolite traits	Previously associated other traits
					rs241768<br>Tyrosine levels<br>rs9400467<br>Tyrosine levels
7:17287998	rs2106727	Quinic acid, Trigonelline		rs4410790<br>Blood urea nitrogen levels<br>rs6968865<br>Blood urea nitrogen levels	rs2106727<br>1-methylxanthine levels<br>Coffee consumption (cups per day)<br>Serum creatinine levels<br>Sex hormone-binding globulin levels adjusted for BMI<br>Total bilirubin levels<br>Triglyceride levels<br>rs4410790<br>Bitter beverage consumption<br>Bitter non-alcoholic beverage consumption<br>Body mass index<br>Caffeine consumption<br>Caffeine consumption from coffee<br>Caffeine consumption from coffee or tea<br>Caffeine consumption from tea<br>Caffeine levels<br>Caffeine metabolism (plasma 1,3,7-trimethylxanthine (caffeine) level)<br>Caffeine metabolism (plasma 1,7-dimethylxanthine (paraxanthine) to 1,3,7-trimethylxanthine (caffeine) ratio)<br>Coffee consumption<br>Coffee consumption (cups per day)<br>Estimated glomerular filtration rate<br>HDL cholesterol levels<br>Microalbuminuria<br>Non-HDL cholesterol levels<br>Tea consumption<br>Triglyceride levels<br>Urinary albumin excretion<br>Urinary albumin excretion (no hypertensive medication)<br>Urinary albumin-to-creatinine ratio<br>Urinary albumin-to-creatinine ratio *(EA)*<br>Urinary potassium excretion *(EA)*<br>Urinary sodium excretion *(EA)*<br>rs6968554<br>Body mass index<br>Body mass index (MTAG)<br>Caffeine levels<br>Caffeine metabolism (plasma 1,3,7-trimethylxanthine (caffeine) level)<br>Caffeine metabolism (plasma 1,7-dimethylxanthine (paraxanthine) to

Chr:Pos	Rsid	Metabolite	Previously associated urinary metabolite traits	Previously associated blood metabolite traits	Previously associated other traits
					1,3,7-trimethylxanthine (caffeine) ratio)<br>Coffee consumption<br>Coffee consumption (cups per day)<br>Estimated glomerular filtration rate<br>Hemoglobin A1c levels<br>High density lipoprotein cholesterol levels<br>rs6968865<br>Coffee consumption<br>Coffee consumption (cups per day)<br>Coffee max liking<br>Estimated glomerular filtration rate (creatinine)<br>F-chocolate/coffee liking (derived food-liking factor)<br>Low density lipoprotein cholesterol levels<br>Triglyceride levels<br>Triglyceride levels in non-type 2 diabetes
8:18272377	rs4921913	Formate	rs1495741<br>Urinary metabolite levels in chronic kidney disease *(1-methylxanthine)*<br>rs35246381<br>Urinary metabolite levels in chronic kidney disease *(5-acetylamino-6-amino-3-methyluracil)*<br>Urinary metabolite levels in chronic kidney disease *(5-acetylamino-6-formylamino-3-methyluracil)*<br>Urinary metabolite levels in chronic kidney disease *(N-acetylputrescine)*<br>Urinary metabolite modules (eigenmetabolites) in chronic kidney disease *(4-acetamidobutanoate, allo-threonine, N-acetylputrescine)*<br>Urinary metabolites *(2.159 ppm/3.324 ppm)*<br>Urine 5-acetylamino-6-amino-3-methyluracil levels in chronic kidney disease<br>Urine 5-acetylamino-6-formylamino-3-methyluracil levels in chronic kidney disease<br>Urine N-acetylputrescine levels in chronic kidney disease<br>rs4921914<br>Urinary metabolite levels in chronic	rs146812806<br>Serum metabolite levels *(5-acetylamino-6-amino-3-methyluracil)*<br>rs35246381<br>Serum metabolite levels *(5-acetylamino-6-formylamino-3-methyluracil)*<br>rs35570672<br>Serum metabolite levels *(1-methylxanthine)*<br>Serum metabolite levels *(N-acetylputrescine)*<br>rs4921913<br>Serum metabolite levels *(4-acetamidobutanoate)*	rs11784251<br>N-acetylputrescine levels in chronic kidney disease<br>Triglyceride levels<br>rs146812806<br>Mean corpuscular hemoglobin concentration<br>rs1495741<br>1-methylurate levels<br>4-acetamidobutanoate levels in chronic kidney disease<br>5-acetylamino-6-amino-3-methyluracil levels<br>5-acetylamino-6-formylamino-3-methyluracil levels<br>5-acetylamino-6-formylamino-3-methyluracil levels in elite athletes<br>Apolipoprotein B levels<br>Bladder cancer<br>Cholesterol in chylomicrons and extremely large VLDL (UKB data field 23484)<br>Cholesterol levels in chylomicrons and extremely large VLDL<br>Cholesterol to total lipids ratio in medium HDL<br>Cholesterol to total lipids ratio in small HDL<br>Cholesterol, total<br>Cholesteryl ester levels in chylomicrons and extremely large VLDL<br>Cholesteryl esters in chylomicrons and extremely large VLDL (UKB data field 23485)<br>Cholesteryl esters to total lipids ratio in large HDL<br>Cholesteryl esters to total lipids ratio in medium HDL<br>Cholesteryl esters to total lipids ratio in small HDL<br>Cholesteryl esters to total lipids ratio in small LDL<br>Cholesteryl esters to total lipids ratio in very large HDL<br>Concentration of chylomicrons and extremely large VLDL particles

Chr:Pos	Rsid	Metabolite	Previously associated urinary metabolite traits	Previously associated blood metabolite traits	Previously associated other traits
			kidney disease *(1-methylurate)*<br>Urinary metabolite levels in chronic kidney disease *(X - 12410)*<br>Urinary metabolites (H-NMR features) *(2.1875, Unknown)*<br>Urinary metabolites *(Formate/succinate ratio)*<br>Urine 4-acetamidobutanoate levels in chronic kidney disease<br>Urine X-12410 levels in chronic kidney disease<br>rs4921915<br>Urinary metabolite levels in chronic kidney disease *(4-acetamidobutanoate)*<br>Urine N-acetyl-cadaverine levels in chronic kidney disease		Free cholesterol in chylomicrons and extremely large VLDL (UKB data field 23486)<br>Free cholesterol levels in chylomicrons and extremely large VLDL<br>Free cholesterol to total lipids ratio in large LDL<br>Free cholesterol to total lipids ratio in medium LDL<br>Free cholesterol to total lipids ratio in small LDL<br>LDL cholesterol levels<br>Liver injury in anti-tuberculosis drug treatment *(recessive)*<br>Low density lipoprotein cholesterol levels<br>Medication use (HMG CoA reductase inhibitors)<br>Medication use for hyperlipidemia (number of purchases)<br>Monounsaturated fatty acid levels<br>Monounsaturated fatty acids levels (UKB data field 23447)<br>N-acetylputrescine levels in chronic kidney disease<br>N-acetylputrescine levels in elite athletes<br>Non-HDL cholesterol levels<br>Phospholipids to total lipids ratio in large HDL<br>Phospholipids to total lipids ratio in large VLDL<br>Phospholipids to total lipids ratio in medium HDL<br>Phospholipids to total lipids ratio in small HDL<br>Phospholipids to total lipids ratio in small LDL<br>Polyunsaturated fatty acids to monounsaturated fatty acids ratio (UKB data field 23458)<br>Polyunsaturated fatty acids to total fatty acids percentage (UKB data field 23453)<br>Ratio of monounsaturated fatty acids to total fatty acids<br>Ratio of omega-6 fatty acids to total fatty acids<br>Ratio of polyunsaturated fatty acids to monounsaturated fatty acids<br>Ratio of polyunsaturated fatty acids to total fatty acids<br>Saturated fatty acid levels<br>Saturated fatty acids levels (UKB data field 23448)<br>Total cholesterol levels<br>Total cholesterol levels *(EA)*<br>Total fatty acid levels<br>Total fatty acids levels (UKB data field 23442)<br>Total lipid levels in chylomicrons and extremely large VLDL<br>Triglyceride levels<br>Triglyceride levels in HDL<br>Triglyceride levels in IDL<br>Triglyceride levels in LDL<br>Triglyceride levels in large HDL<br>Triglyceride levels in large LDL<br>Triglyceride levels in medium HDL<br>Triglyceride levels in medium LDL

Chr:Pos	Rsid	Metabolite	Previously associated urinary metabolite traits	Previously associated blood metabolite traits	Previously associated other traits
					Triglyceride levels in small LDL<br>Triglyceride levels in very large HDL<br>Triglyceride levels in very small VLDL<br>Triglycerides<br>Triglycerides *(EA)*<br>Triglycerides in IDL (UKB data field 23529)<br>Triglycerides in LDL (UKB data field 23409)<br>Triglycerides in medium LDL (UKB data field 23543)<br>Triglycerides in small LDL (UKB data field 23550)<br>Triglycerides in very large HDL (UKB data field 23557)<br>Triglycerides to total lipids ratio in large HDL<br>Triglycerides to total lipids ratio in medium HDL<br>Triglycerides to total lipids ratio in medium LDL<br>Triglycerides to total lipids ratio in small HDL<br>Triglycerides to total lipids ratio in small LDL<br>Triglycerides to total lipids ratio in very large HDL<br>Youthful appearance (self-reported)<br>rs1495745<br>C-reactive protein levels<br>Cholesteryl esters to total lipids ratio in very small VLDL<br>rs1495747<br>C-reactive protein levels (MTAG)<br>Calcium levels<br>N-acetylputrescine levels<br>rs35246381<br>4-acetamidobutanoate levels<br>Cholesterol to total lipids ratio in large HDL<br>Cholesterol to total lipids ratio in medium VLDL<br>Cholesterol to total lipids ratio in small VLDL<br>Cholesterol to total lipids ratio in very small VLDL<br>Cholesteryl esters to total lipids ratio in medium VLDL<br>Free cholesterol to total lipids in large LDL percentage (UKB data field 23617)<br>Free cholesterol to total lipids in small LDL percentage (UKB data field 23627)<br>Free cholesterol to total lipids in small VLDL percentage (UKB data field 23602)<br>Free cholesterol to total lipids ratio in IDL<br>Free cholesterol to total lipids ratio in medium VLDL<br>Free cholesterol to total lipids ratio in small VLDL<br>Free cholesterol to total lipids ratio in very small VLDL<br>Mean corpuscular hemoglobin concentration<br>N-acetylputrescine levels<br>Phosphatidylcholine levels

Chr:Pos	Rsid	Metabolite	Previously associated urinary metabolite traits	Previously associated blood metabolite traits	Previously associated other traits
					Phosphoglycerides levels<br>Phospholipid levels in small HDL<br>Phospholipids to total lipids ratio in small VLDL<br>Plasma 5-acetylamino-6-amino-3-methyluracil levels in chronic kidney disease<br>Plasma N-acetylputrescine levels in chronic kidney disease<br>Triglyceride levels<br>Triglyceride levels (MTAG)<br>Triglyceride levels x short total sleep time interaction (2df test)<br>Triglycerides in very small VLDL (UKB data field 23522)<br>Triglycerides to total lipids in small LDL percentage (UKB data field 23628)<br>Triglycerides to total lipids in very small VLDL percentage (UKB data field 23608)<br>Triglycerides to total lipids ratio in medium VLDL<br>Triglycerides to total lipids ratio in small VLDL<br>Triglycerides to total lipids ratio in very small VLDL<br>Triglycerides x physical activity interaction (2df test)<br>rs35570672<br>1-methylurate levels<br>1-methylxanthine levels<br>Mean corpuscular hemoglobin<br>Total cholesterol levels<br>Triglyceride levels<br>X-12410 levels<br>rs4921913<br>1-methylxanthine levels<br>4-acetamidobutanoate levels<br>4-acetamidobutanoate-to-N1-methyladenosine ratio<br>5-acetylamino-6-amino-3-methyluracil levels<br>5-acetylamino-6-formylamino-3-methyluracil levels<br>Average diameter for VLDL particles<br>Cholesterol levels in large VLDL<br>Cholesterol levels in very large VLDL<br>Cholesteryl ester levels in large VLDL<br>Cholesteryl ester levels in very large VLDL<br>Concentration of chylomicrons and extremely large VLDL particles (UKB data field 23481)<br>Concentration of large VLDL particles<br>Concentration of small VLDL particles<br>Concentration of very large VLDL particles<br>Free cholesterol levels in VLDL<br>Free cholesterol levels in large VLDL<br>Liver enzyme levels (gamma-glutamyl transferase)

Chr:Pos	Rsid	Metabolite	Previously associated urinary metabolite traits	Previously associated blood metabolite traits	Previously associated other traits
					Low-density lipoprotein levels (MTAG)
					Multi-trait sum score
					N-acetylputrescine levels
					Omega-6 fatty acid levels
					Phospholipid levels in VLDL
					Phospholipid levels in large VLDL
					Phospholipid levels in very large VLDL
					Phospholipids in chylomicrons and extremely large VLDL (UKB data field 23483)
					Total lipid levels in VLDL
					Total lipid levels in large VLDL
					Total lipid levels in small VLDL
					Total lipid levels in very large VLDL
					Total lipids in chylomicrons and extremely large VLDL (UKB data field 23482)
					Triglyceride levels
					Triglyceride levels in VLDL
					Triglyceride levels in chylomicrons and extremely large VLDL
					Triglyceride levels in large VLDL
					Triglyceride levels in medium VLDL
					Triglyceride levels in very large VLDL
					Triglycerides in chylomicrons and extremely large VLDL (UKB data field 23487)
					rs4921914
					1-methylxanthine levels
					5-acetylamino-6-amino-3-methyluracil levels
					5-acetylamino-6-formylamino-3-methyluracil levels
					Mean corpuscular hemoglobin concentration
					N-acetylputrescine levels
					Total cholesterol levels
					Triglyceride levels *(Trans-ethnic initial)*
					rs4921915
					Albumin levels
					Cholesterol to total lipids ratio in very large VLDL
					Cholesteryl esters to total lipids ratio in very large VLDL
					Free cholesterol levels in very large VLDL
					Free cholesterol to total lipids in medium LDL percentage (UKB data field 23622)
					Iron status biomarkers (transferrin levels)
					Phospholipid levels in chylomicrons and extremely large VLDL
					Plasma X-12410 levels in chronic kidney disease
					Polyunsaturated fatty acid levels
					Ratio of triglycerides to phosphoglycerides
					Total cholesterol levels

Chr:Pos	Rsid	Metabolite	Previously associated urinary metabolite traits	Previously associated blood metabolite traits	Previously associated other traits
					Total lipid levels in small HDL<br>Total triglycerides levels<br>Triglyceride levels in small HDL<br>Triglyceride levels in small VLDL<br>Triglycerides
8:74868909	rs72661850	4-Deoxyerythronic acid			rs62509311<br>Liver enzyme levels (alkaline phosphatase)<br>Low density lipoprotein cholesterol levels<br>rs7013120<br>Total cholesterol levels<br>rs72661850<br>Gamma-glutamylthreonine levels<br>rs72661853<br>Serum alkaline phosphatase levels<br>Threonine levels
9:136146597	rs550057	Xylose		rs550057<br>Blood urea nitrogen levels	rs550057<br>Alpha-(1,3)-fucosyltransferase 10 levels<br>Asthma or COVID-19 (pleiotropy)<br>Basal Cell Adhesion Molecule levels<br>Beta-1,4-galactosyltransferase 2 levels<br>Brain morphology (MOSTest)<br>COVID-19 with respiratory failure requiring mechanical ventilation<br>Calcium levels<br>Carbohydrate sulfotransferase 12 levels<br>Carbohydrate sulfotransferase 15 levels<br>Carbohydrate sulfotransferase 15 levels (CHST15.4469.78.2)<br>Cytokine network levels (multivariate analysis)<br>Estimated glomerular filtration rate<br>Estimated glomerular filtration rate (creatinine)<br>Golgi membrane protein 1 levels<br>Gut microbiota abundance (k_Bacteria.p_Actinobacteria.c_Actinobacteria.o_Coriobacteriales)<br>Gut microbiota abundance (k_Bacteria.p_Actinobacteria.c_Actinobacteria.o_Coriobacteriales.f_Coriobacteriaceae)<br>Gut microbiota abundance (k_Bacteria.p_Actinobacteria.c_Actinobacteria.o_Coriobacteriales.f_Coriobacteriaceae.g_Collinsella)<br>Gut microbiota abundance (k_Bacteria.p_Actinobacteria.c_Actinobacteria.o_Coriobacteriales.f_Coriobacteriaceae.g_Collinsella.s_Collinsella_aerofaciens)<br>Hematocrit

Chr:Pos	Rsid	Metabolite	Previously associated urinary metabolite traits	Previously associated blood metabolite traits	Previously associated other traits
					Hemoglobin concentration<br>Inflammatory biomarkers (multivariate analysis)<br>Intestinal-type alkaline phosphatase levels<br>Intestinal-type alkaline phosphatase levels (ALPI.10463.23.3)<br>LDL cholesterol<br>LDL cholesterol levels<br>Low density lipoprotein cholesterol levels<br>Low density lipoprotein cholesterol levels *(EA)*<br>Low density lipoprotein cholesterol levels *(Hispanic)*<br>Lung function (FVC)<br>Pancreatic alpha-amylase levels<br>Pancreatic triacylglycerol lipase levels<br>Peak expiratory flow<br>Protein CASC4 levels<br>Protein FAM177A1 levels<br>Protein FAM177A1 levels (FAM177A1.8039.41.3)<br>Protein FAM3B levels<br>Protein FAM3D levels<br>Protein jagged-1 levels (JAG1.5092.51.3)<br>Red blood cell count<br>Scavenger receptor class F member 2 levels<br>Secreted and transmembrane protein 1 levels<br>Serum creatinine levels<br>Serum levels of protein C1GALT1C1<br>Serum levels of protein CASC4<br>Serum levels of protein CHST15<br>Serum levels of protein FAM177A1<br>Serum levels of protein FAM3D<br>Serum levels of protein GOLM1<br>Serum levels of protein ISLR2<br>Serum levels of protein QSOX2<br>Serum levels of protein TIE1<br>Total cholesterol levels<br>Total cholesterol levels *(EA)*<br>Total cholesterol levels *(Hispanic)*<br>Tumor necrosis factor ligand superfamily member 8 levels<br>Two-hour glucose<br>Tyrosine-protein kinase receptor Tie-1, soluble levels<br>X-26054 levels<br>rs9411378<br>C-C motif chemokine 25 levels (CCL25.2705.5.2)<br>COVID-19 (covid vs negative)<br>Calcium levels<br>Coronary artery disease

Chr:Pos	Rsid	Metabolite	Previously associated urinary metabolite traits	Previously associated blood metabolite traits	Previously associated other traits
					Gamma glutamyl transferase levels<br>Low density lipoprotein cholesterol levels<br>N-acetyl neuraminic acid levels<br>Red cell distribution width<br>Serum levels of protein CHST15<br>Serum levels of protein GNS<br>Total cholesterol levels
12:345369	rs2080403	3-Aminoisobutyrate	rs11613331<br>Urinary metabolite levels in chronic kidney disease *(3-aminoisobutyrate)*<br>Urine 3-aminoisobutyrate levels in chronic kidney disease	rs11613331<br>Serum metabolite levels *(3-aminoisobutyrate)*<br>Serum metabolite levels *(imidazole lactate)*<br>Serum metabolite levels *(pyroglutamine)**	rs11062167<br>Estimated glomerular filtration rate<br>Glomerular filtration rate (creatinine)<br>rs11613331<br>1-ribosyl-imidazoleacetate levels<br>3-hydroxybutyrate + 3-aminoisobutyrate levels<br>Beta-aminoisobutyric acid levels *(Hispanic)*<br>Deoxycarnitine levels in elite athletes<br>Height<br>Imidazole propionate levels<br>Metabolite levels *(beta aminoisobutyric acid)*<br>Pyroglutamine levels<br>rs2080402<br>1-ribosyl-imidazoleacetate levels<br>Photoreceptor cell layer thickness phenotypes (MTAG)<br>X-25420 levels<br>rs3782860<br>Chronotype<br>rs7969761<br>3-aminoisobutyrate levels<br>Betaine-to-pyroglutamine ratio<br>Estimated glomerular filtration rate in non-diabetics<br>Guanidinoacetate levels<br>Imidazole lactate levels<br>Imidazole propionate levels<br>Plasma 3-aminoisobutyrate levels in chronic kidney disease
12:4521511	rs78470967	4-Deoxythreonate			rs78470967<br>Height<br>Type 2 diabetes
12:122344302	rs1795967	2-Hydroxyisobutyrate	rs10840627<br>Urine caffeate levels in chronic kidney disease<br>rs11043222<br>Urine alpha-hydroxyisovalerate	rs11614623<br>Serum metabolite levels *(2-hydroxyphenylacetate)*<br>rs1168674<br>Serum metabolite levels *(beta-*	rs10840627<br>Beta-hydroxyisovalerate levels<br>rs11502392<br>2-hydroxyphenylacetate levels<br>rs1154510

Chr:Pos	Rsid	Metabolite	Previously associated urinary metabolite traits	Previously associated blood metabolite traits	Previously associated other traits
			levels in chronic kidney disease<br>rs1678967<br>Urine 2-hydroxybutyrate/2-hydroxyisobutyrate levels in chronic kidney disease<br>rs2247139<br>Urine 2-hydroxyphenylacetate levels in chronic kidney disease<br>rs2688412<br>Urinary metabolite levels in chronic kidney disease *(4-hydroxyphenylpyruvate)*<br>Urine 4-hydroxyphenylpyruvate levels in chronic kidney disease<br>rs4760099<br>Urinary metabolites *(2-hydroxyisobutyrate)*<br>rs7314056<br>Urinary metabolites (H-NMR features) *(1.3625, 2-Hydroxyisobutyrate)*<br>rs830124<br>Urinary metabolites *(2-Hydroxyisobutyrate concentration)*	*hydroxyisovalerate)*<br>rs1720067<br>Serum metabolite levels *(4-hydroxyphenylpyruvate)*	4-hydroxyphenylpyruvate levels<br>Beta-hydroxyisovalerate levels<br>rs1154515<br>4-hydroxyphenylpyruvate levels<br>rs11614623<br>2-hydroxyphenylacetate levels<br>rs1168674<br>Plasma beta-hydroxyisovalerate levels in chronic kidney disease<br>rs1169075<br>Tyrosine levels<br>rs1667583<br>Total cholesterol levels<br>rs1667584<br>4-hydroxyphenylpyruvate levels<br>rs2247139<br>Low density lipoprotein cholesterol levels<br>rs493519<br>2-hydroxyisobutyrate levels<br>rs7314056<br>Plasma 2-hydroxyphenylacetate levels in chronic kidney disease
16:20557634	rs7499358	3-Hydroxyisovalerate		rs7499271<br>Serum metabolite levels *(Phenylacetate)*<br>Serum metabolite levels *(phenylacetate)*<br>Serum metabolite levels *(picolinate)*	rs58395451<br>Beta-hydroxyisovalerate levels<br>rs7499557<br>Plasma picolinate levels in chronic kidney disease<br>rs9922704<br>X-17676 levels
16:20608891	rs540815683	3-Hydroxyisovalerate			rs185603444<br>X-17676 levels<br>rs192218016<br>Beta-hydroxyisovalerate levels<br>rs193030024<br>Salicylate levels
17:26824156	rs11567842	Citrate		rs11567842<br>Blood urea nitrogen levels	
17:37631883	rs11078902	Threonine	rs7219014<br>Urinary metabolites *(histidine/τ-*		rs10432031<br>Sulfatide (d18:1/16:0) levels

Chr:Pos	Rsid	Metabolite	Previously associated urinary metabolite traits	Previously associated blood metabolite traits	Previously associated other traits
			methylhistidine)<br>rs7220650<br>Urine tryptophan levels in chronic kidney disease<br>Urine tyrosine levels in chronic kidney disease<br>rs755500<br>Urine histidine levels in chronic kidney disease		rs10491128<br>Estimated glomerular filtration rate (creatinine)<br>Estimated glomerular filtration rate in non-diabetics<br>rs10491129<br>Mean reticulocyte volume<br>rs1077715<br>Coronary artery disease<br>rs11078893<br>Estimated glomerular filtration rate<br>rs11078901<br>Serum creatinine levels<br>rs11078916<br>Type 2 diabetes<br>rs113414512<br>Creatinine levels (UKB data field 23478)<br>rs113825099<br>Drinks per week<br>rs12451586<br>Glomerular filtration rate (creatinine)<br>rs12453397<br>Drinks per week<br>rs12600751<br>Asthma (childhood onset)<br>Educational attainment (years of education) *(conditional-joint)*<br>rs12942352<br>1-methylhistidine levels<br>Methionine sulfone levels<br>Sulfatide (d18:1/16:0) levels<br>rs12947281<br>Mean platelet volume<br>rs1619021<br>Red blood cell count<br>rs2100651<br>Mean platelet volume<br>rs2168785<br>Serum creatinine levels<br>rs34473775<br>Brain morphology (MOSTest)<br>rs35497503<br>Refractive error<br>rs3964723<br>Platelet count<br>rs4239222<br>Hemoglobin concentration

Chr:Pos	Rsid	Metabolite	Previously associated urinary metabolite traits	Previously associated blood metabolite traits	Previously associated other traits
					rs4390625<br>Creatinine levels<br>rs4794813<br>Estimated glomerular filtration rate<br>Estimated glomerular filtration rate (creatinine)<br>rs4794814<br>Estimated glomerular filtration rate<br>rs4795364<br>Spherical equivalent or myopia (age of diagnosis)<br>rs4795384<br>Estimated glomerular filtration rate<br>Whole brain restricted directional diffusion (multivariate analysis)<br>rs4795386<br>Free cholesterol to total lipids in very large HDL percentage (UKB data field 23632)<br>rs55722796<br>Drinks per week<br>Estimated glomerular filtration rate (creatinine) *(conditioned on rs35289683, rs533075, rs114777282, rs1031459)*<br>rs6503506<br>Calcium levels<br>rs677888<br>Microalbuminuria<br>Urinary albumin-to-creatinine ratio<br>rs7212715<br>Creatinine levels<br>Glomerular filtration rate<br>rs755500<br>Red blood cell count<br>rs8069451<br>Asthma and cardiovascular disease<br>Childhood asthma with severe exacerbations<br>FEV1<br>Lung function (FVC)<br>Smoking initiation<br>rs8072297<br>Creatinine levels<br>rs8076546<br>Mean corpuscular hemoglobin<br>rs9747342<br>Drinks per week<br>rs9892055<br>Systemic lupus erythematosus (MTAG)

Chr:Pos	Rsid	Metabolite	Previously associated urinary metabolite traits	Previously associated blood metabolite traits	Previously associated other traits
17:37633970	rs12453397	Tryptophan, Tyrosine	rs7219014<br>Urinary metabolites *(histidine/τ-methylhistidine)*<br>rs7220650<br>Urine tryptophan levels in chronic kidney disease<br>Urine tyrosine levels in chronic kidney disease<br>rs755500<br>Urine histidine levels in chronic kidney disease		rs10432031<br>Sulfatide (d18:1/16:0) levels<br>rs10491128<br>Estimated glomerular filtration rate (creatinine)<br>Estimated glomerular filtration rate in non-diabetics<br>rs10491129<br>Mean reticulocyte volume<br>rs1077715<br>Coronary artery disease<br>rs11078893<br>Estimated glomerular filtration rate<br>rs11078901<br>Serum creatinine levels<br>rs11078916<br>Type 2 diabetes<br>rs113414512<br>Creatinine levels (UKB data field 23478)<br>rs113825099<br>Drinks per week<br>rs12451586<br>Glomerular filtration rate (creatinine)<br>rs12453397<br>Drinks per week<br>rs12600751<br>Asthma (childhood onset)<br>Educational attainment (years of education) *(conditional-joint)*<br>rs12942352<br>1-methylhistidine levels<br>Methionine sulfone levels<br>Sulfatide (d18:1/16:0) levels<br>rs12947281<br>Mean platelet volume<br>rs1619021<br>Red blood cell count<br>rs2100651<br>Mean platelet volume<br>rs2168785<br>Serum creatinine levels<br>rs34473775<br>Brain morphology (MOSTest)<br>rs35497503<br>Refractive error<br>rs3964723<br>Platelet count

Chr:Pos	Rsid	Metabolite	Previously associated urinary metabolite traits	Previously associated blood metabolite traits	Previously associated other traits
					rs4239222<br>Hemoglobin concentration<br>rs4390625<br>Creatinine levels<br>rs4794813<br>Estimated glomerular filtration rate<br>Estimated glomerular filtration rate (creatinine)<br>rs4794814<br>Estimated glomerular filtration rate<br>rs4795364<br>Spherical equivalent or myopia (age of diagnosis)<br>rs4795384<br>Estimated glomerular filtration rate<br>Whole brain restricted directional diffusion (multivariate analysis)<br>rs4795386<br>Free cholesterol to total lipids in very large HDL percentage (UKB data field 23632)<br>rs55722796<br>Drinks per week<br>Estimated glomerular filtration rate (creatinine) *(conditioned on rs35289683, rs533075, rs114777282, rs1031459)*<br>rs6503506<br>Calcium levels<br>rs677888<br>Microalbuminuria<br>Urinary albumin-to-creatinine ratio<br>rs7212715<br>Creatinine levels<br>Glomerular filtration rate<br>rs755500<br>Red blood cell count<br>rs8069451<br>Asthma and cardiovascular disease<br>Childhood asthma with severe exacerbations<br>FEV1<br>Lung function (FVC)<br>Smoking initiation<br>rs8072297<br>Creatinine levels<br>rs8076546<br>Mean corpuscular hemoglobin<br>rs9747342<br>Drinks per week

Chr:Pos	Rsid	Metabolite	Previously associated urinary metabolite traits	Previously associated blood metabolite traits	Previously associated other traits
					rs9892055<br>Systemic lupus erythematosus (MTAG)
17:37636695	rs4795371	Histidine	rs7219014<br>Urinary metabolites *(histidine/τ-methylhistidine)*<br>rs7220650<br>Urine tryptophan levels in chronic kidney disease<br>Urine tyrosine levels in chronic kidney disease<br>rs755500<br>Urine histidine levels in chronic kidney disease		rs10432031<br>Sulfatide (d18:1/16:0) levels<br>rs10491128<br>Estimated glomerular filtration rate (creatinine)<br>Estimated glomerular filtration rate in non-diabetics<br>rs10491129<br>Mean reticulocyte volume<br>rs1077715<br>Coronary artery disease<br>rs11078893<br>Estimated glomerular filtration rate<br>rs11078901<br>Serum creatinine levels<br>rs11078916<br>Type 2 diabetes<br>rs113414512<br>Creatinine levels (UKB data field 23478)<br>rs113825099<br>Drinks per week<br>rs12451586<br>Glomerular filtration rate (creatinine)<br>rs12453397<br>Drinks per week<br>rs12600751<br>Asthma (childhood onset)<br>Educational attainment (years of education) *(conditional-joint)*<br>rs12942352<br>1-methylhistidine levels<br>Methionine sulfone levels<br>Sulfatide (d18:1/16:0) levels<br>rs12947281<br>Mean platelet volume<br>rs1619021<br>Red blood cell count<br>rs2100651<br>Mean platelet volume<br>rs2168785<br>Serum creatinine levels<br>rs34473775<br>Brain morphology (MOSTest)<br>rs35497503

Chr:Pos	Rsid	Metabolite	Previously associated urinary metabolite traits	Previously associated blood metabolite traits	Previously associated other traits
					Refractive error<br>rs3964723<br>Platelet count<br>rs4239222<br>Hemoglobin concentration<br>rs4390625<br>Creatinine levels<br>rs4794813<br>Estimated glomerular filtration rate<br>Estimated glomerular filtration rate (creatinine)<br>rs4794814<br>Estimated glomerular filtration rate<br>rs4795364<br>Spherical equivalent or myopia (age of diagnosis)<br>rs4795384<br>Estimated glomerular filtration rate<br>Whole brain restricted directional diffusion (multivariate analysis)<br>rs4795386<br>Free cholesterol to total lipids in very large HDL percentage (UKB data field 23632)<br>rs55722796<br>Drinks per week<br>Estimated glomerular filtration rate (creatinine) *(conditioned on rs35289683, rs533075, rs114777282, rs1031459)*<br>rs6503506<br>Calcium levels<br>rs677888<br>Microalbuminuria<br>Urinary albumin-to-creatinine ratio<br>rs7212715<br>Creatinine levels<br>Glomerular filtration rate<br>rs755500<br>Red blood cell count<br>rs8069451<br>Asthma and cardiovascular disease<br>Childhood asthma with severe exacerbations<br>FEV1<br>Lung function (FVC)<br>Smoking initiation<br>rs8072297<br>Creatinine levels<br>rs8076546<br>Mean corpuscular hemoglobin

Chr:Pos	Rsid	Metabolite	Previously associated urinary metabolite traits	Previously associated blood metabolite traits	Previously associated other traits
					rs9747342<br>Drinks per week<br>rs9892055<br>Systemic lupus erythematosus (MTAG)

Supplementary Table 3

MAGMA tissue enrichment analysis, tissues with p<0.01. The analysis was performed using MAGMA gene-property analysis for tissue specificity (one-sided p-value).

Metabolite	Tissue	N genes	Beta	SE	P
cis-Aconitate	Adipose Tissue	17391	0.049	0.013	6.9E-05
glycine	Kidney	17393	0.033	0.009	1.5E-04
Pyroglutamate	Pituitary	17392	0.025	0.008	6.8E-04
Allantoin	Cervix Uteri	17390	0.042	0.013	0.0010
2-Furoylglycine	Testis	17391	0.016	0.005	0.0014
Mannitol	Small Intestine	17387	0.030	0.010	0.0016
Acetate	Esophagus	17393	0.048	0.016	0.0018
Pseudouridine	Blood Vessel	17393	0.031	0.011	0.0030
3-(3-Hydroxyphenyl)-3-hydroxypropionic acid	Adipose Tissue	17390	0.034	0.013	0.0042
3-(3-Hydroxyphenyl)-3-hydroxypropionic acid	Breast	17390	0.039	0.015	0.0049
cis-Aconitate	Blood Vessel	17391	0.028	0.011	0.0057
Quinic acid	Ovary	17392	0.024	0.010	0.0067
Allantoin	Thyroid	17390	0.025	0.010	0.0071
2-Hydroxyisobutyrate	Muscle	17393	0.016	0.007	0.0094
Proline betaine	Blood	17390	0.014	0.006	0.0094

Supplementary Table 4

Mendelian randomization analysis outcomes and exposures. Two sample Mendelian randomization analysis outcomes (n=17) used with urinary metabolites as the exposures, and exposures (n=3) used with urinary metabolites as the outcomes.

Analysis	Source	Trait	Population	PMID	IEU GWAS database ID
**Metabolite -> Outcome**	DNCRI	allvcntrl	European	31537649	–
		ckddn	European	31537649	–
		ckdextreme	European	31537649	–
		ckd	European	31537649	–
		DN	European	31537649	–
		esrd	European	31537649	–
		esrdvall	European	31537649	–
		esrdvmacro	European	31537649	–
		macro	European	31537649	–
		micro	European	31537649	–
	CKDGen	eGFR	European	31152163	–
		CKD	European	31152163	–
		UACR	European	31511532	–
	DIAMANTE	T2D	European	35551307	–
	IEU GWAS database	Asthma	European	–	finn-b-J10_ASTHMA
		Body mass index (BMI)	European	–	ukb-b-19953
		Coronary heart disease	Mixed	26343387	ieu-a-7
**Exposure -> Metabolite**	CKDGen	eGFR	European	31152163	–
		UACR	European	31511532	–
	IEU GWAS database	BMI	European	-	ukb-b-19953

Supplementary Table 5

FinnDiane physicians and nurses participating in the collection of the FinnDiane study subjects.

**The Finnish Diabetic Nephropathy Study Centers**	
Anjalankoski Health Center	S.Koivula, T.Uggeldahl
Central Finland Central Hospital, Jyväskylä	T.Forslund, A.Halonen, A.Koistinen, P.Koskiaho, M.Laukkanen, J.Saltevo, M.Tiihonen
Central Hospital of Åland Islands, Mariehamn	M.Forsen, H.Granlund, A.-C.Jonsson, B.Nyroos
Central Hospital of Kanta-Häme, Hämeenlinna	P.Kinnunen, A.Orvola, T.Salonen, A.Vähänen
Central Hospital of Kymenlaakso, Kotka	R.Paldanius, M.Riihelä, L.Ryysy
Central Hospital of Länsi-Pohja, Kemi	H.Laukkanen, P.Nyländen, A.Sademies
Central Ostrobothnian Hospital District, Kokkola	S.Anderson, B.Asplund, U.Byskata, P.Liedes, M.Kuusela, T.Virkkala
City of Espoo Health Center:	
Espoonlahti	A.Nikkola, E.Ritola
Tapiola	M.Niska, H.Saarinen
Samaria	E.Oukko-Ruponen, T.Virtanen
Viherlaakso	A.Lyytinen
City of Helsinki Health Center:	
Puistola	H.Kari, T.Simonen
Suutarila	A.Kaprio, J.Kärkkäinen, B.Rantaeskola
Töölö	P.Kääriäinen, J.Haaga, A-L.Pietiläinen
City of Hyvinkää Health Center	S.Klemetti, T.Nyandoto, E.Rontu, S.Satuli-Autere
City of Vantaa Health Center:	
Korso	R.Toivonen, H.Virtanen
Länsimäki	R.Ahonen, M.Ivaska-Suomela, A.Jauhiainen
Martinlaakso	M.Laine, T.Pellonpää, R.Puranen
Myyrmäki	A.Airas, J.Laakso, K.Rautavaara
Rekola	M.Erola, E.Jatkola
Tikkurila	R.Lönnblad, A.Malm, J.Mäkelä, E.Rautamo
Heinola Health Center	P.Hentunen, J.Lagerstam
Helsinki University Central Hospital, Department of Medicine, Division of Nephrology	A.Ahola, M.Feodoroff, D.Gordin, O.Heikkilä, K.Hietala, M.Korolainen J.Kytö, S.Lindh, K.Pettersson-Fernholm, A.Sandelin, L.Thorn, J.Tuomikangas, T.Vesisenaho, J.Wadén
Herttoniemi Hospital, Helsinki	V.Sipilä
Hospital of Lounais-Häme, Forssa	T.Kalliomäki, J.Koskelainen, R.Nikkanen, N.Savolainen, H.Sulonen, E.Valtonen
Hyvinkää Hospital	L. Norvio, A. Hämäläinen
Iisalmi Hospital	E.Toivanen

Jokilaakso Hospital, Jämsä	A.Parta, I.Pirttiniemi
Jorvi Hospital, Helsinki University Central Hospital	S.Aranko, S.Ervasti, R.Kauppinen-Mäkelin, A.Kuusisto, T.Leppälä, K.Nikkilä, L.Pekkonen
Jyväskylä Health Center, Kyllö	K.Nuorva, M.Tiihonen
Kainuu Central Hospital, Kajaani	S.Jokelainen, K.Kananen, M.Karjalainen, P.Kemppainen, A-M.Mankinen, A.Reponen, M.Sankari
Kerava Health Center	H.Stuckey, P.Suominen
Kirkkonummi Health Center	A.Lappalainen, M.Liimatainen, J.Santaholma
Kivelä Hospital, Helsinki	A.Aimolahti, E.Huovinen
Koskela Hospital, Helsinki	V.Ilkka, M.Lehtimäki
Kotka Health Center	E.Pälikkö-Kontinen, A.Vanhanen
Kouvola Health Center	E.Koskinen, T.Siitonen
Kuopio University Hospital	E.Huttunen, R.Ikäheimo, P.Karhapää, P.Kekäläinen, M.Laakso, T.Lakka, E.Lampainen, L.Moilanen, L.Niskanen, U.Tuovinen, I.Vauhkonen, E.Voutilainen
Kuusamo Health Center	T.Kääriäinen, E.Isopoussu
Kuusankoski Hospital	E.Kilkki, I.Koskinen, L.Riihelä
Laakso Hospital, Helsinki	T.Meriläinen, P.Poukka, R.Savolainen, N.Uhlenius
Lahti City Hospital	A.Mäkelä, M.Tanner
Lapland Central Hospital, Rovaniemi	L.Hyvärinen, K.Lampela, S.Pöykkö, T.Rompasaari, S.Severinkangas, T.Tulokas
Lappeenranta Health Center	P. Erola, L. Härkönen, P.Linkola, I.Pulli, E.Repo
Lohja Hospital	T.Granlund, K.Hietanen, M.Porrassalmi, M.Saari, T.Salonen, M.Tiikkainen,
Länsi-Uusimaa Hospital, Tammisaari	I.-M.Jousmaa, J.Rinne
Loimaa Health Center	A.Mäkelä, P.Eloranta
Malmi Hospital, Helsinki	H.Lanki, S.Moilanen, M.Tilly-Kiesi
Mikkeli Central Hospital	A.Gynther, R.Manninen, P.Nironen, M.Salminen, T.Vänttinen
Mänttä Regional Hospital	I.Pirttiniemi, A-M.Hänninen
North Karelian Hospital, Joensuu	U-M.Henttula, P.Kekäläinen, M.Pietarinen, A.Rissanen, M.Voutilainen
Nurmijärvi Health Center	A.Burgos, K.Urtamo
Oulaskangas Hospital, Oulainen	E.Jokelainen, P-L.Jylkkä, E.Kaarlela, J.Vuolaspuro
Oulu Health Center	L.Hiltunen, R.Häkkinen, S.Keinänen-Kiukaanniemi
Oulu University Hospital	R.Ikäheimo
Päijät-Häme Central Hospital	H.Haapamäki, A.Helanterä, S.Hämäläinen, V.Ilvesmäki, H.Miettinen
Palokka Health Center	P.Sopanen, L.Welling
Pieksämäki Hospital	V.Sevtsenko, M.Tamminen
Pietarsaari Hospital	M-L.Holmbäck, B.Isomaa, L.Sarelin
Pori City Hospital	P.Ahonen, P.Merisalo, E.Muurinen, K.Sävelä
Porvoo Hospital	M.Kallio, B.Rask, S.Rämö

Raahe Hospital	A.Holma, M.Honkala, A.Tuomivaara, R.Vainionpää
Rauma Hospital	K.Laine, K.Saarinen, T.Salminen
Riihimäki Hospital	P.Aalto, E.Immonen, L.Juurinen
Salo Hospital	A.Alanko, J.Lapinleimu, P.Rautio, M.Virtanen
Satakunta Central Hospital, Pori	M.Asola, M.Juhola, P.Kunelius, M.-L.Lahdenmäki, P.Pääkkönen, M.Rautavirta
Savonlinna Central Hospital	T.Pulli, P.Sallinen, M.Taskinen, E.Tolvanen, T.Tuominen, H.Valtonen, A.Vartia, S-L.Viitanen
Seinäjoki Central Hospital	O.Antila, E.Korpi-Hyövälti, T.Latvala, E.Leijala, T.Leikkari, M.Punkari N.Rantamäki, H.Vähävuori
South Karelia Central Hospital, Lappeenranta	T.Ensala, E.Hussi, R.Härkönen, U.Nyholm, J.Toivanen
Tampere Health Center	A.Vaden, P.Alarotu, E.Kujansuu, H.Kirkkopelto-Jokinen, M.Helin, S.Gummerus, L.Calonius, T.Niskanen, T.Kaitala, T.Vatanen
Tampere University Hospital	I.Ala-Houhala, R.Kannisto, T.Kuningas, P.Lampinen, M.Määttä, H.Oksala, T.Oksanen, A.Putila, H.Saha, K.Salonen, H.Tauriainen, S.Tulokas
Tiirismaa Health Center, Hollola	T.Kivelä, L.Petlin, L.Savolainen
Turku Health Center	A.Artukka, I.Hämäläinen, L.Lehtinen, E.Pyysalo, H.Virtamo, M.Viinikkala, M.Vähätalo
Turku University Central Hospital	K.Breitholz, R.Eskola, K.Metsärinne, U.Pietilä, P.Saarinen, R.Tuominen, S.Äyräpää
Vaajakoski Health Center	K.Mäkinen, P.Sopanen
Valkeakoski Regional Hospital	S.Ojanen, E.Valtonen, H.Ylönen, M.Rautiainen, T.Immonen
Vammala Regional Hospital	I.Isomäki, R.Kroneld, L.Mustaniemi, M.Tapiolinna-Mäkelä
Vasa Central Hospital	S.Bergkulla, U.Hautamäki, V-A.Myllyniemi, I.Rusk

Supplementary Figure 1

Heritability estimates for the urinary metabolites. The metabolites are order by the meta-analysis estimates of heritability. Full circle indicates p-value < 0.05 and * heterogeneity test p-value < 0.05 (Supplementary Table 4). The meta-analysis p-value was calculated applying a random-effects model utilizing the inverse variance method and the between study heterogeneity p-value was calculated with a Q-test.

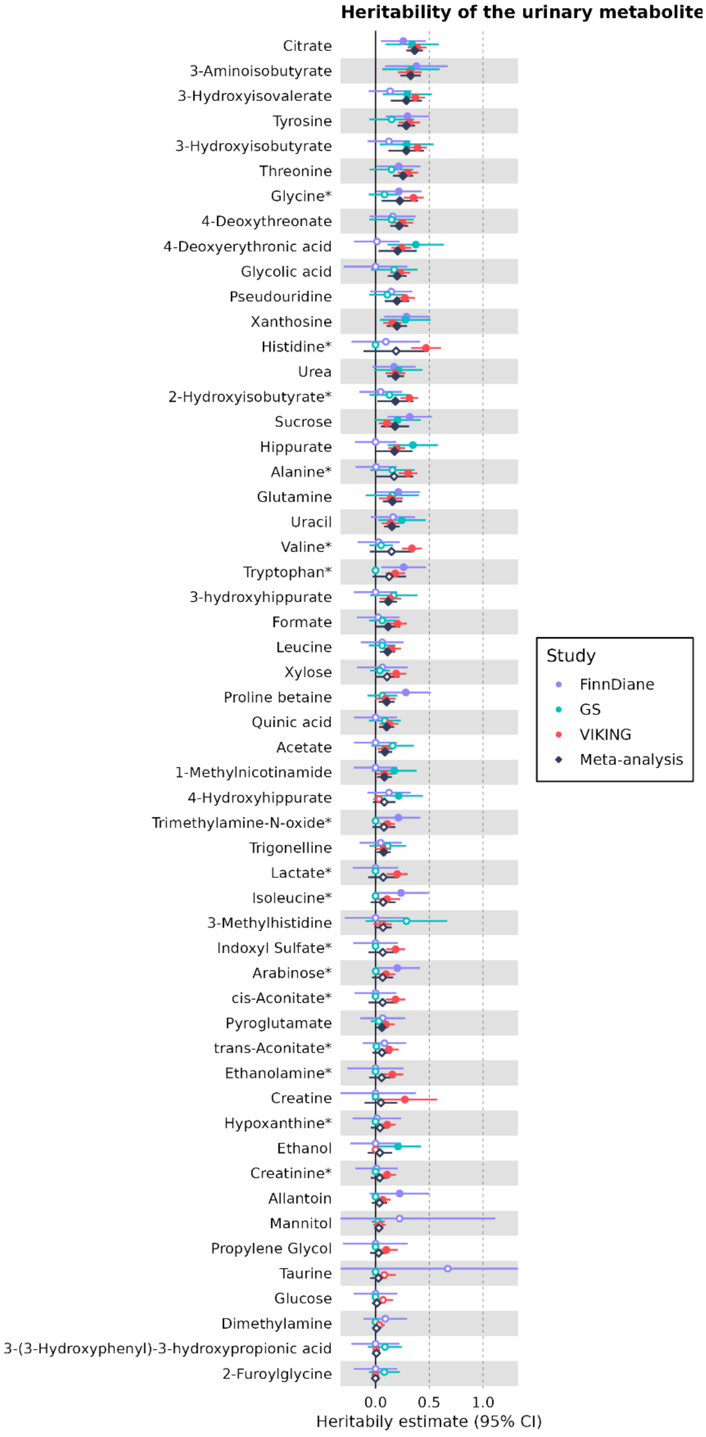

Supplementary Figure 2

LocusZoom plots of the COJO lead signals. Association with urinary metabolite 250kb around the COJO lead variant for each of the 54 genome-wide significant associations. Plot title includes the metabolite name and the lead variant rsid (chromosome:position:effect allele:non-effect allele). Two-sided p-values calculated with METAL applying inverse variance weighted method and genomic control correction for the individual study level results. r^2 is the squared correlation coefficient between a variant and the index variant. LD information was calculated using the 1000 Genomes Phase 3 European population.

3−Hydroxyisovalerate – rs114200864 (chr1:6,334,301:A:G)

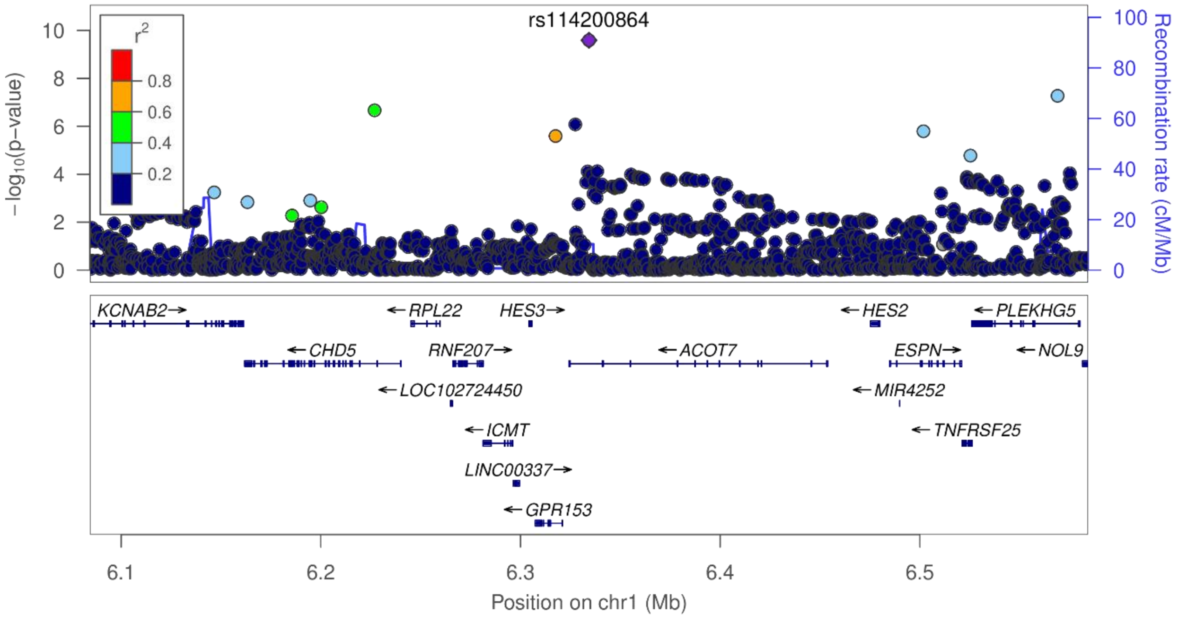

Xylose – rs10788884 (chr1:48,690,229:A:C)

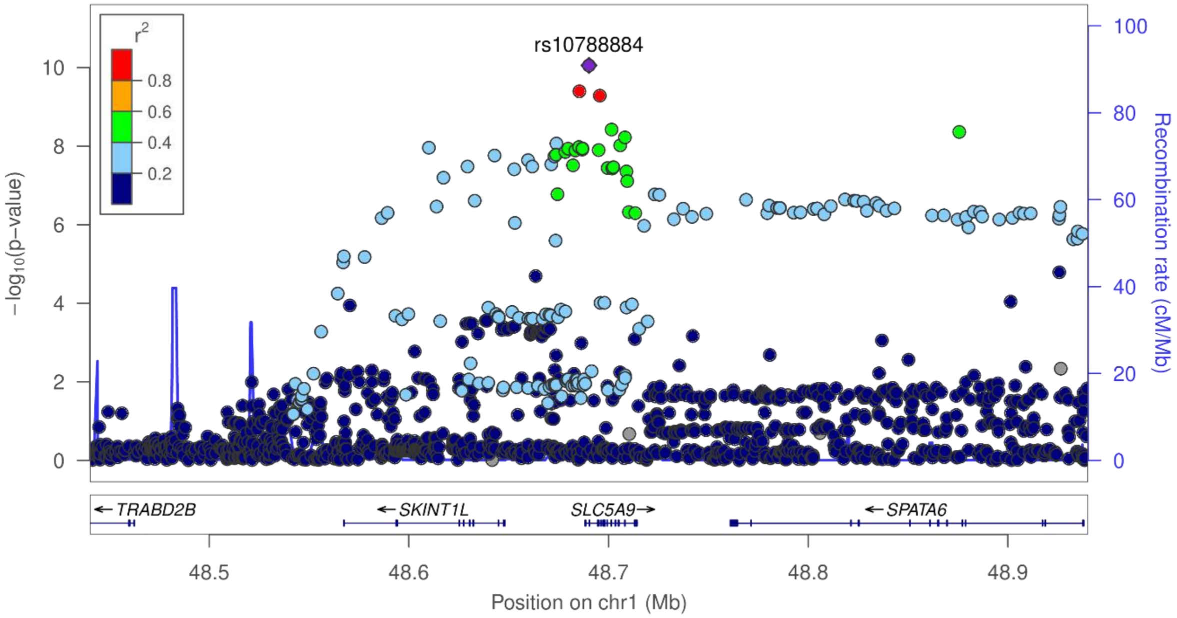

3−Hydroxyisovalerate – rs2999545 (chr1:151,904,146:A:T)

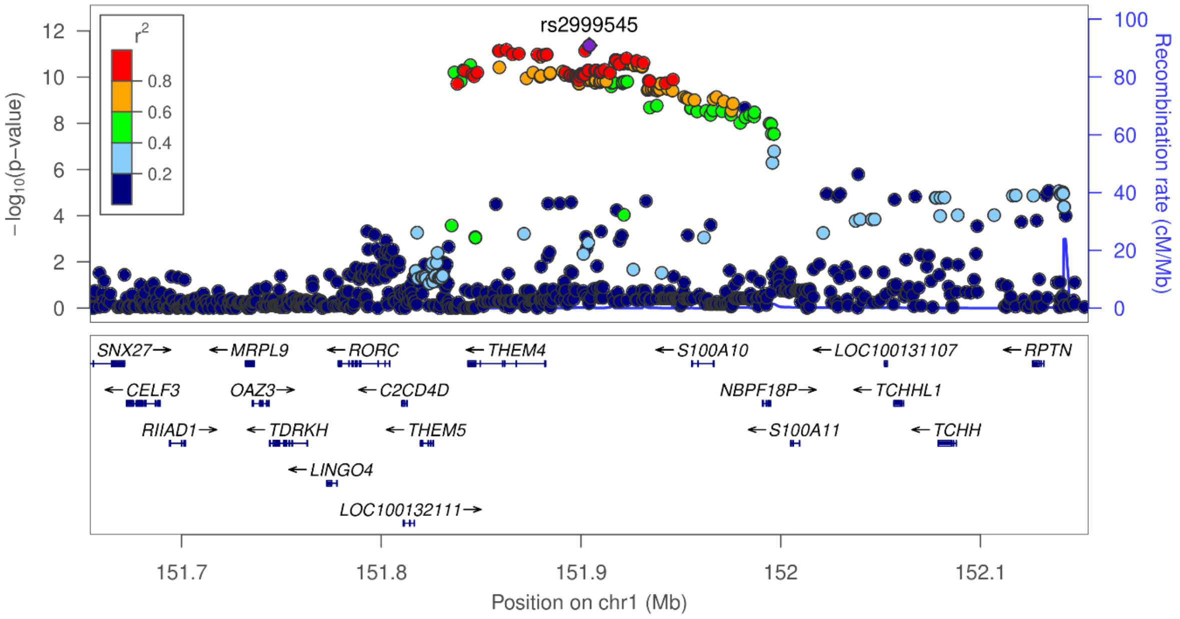

Formate – rs4846068 (chr1:11,940,483:T:C)

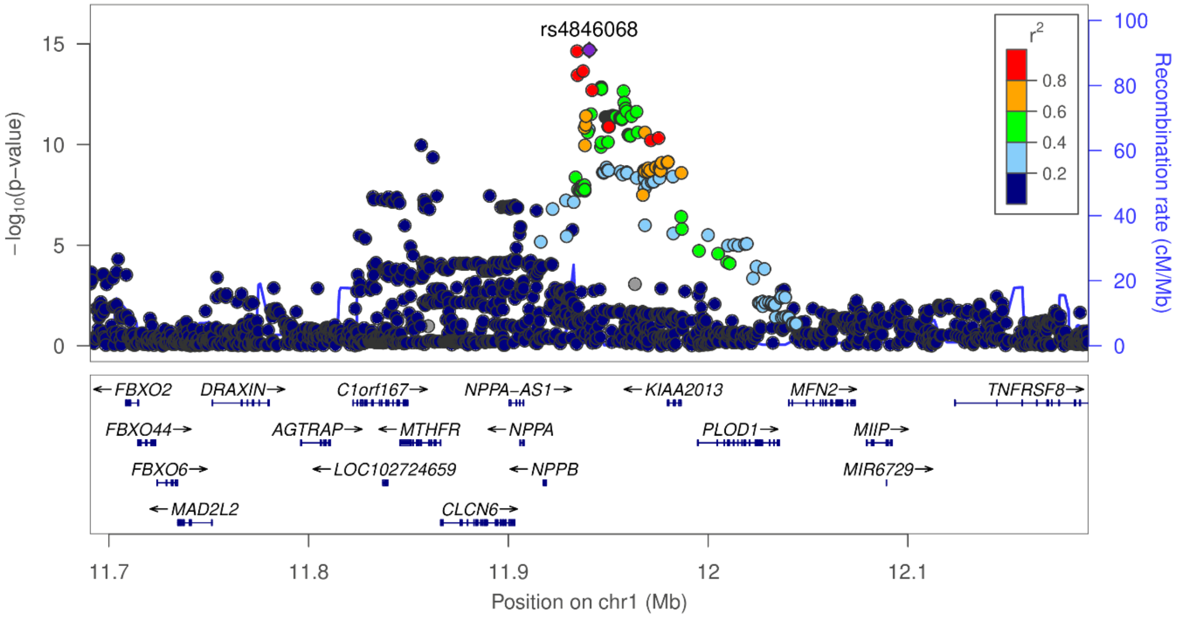

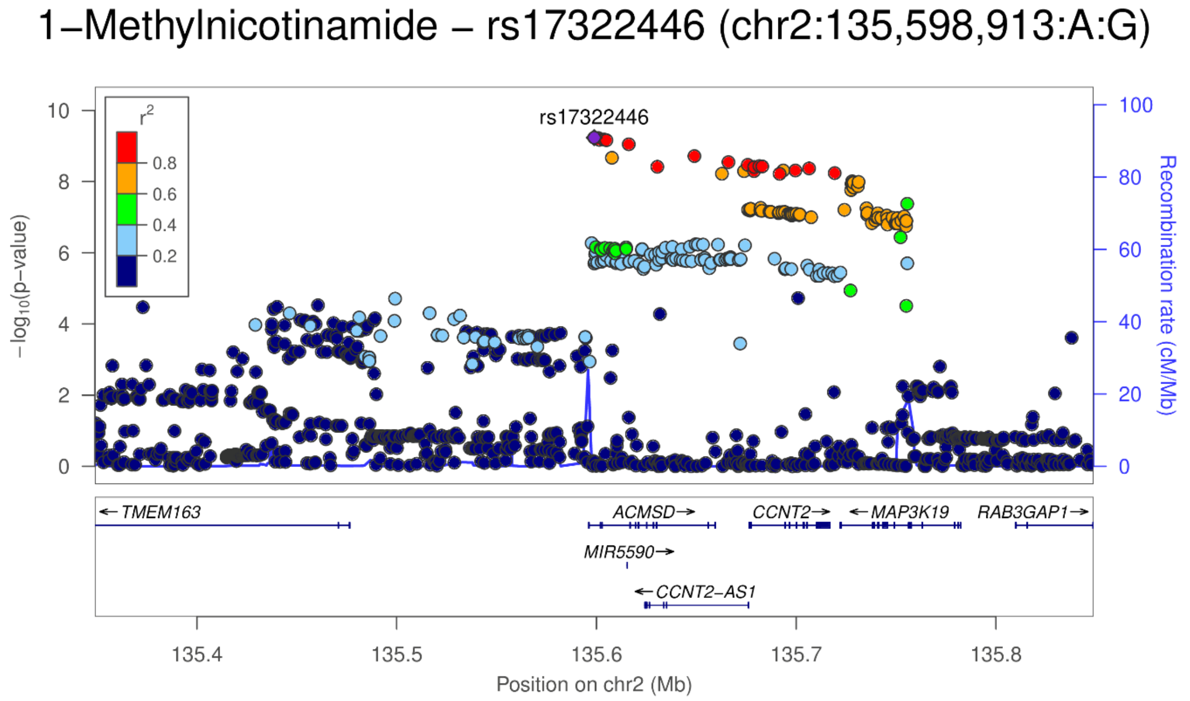
1−Methylnicotinamide − rs17322446 (chr2:135,598,913:A:G)
rs17322446
r2
0.8
0.6
0.4
0.2
−log10(p−value)
Recombination rate (cM/Mb)
TMEM163
ACMSD
CCNT2
MAP3K19
RAB3GAP1
MIR5590
CCNT2−AS1
Position on chr2 (Mb)

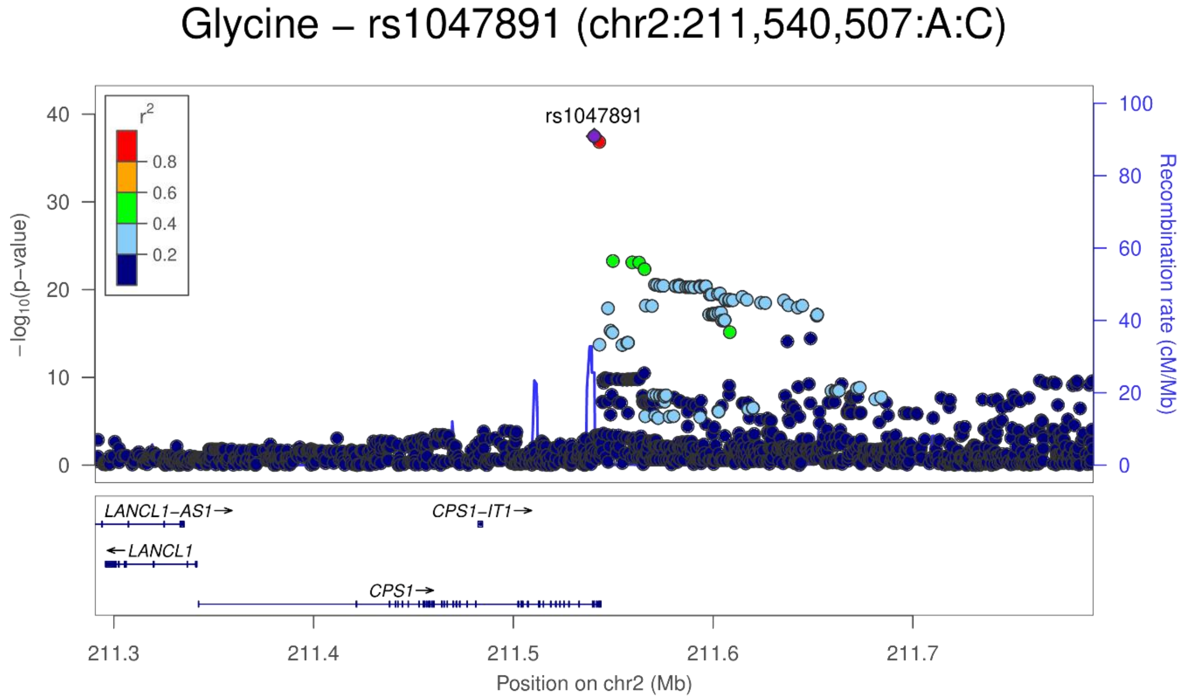
Glycine − rs1047891 (chr2:211,540,507:A:C)
rs1047891
r2
0.8
0.6
0.4
0.2
−log10(p−value)
Recombination rate (cM/Mb)
LANCL1−AS1
CPS1−IT1
LANCL1
CPS1
Position on chr2 (Mb)

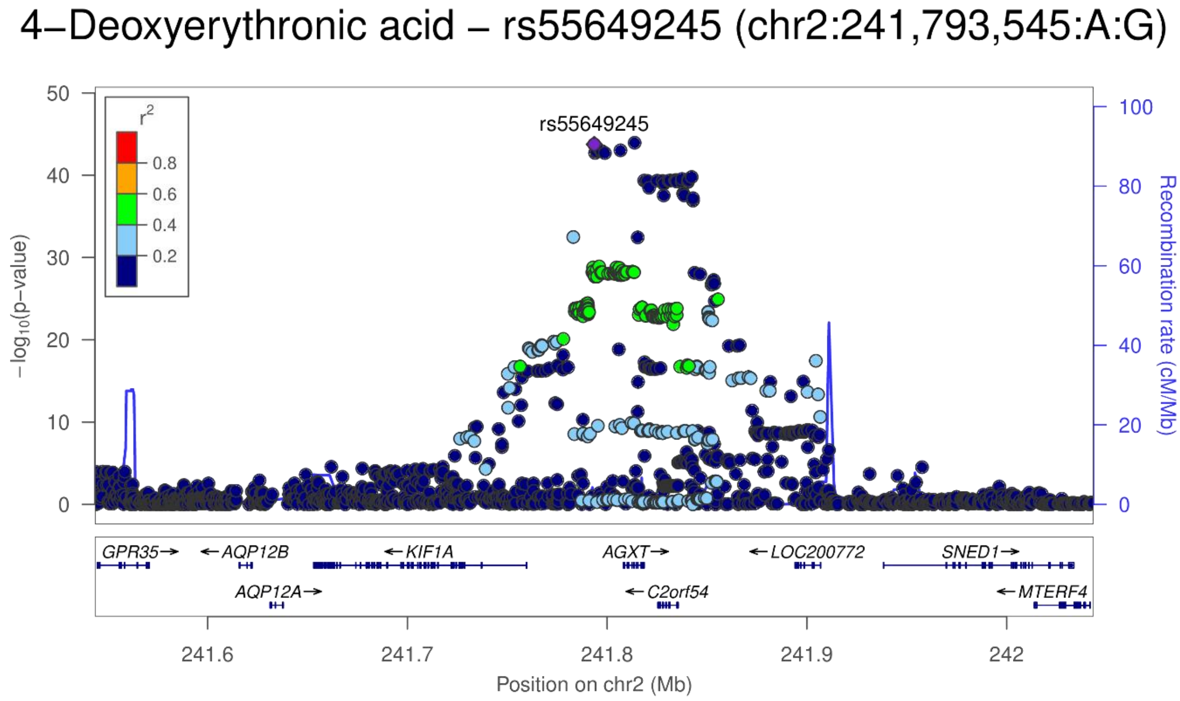
4−Deoxyerythronic acid − rs55649245 (chr2:241,793,545:A:G)
rs55649245
r2
0.8
0.6
0.4
0.2
−log10(p−value)
Recombination rate (cM/Mb)
GPR35
AQP12B
KIF1A
AGXT
LOC200772
SNED1
AQP12A
C2orf54
MTERF4
241.6
241.7
241.8
241.9
242
Position on chr2 (Mb)

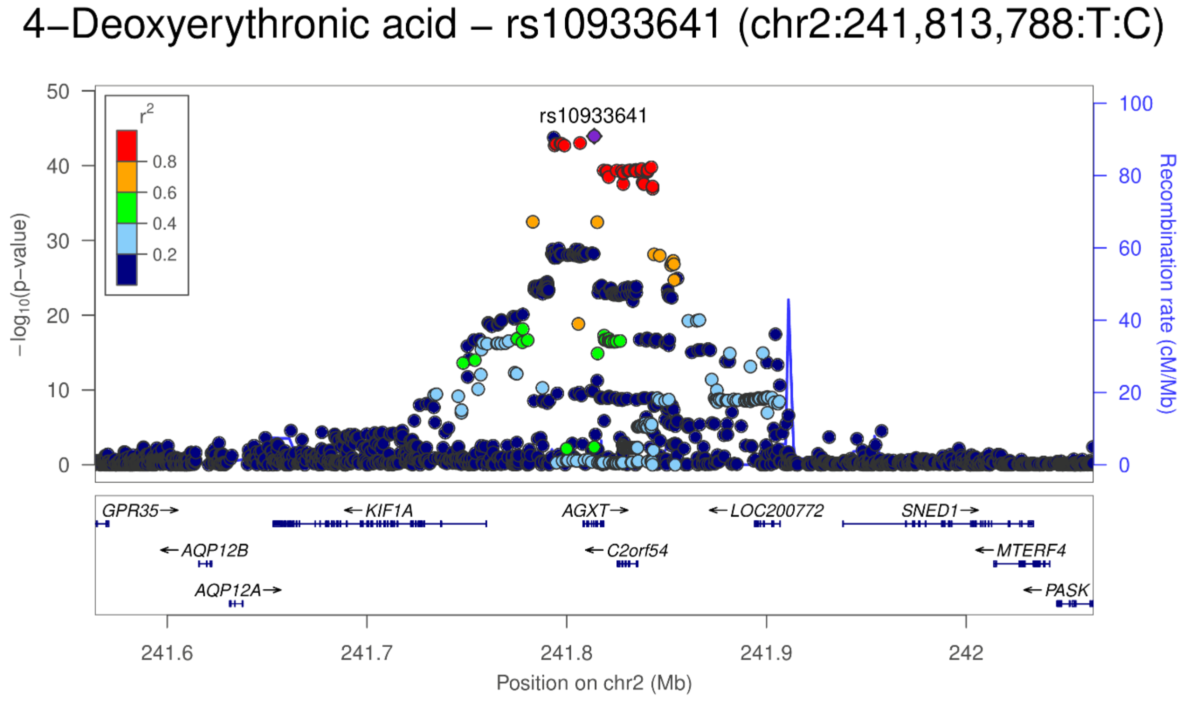
4−Deoxyerythronic acid − rs10933641 (chr2:241,813,788:T:C)
rs10933641
r2
0.8
0.6
0.4
0.2
−log10(p−value)
Recombination rate (cM/Mb)
GPR35
KIF1A
AGXT
LOC200772
SNED1
AQP12B
C2orf54
MTERF4
AQP12A
PASK
241.6
241.7
241.8
241.9
242
Position on chr2 (Mb)

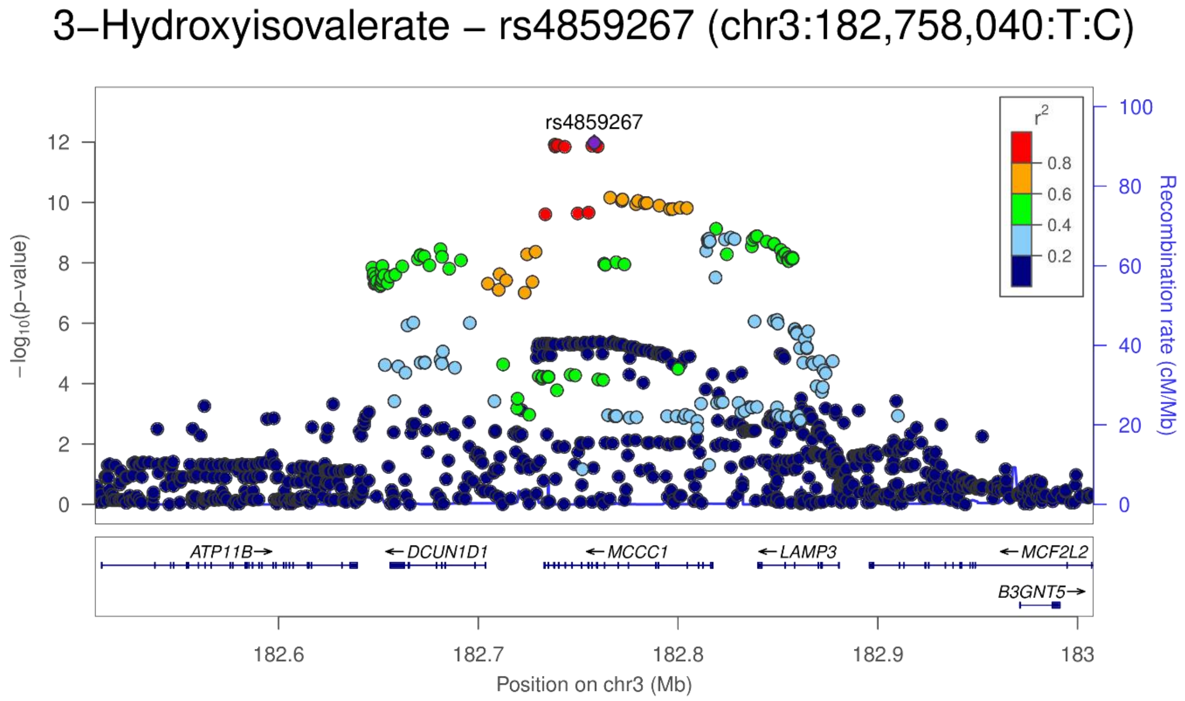
3−Hydroxyisovalerate − rs4859267 (chr3:182,758,040:T:C)
rs4859267
−log10(p−value)
Recombination rate (cM/Mb)
r2
ATP11B
DCUN1D1
MCCC1
LAMP3
MCF2L2
B3GNT5
Position on chr3 (Mb)

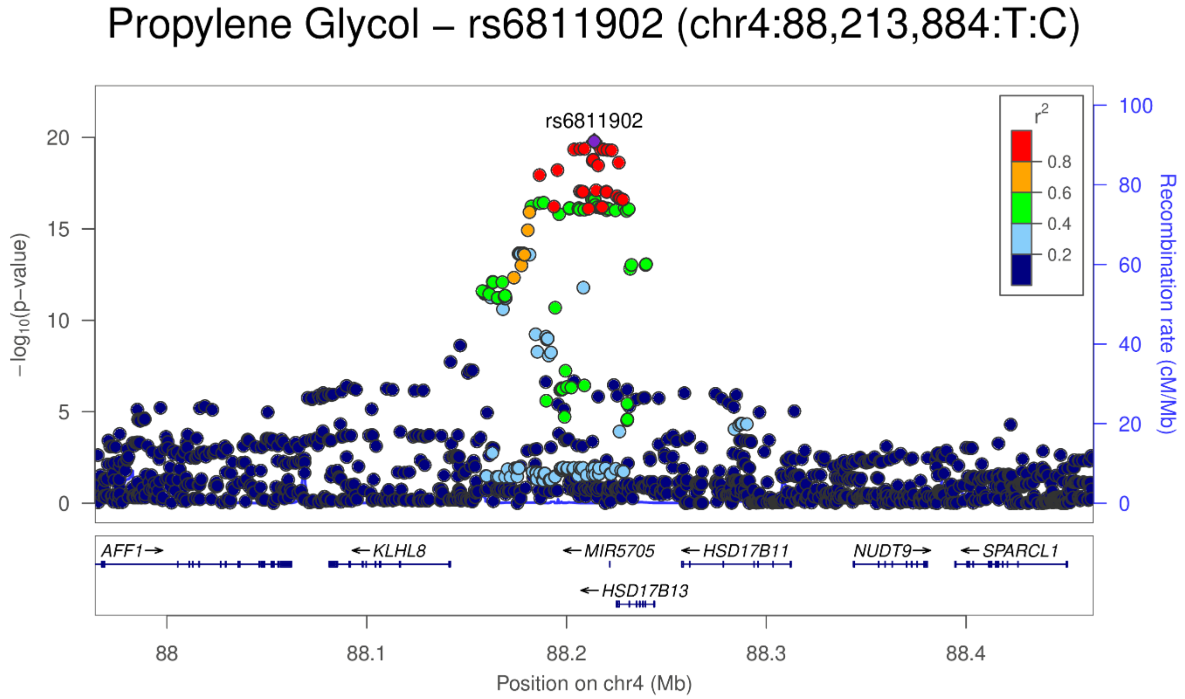
Propylene Glycol − rs6811902 (chr4:88,213,884:T:C)
rs6811902
−log10(p−value)
Recombination rate (cM/Mb)
r2
AFF1
KLHL8
MIR5705
HSD17B11
NUDT9
SPARCL1
HSD17B13
Position on chr4 (Mb)

Ethanolamine – rs62313082 (chr4:109,716,840:A:T)

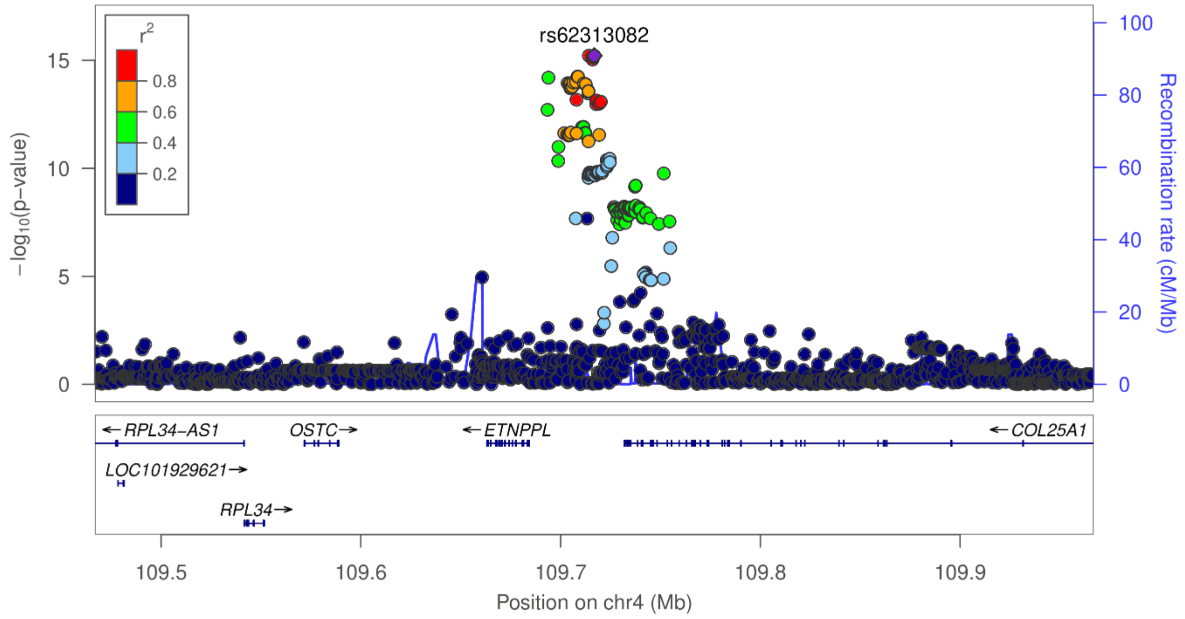

Glycine – rs11133665 (chr5:1,188,285:A:G)

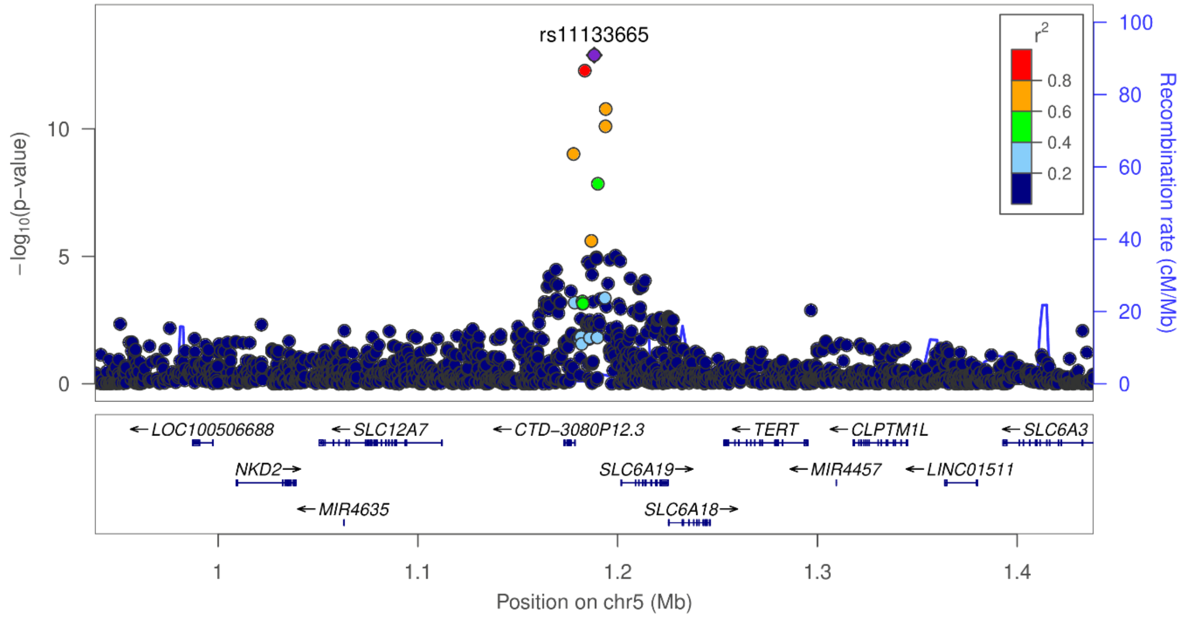

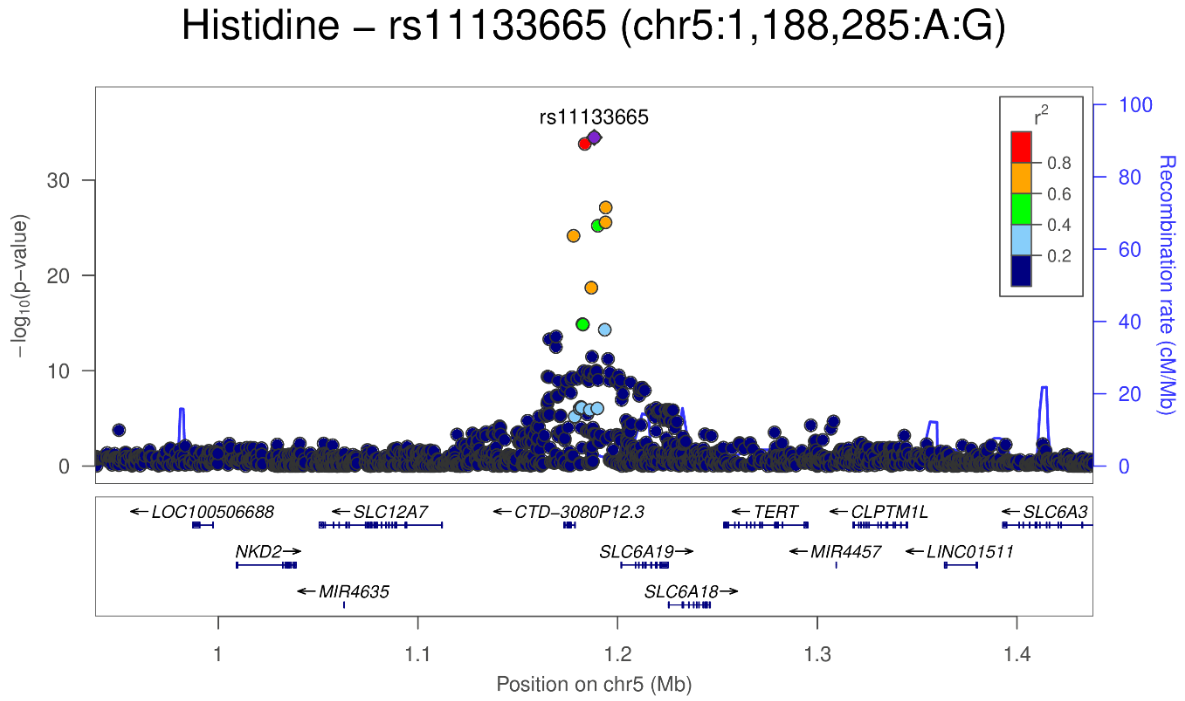
Histidine – rs11133665 (chr5:1,188,285:A:G)
rs11133665
r2
0.8
0.6
0.4
0.2
−log10(p−value)
Recombination rate (cM/Mb)
LOC100506688
SLC12A7
CTD−3080P12.3
TERT
CLPTM1L
SLC6A3
NKD2
SLC6A19
MIR4457
LINC01511
MIR4635
SLC6A18
Position on chr5 (Mb)

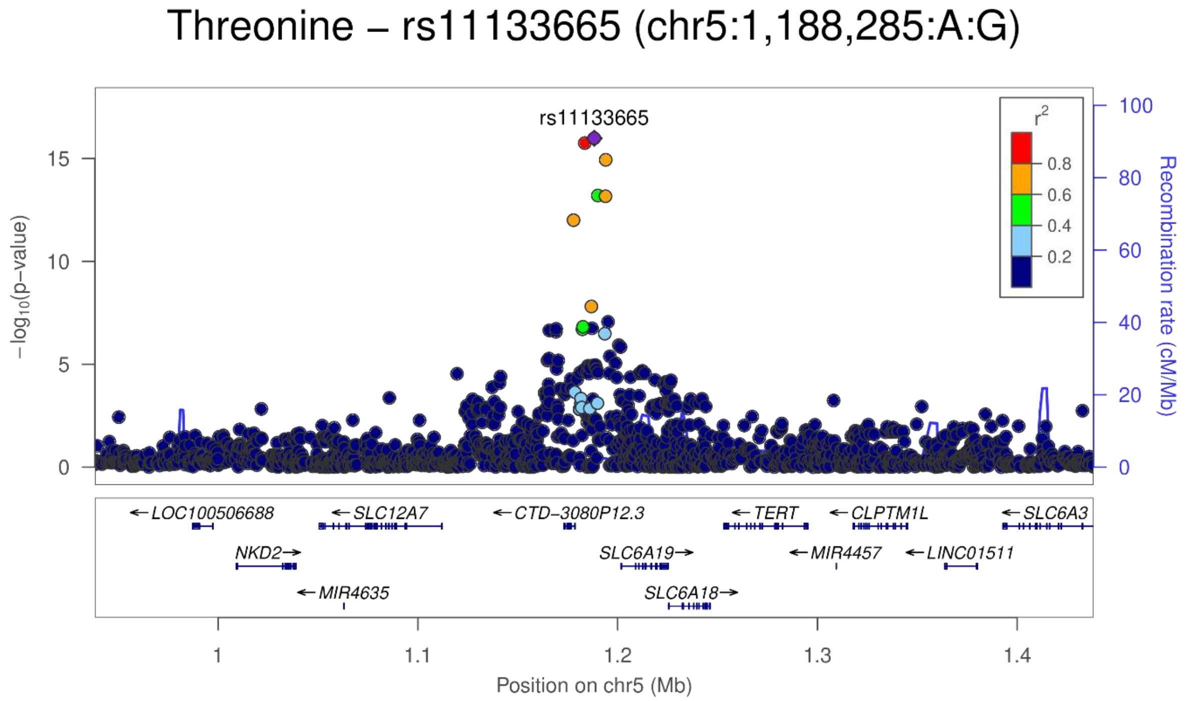
Threonine – rs11133665 (chr5:1,188,285:A:G)
rs11133665
r2
0.8
0.6
0.4
0.2
−log10(p−value)
Recombination rate (cM/Mb)
LOC100506688
SLC12A7
CTD−3080P12.3
TERT
CLPTM1L
SLC6A3
NKD2
SLC6A19
MIR4457
LINC01511
MIR4635
SLC6A18
Position on chr5 (Mb)

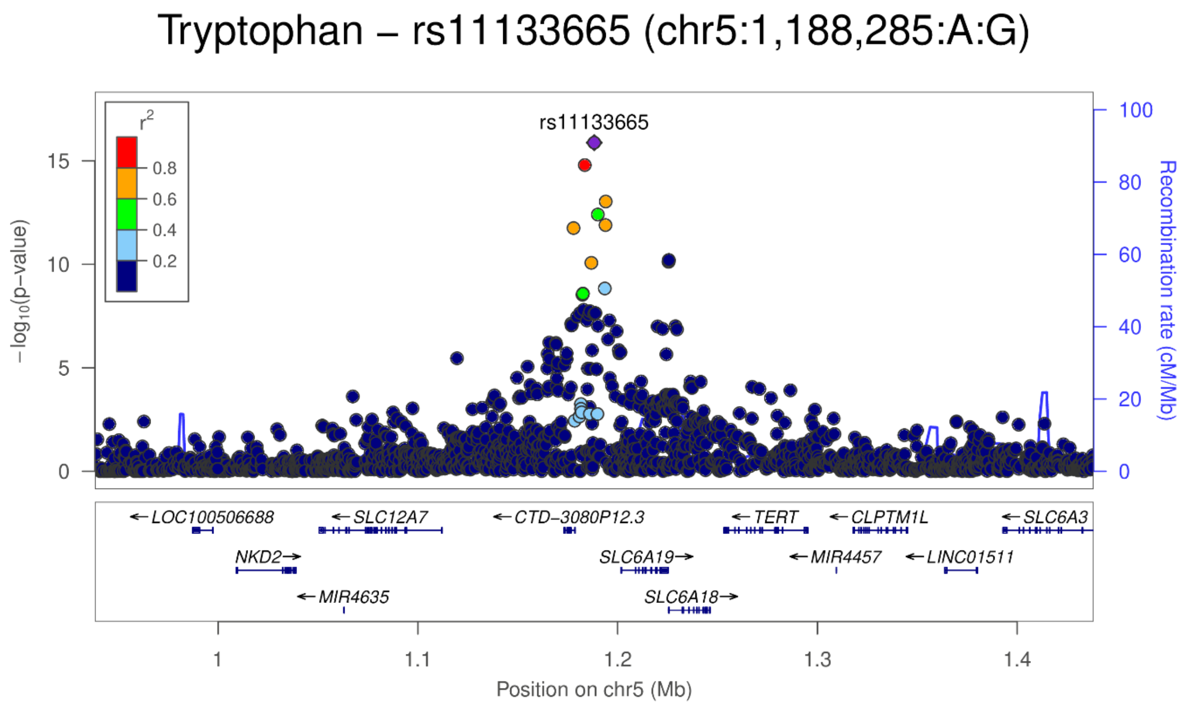

Tryptophan – rs11133665 (chr5:1,188,285:A:G)
rs11133665
r2
0.8
0.6
0.4
0.2
−log10(p−value)
Recombination rate (cM/Mb)
LOC100506688
SLC12A7
CTD−3080P12.3
TERT
CLPTM1L
SLC6A3
NKD2
SLC6A19
MIR4457
LINC01511
MIR4635
SLC6A18
Position on chr5 (Mb)

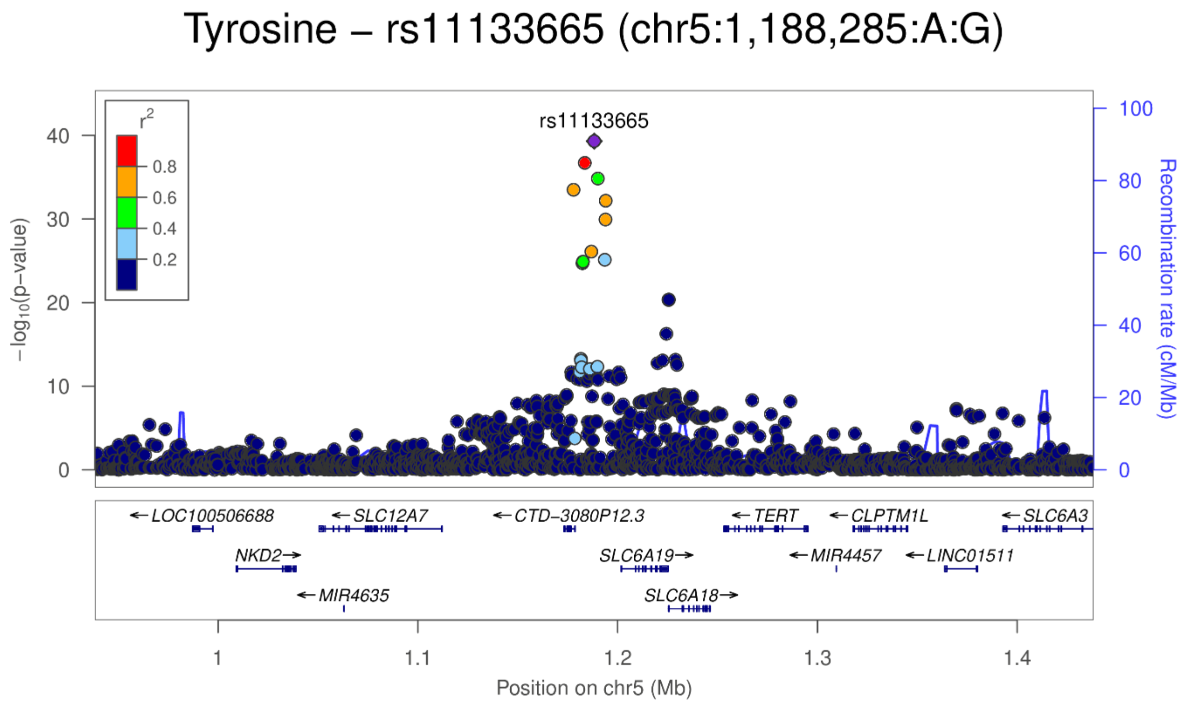

Tyrosine – rs11133665 (chr5:1,188,285:A:G)
rs11133665
r2
0.8
0.6
0.4
0.2
−log10(p−value)
Recombination rate (cM/Mb)
LOC100506688
SLC12A7
CTD−3080P12.3
TERT
CLPTM1L
SLC6A3
NKD2
SLC6A19
MIR4457
LINC01511
MIR4635
SLC6A18
Position on chr5 (Mb)

Tyrosine – rs7704882 (chr5:1,225,434:T:C)

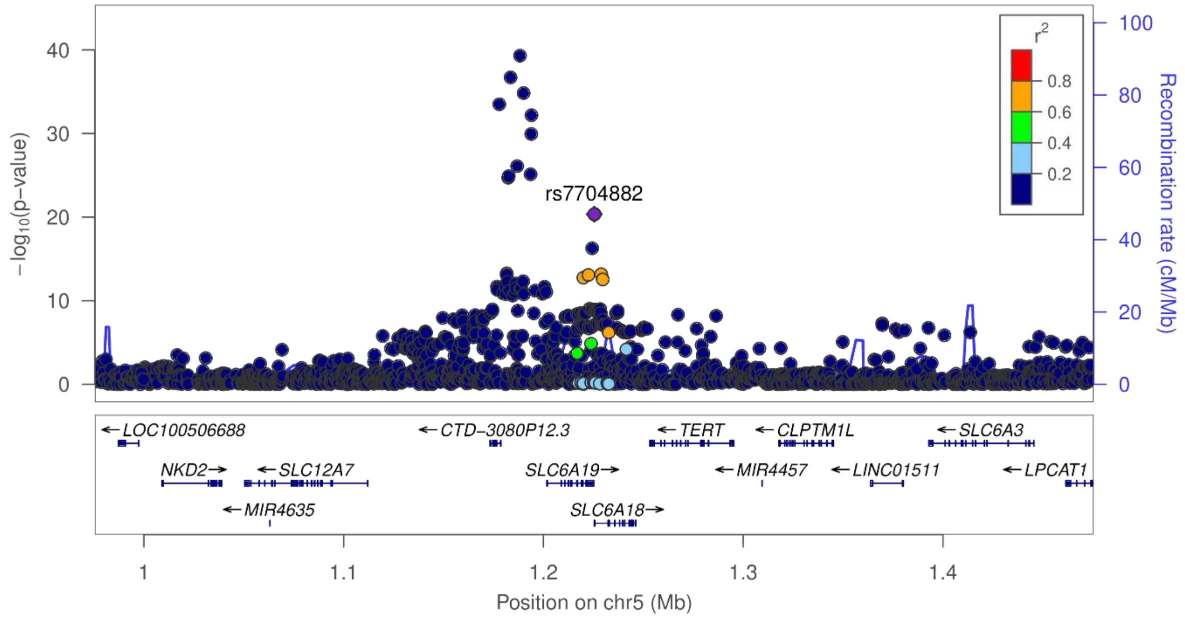

Tryptophan – rs7704058 (chr5:1,225,613:A:G)

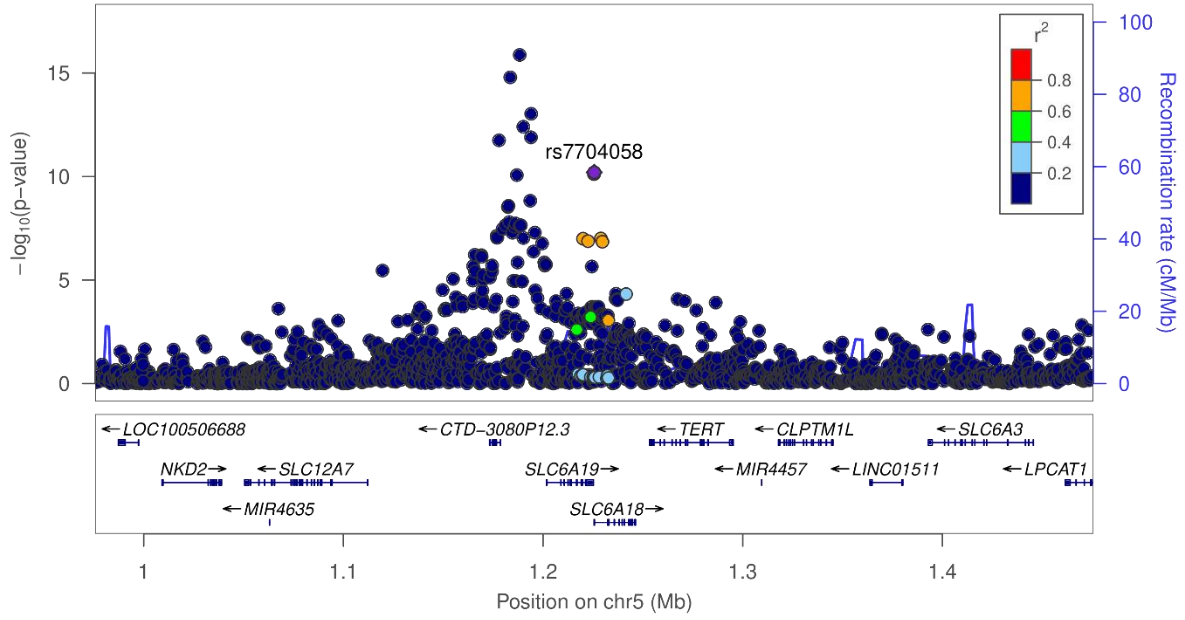

3−Aminoisobutyrate − rs9292560 (chr5:34,742,391:C:G)

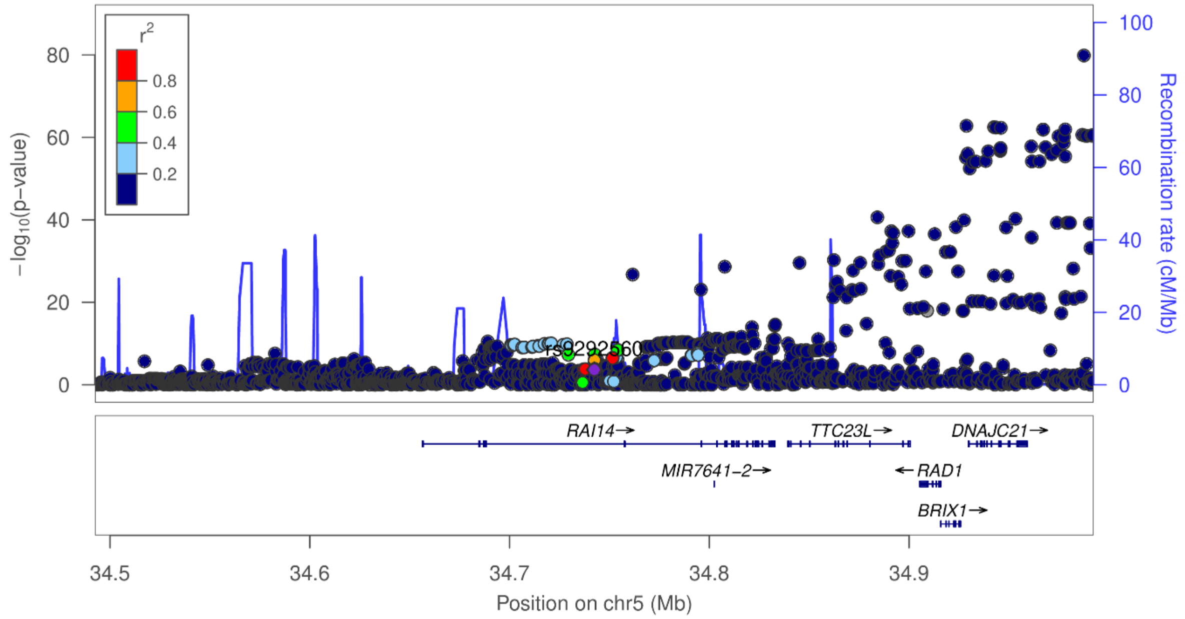

3−Aminoisobutyrate − rs336494 (chr5:34,849,791:A:T)

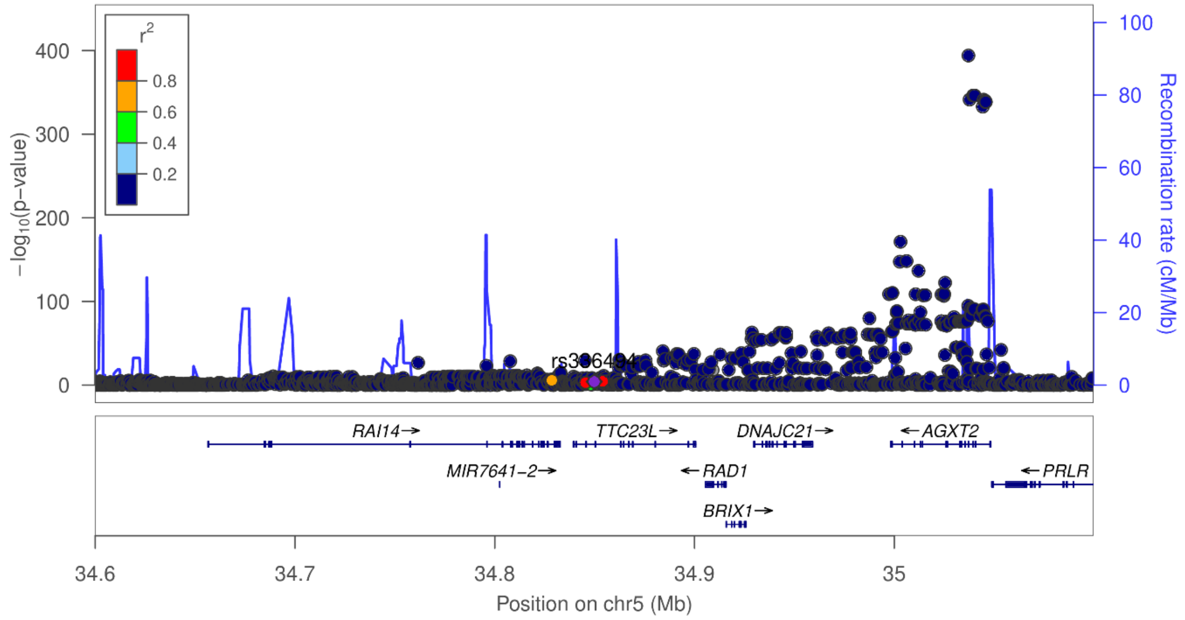

3−Aminoisobutyrate – rs72732827 (chr5:34,868,497:A:T)

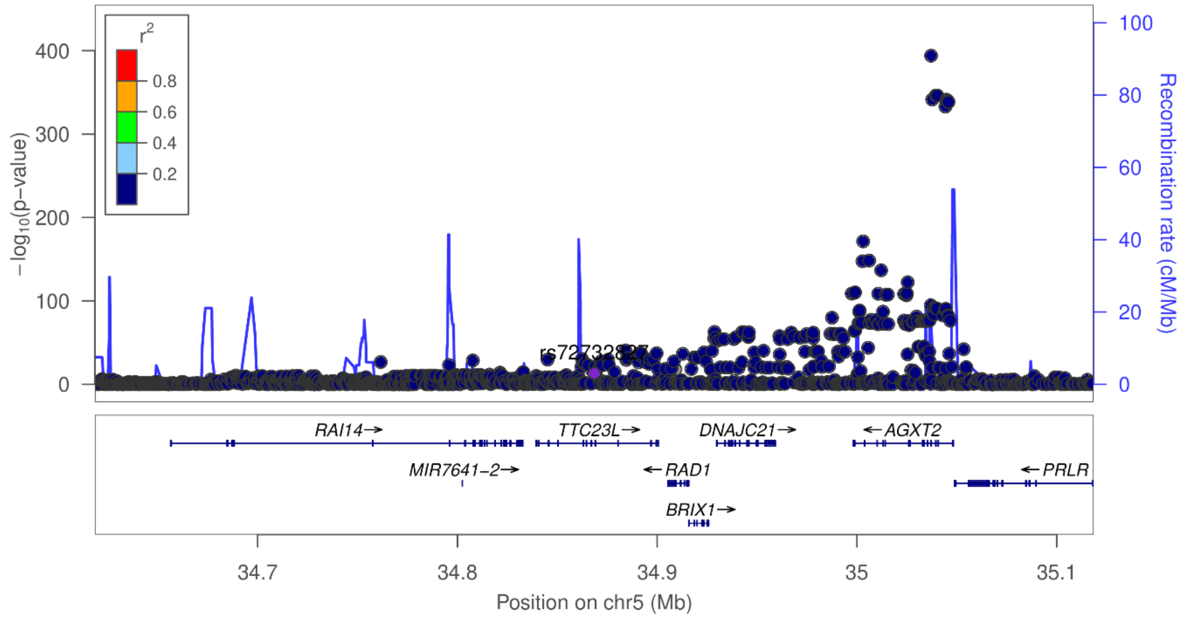

3−Aminoisobutyrate – rs138425947 (chr5:34,896,132:A:G)

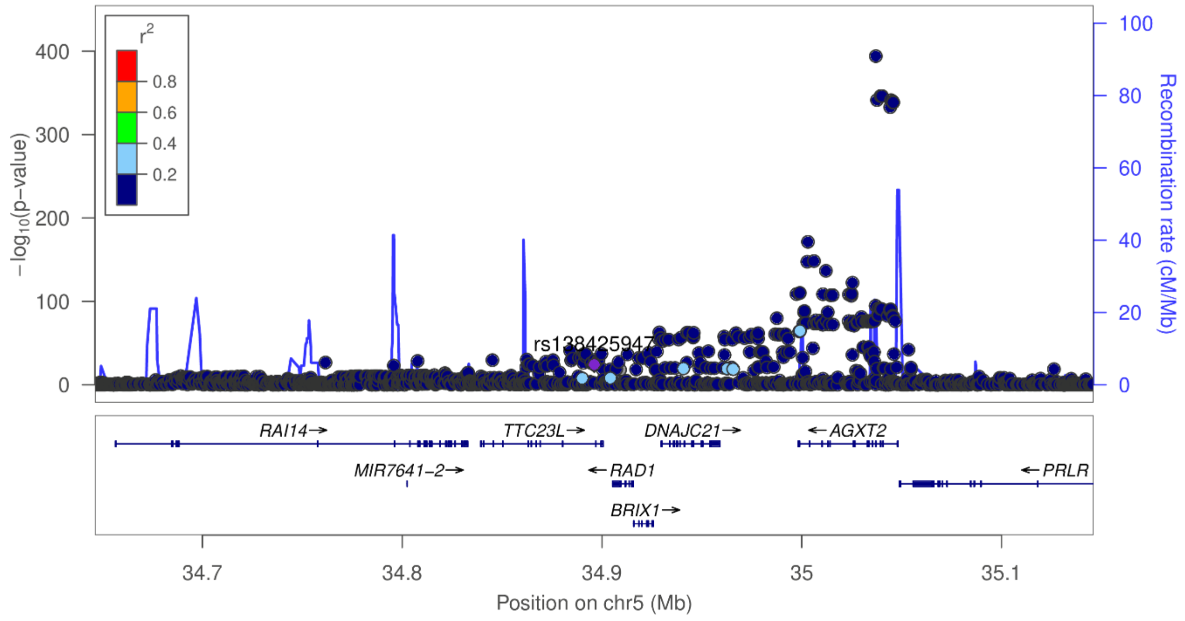

3−Aminoisobutyrate − rs56007938 (chr5:34,899,723:T:C)

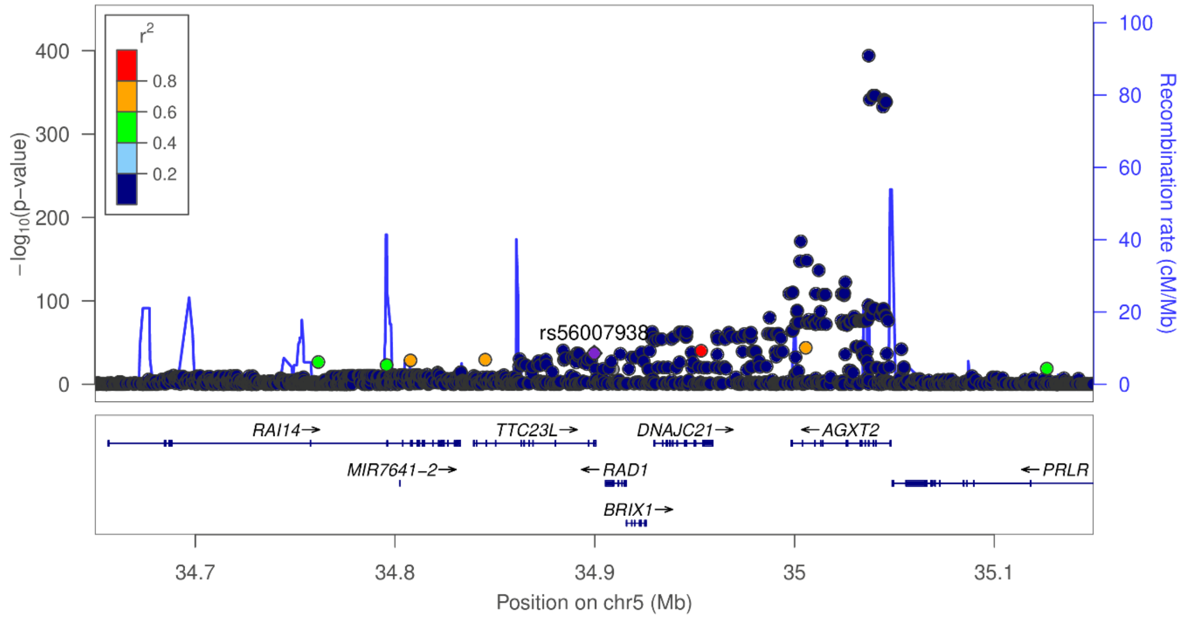

3−Aminoisobutyrate − rs2308957 (chr5:34,911,884:T:C)

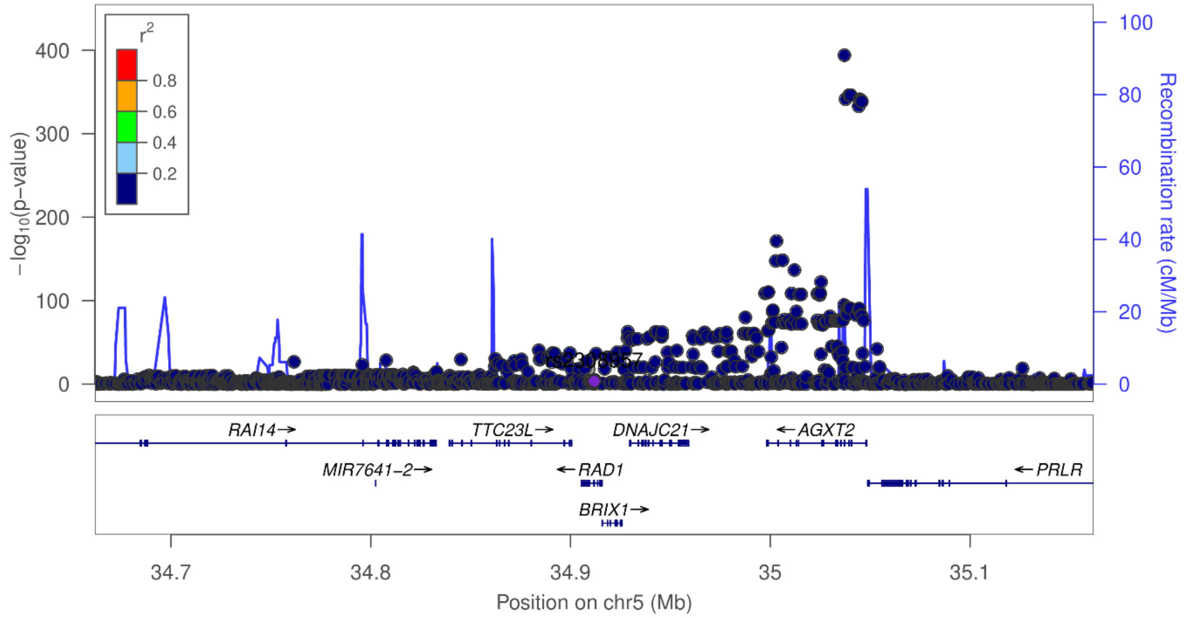

3−Aminoisobutyrate − rs116116288 (chr5:34,982,167:T:C)

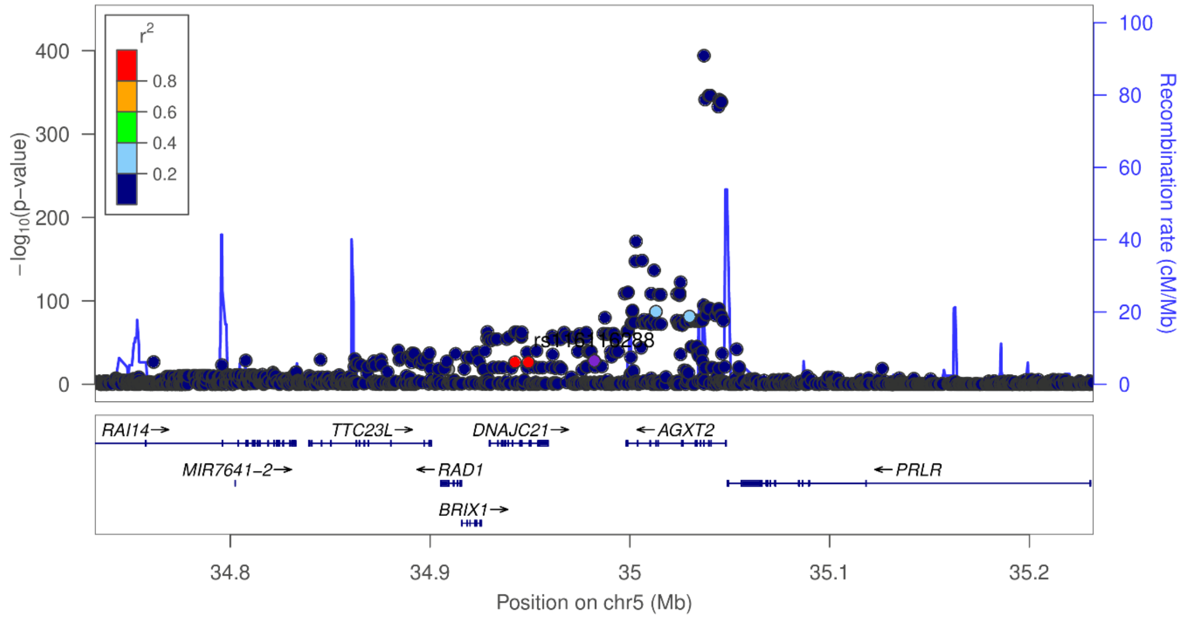

3−Aminoisobutyrate − rs11744796 (chr5:34,993,215:A:G)

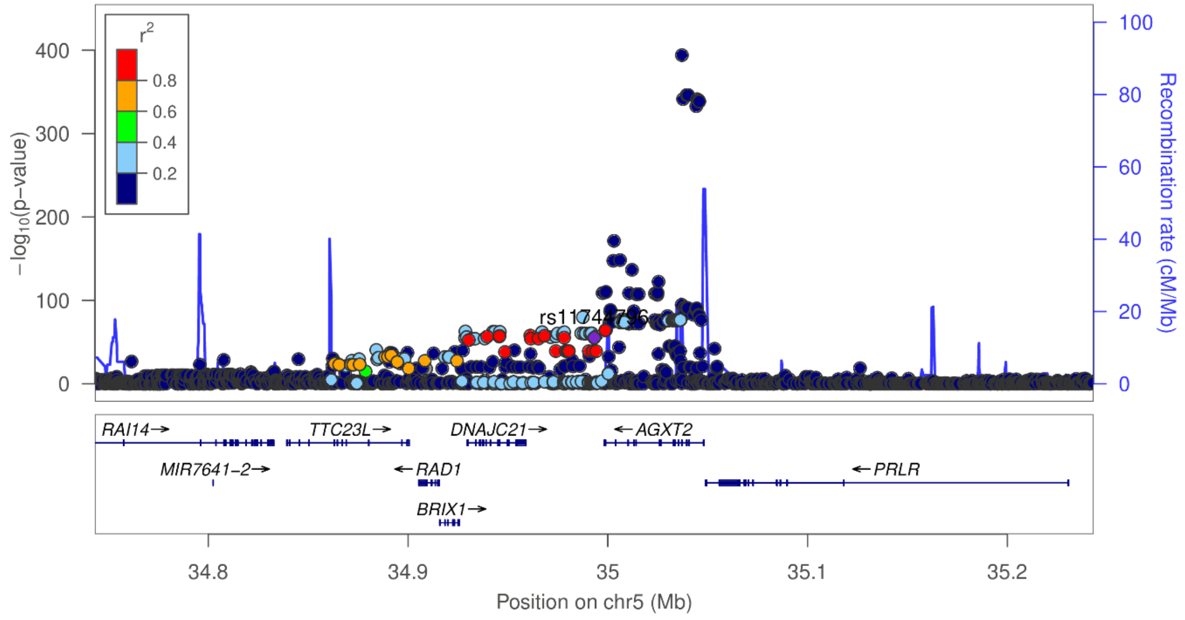

3−Aminoisobutyrate − rs7737763 (chr5:35,000,653:T:C)

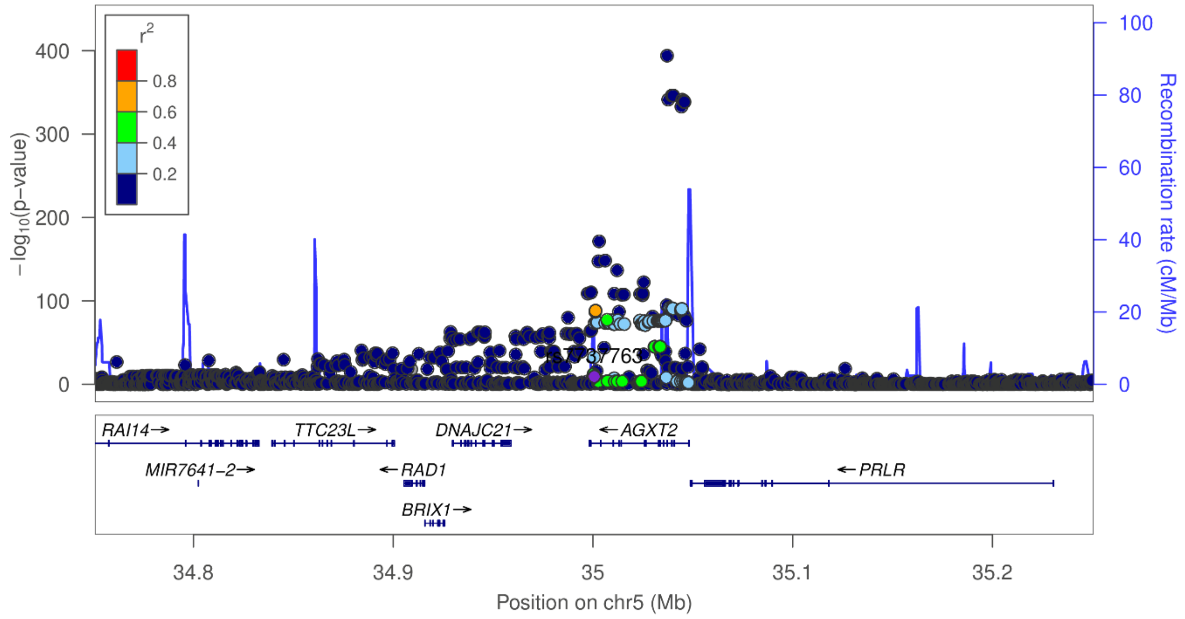

3−Aminoisobutyrate − rs468327 (chr5:35,003,112:A:G)

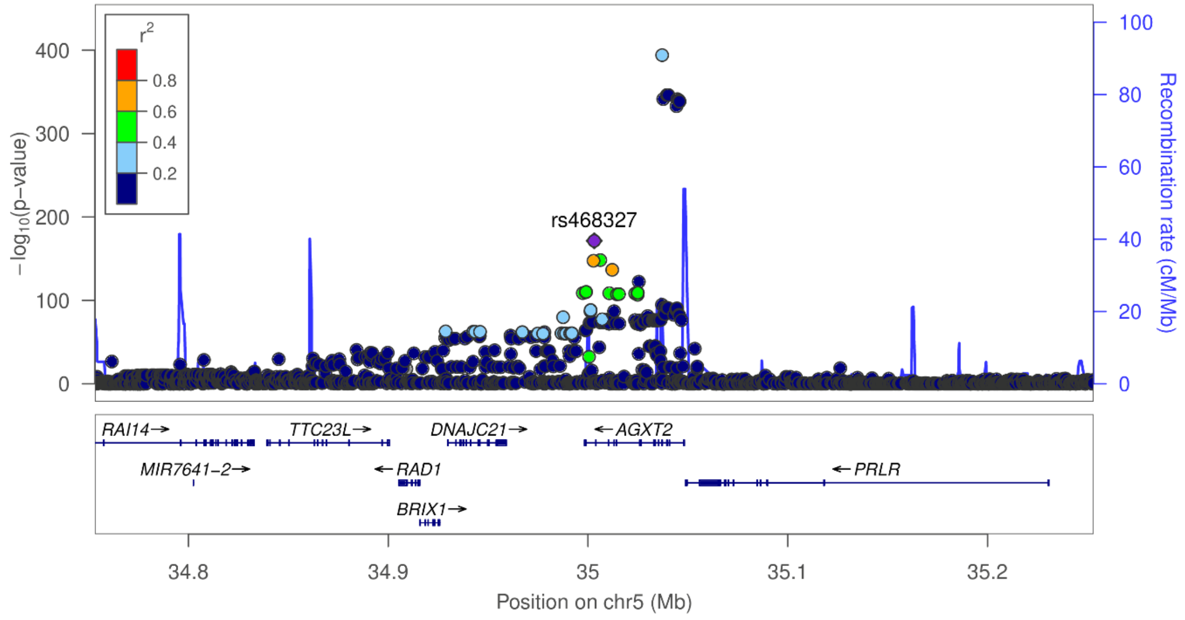

3−Aminoisobutyrate − rs37369 (chr5:35,037,115:T:C)

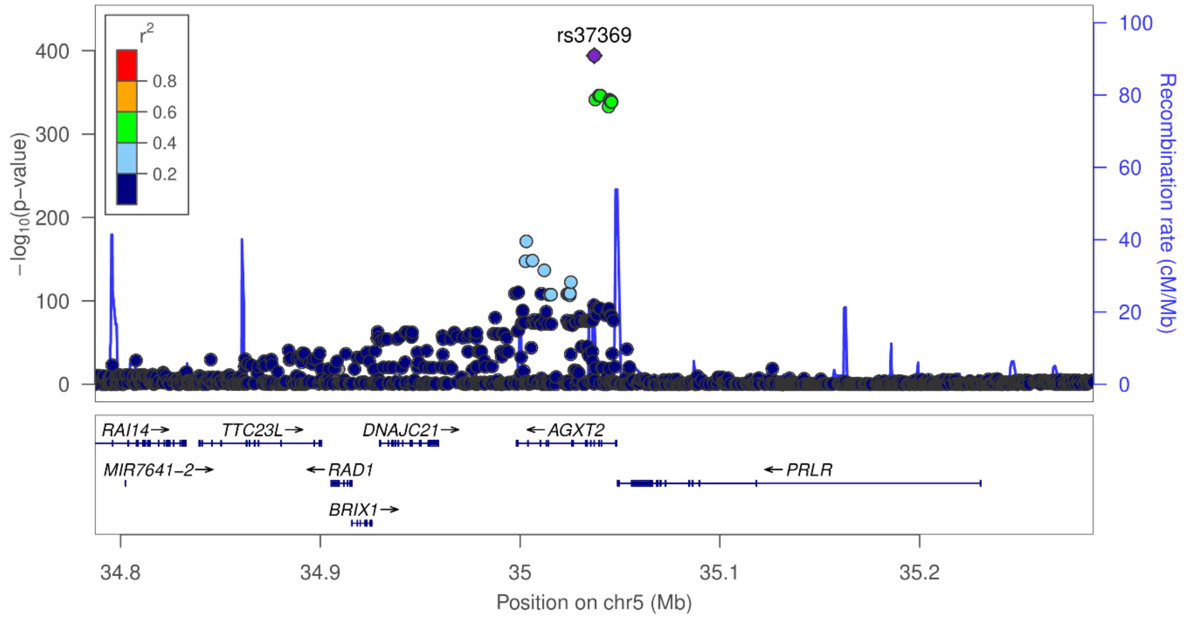

3−Aminoisobutyrate − rs2279651 (chr5:35,039,437:A:G)

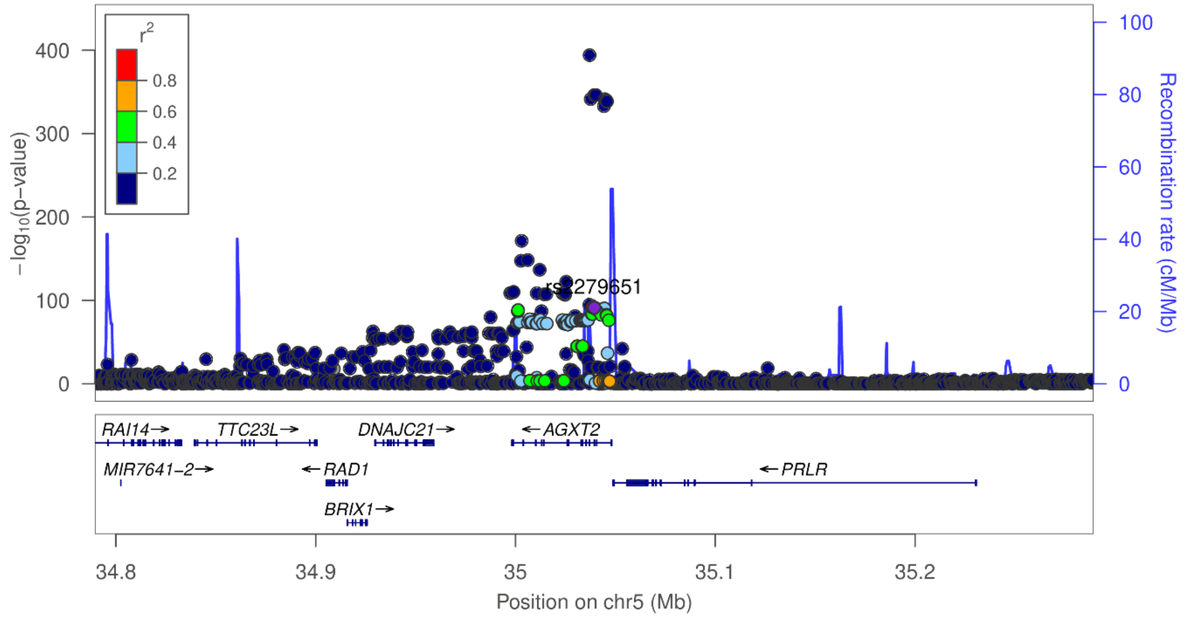

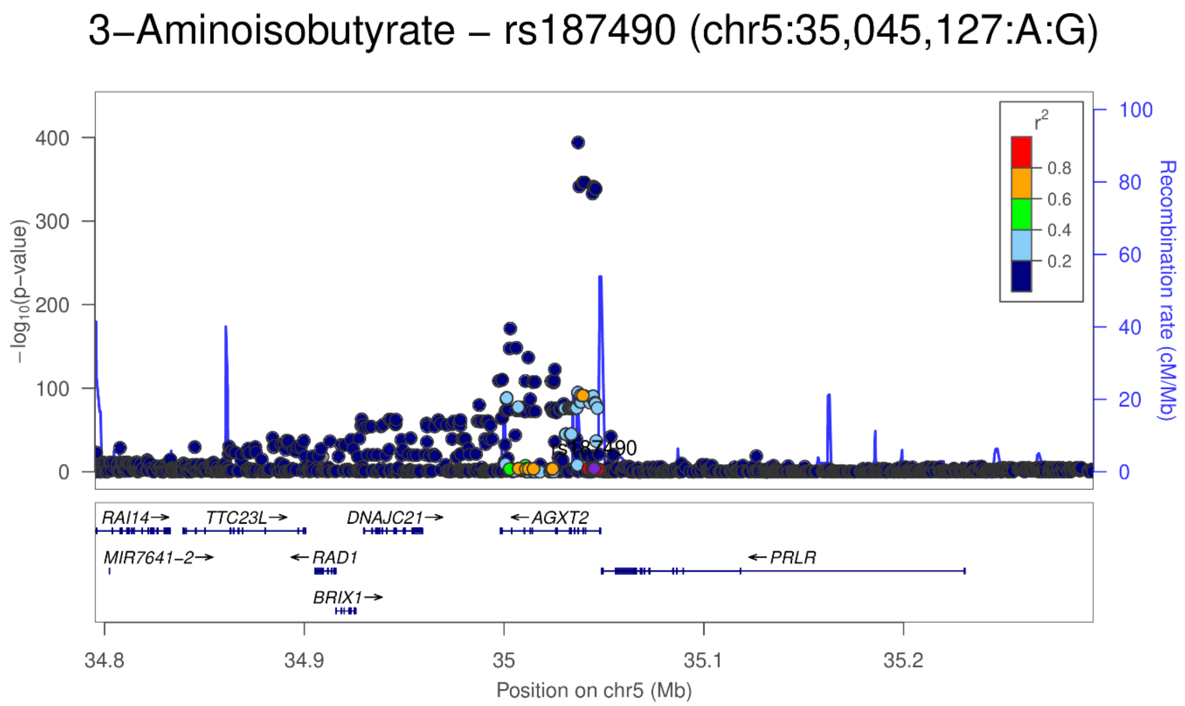

3−Aminoisobutyrate − rs187490 (chr5:35,045,127:A:G)
−log10(p−value)
Recombination rate (cM/Mb)
r2
0.8
0.6
0.4
0.2
rs187490
RAI14
TTC23L
DNAJC21
AGXT2
MIR7641−2
RAD1
PRLR
BRIX1
34.8
34.9
35
35.1
35.2
Position on chr5 (Mb)

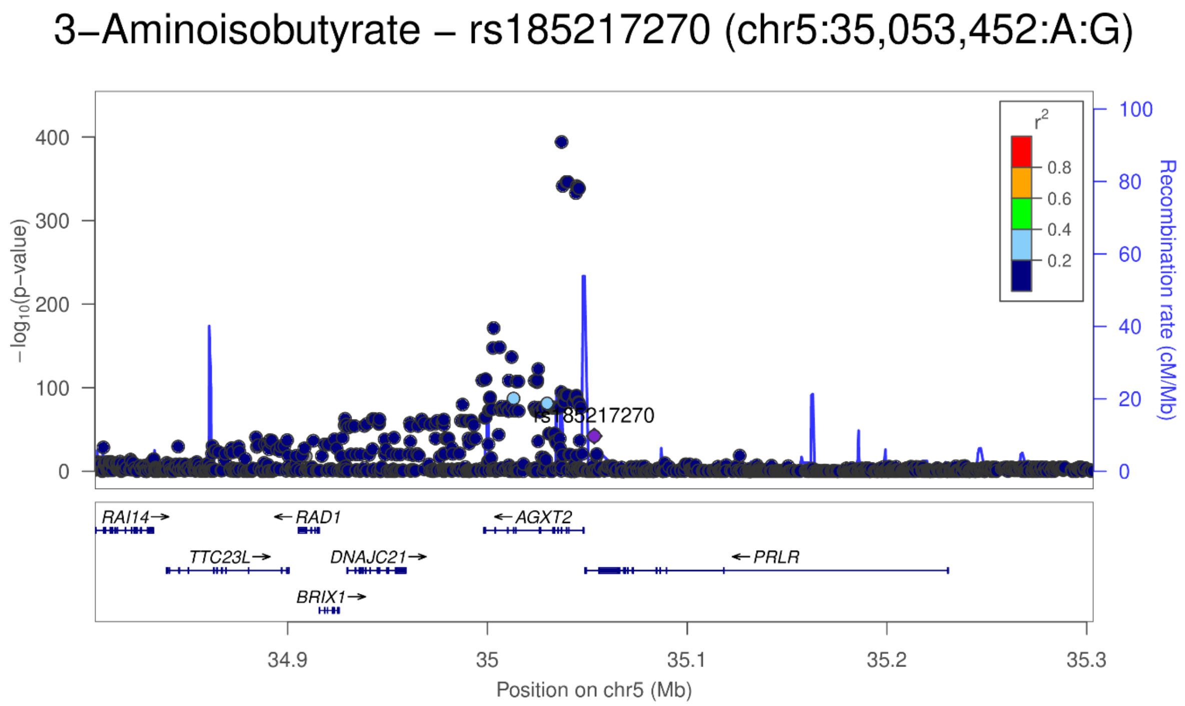

3−Aminoisobutyrate − rs185217270 (chr5:35,053,452:A:G)
−log10(p−value)
Recombination rate (cM/Mb)
r2
0.8
0.6
0.4
0.2
rs185217270
RAI14
RAD1
AGXT2
TTC23L
DNAJC21
PRLR
BRIX1
34.9
35
35.1
35.2
35.3
Position on chr5 (Mb)

3−Aminoisobutyrate – rs138373837 (chr5:36,219,710:T:C)

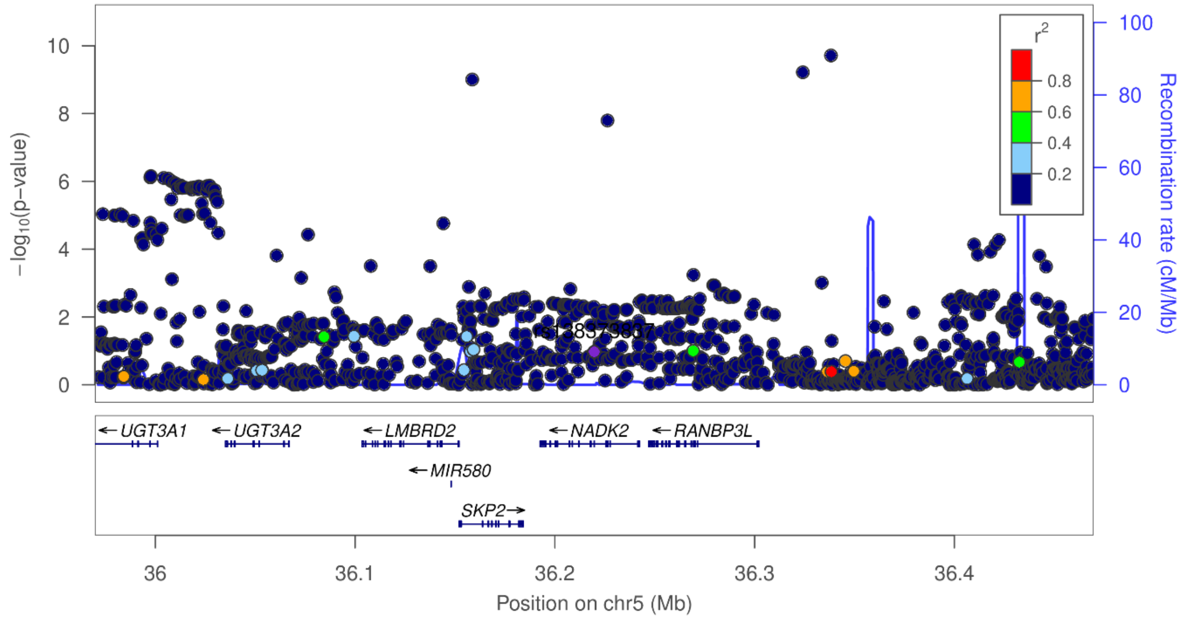

Glycine – rs72794144 (chr5:150,624,099:T:C)

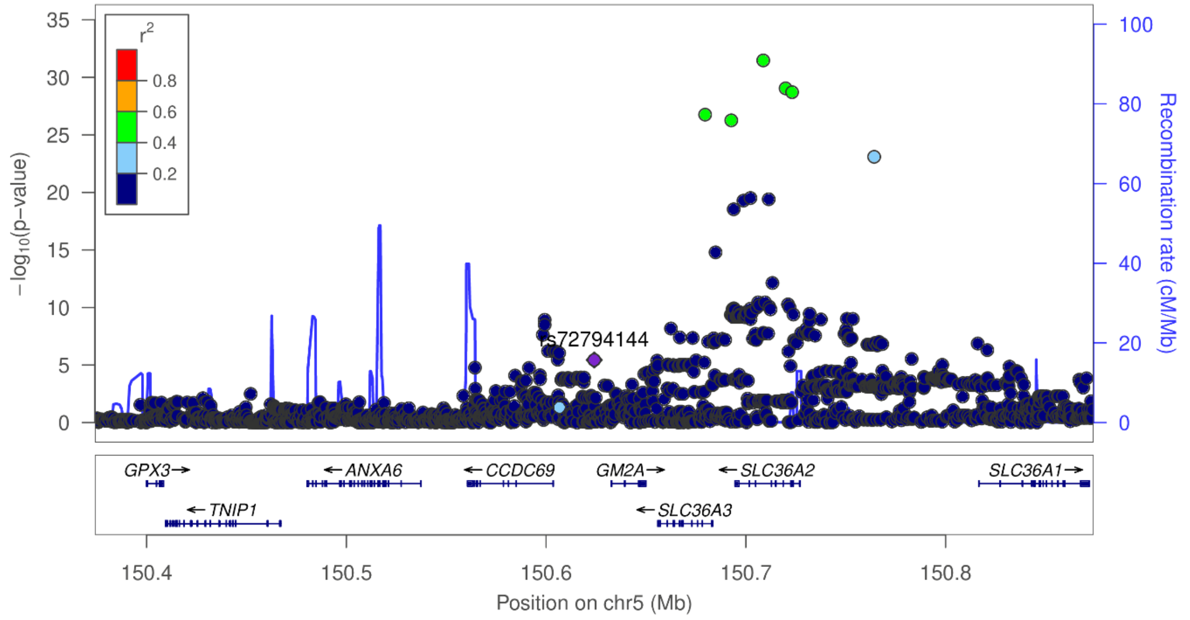

Glycine – rs61067578 (chr5:150,702,299:A:G)

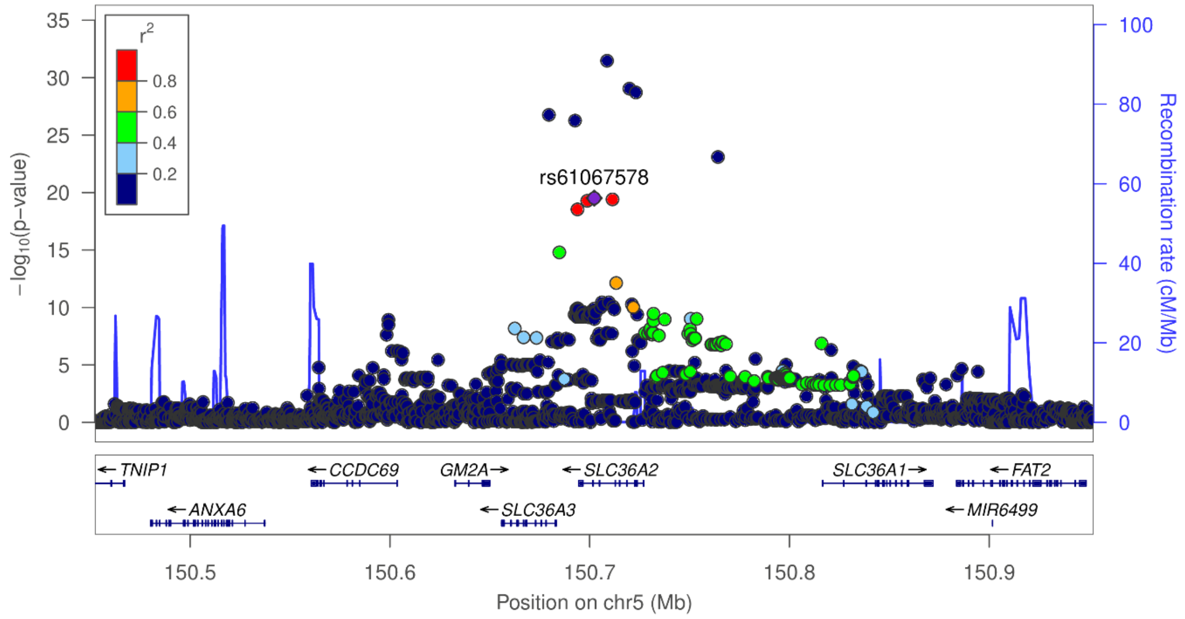

Glycine – rs147000073 (chr5:150,708,711:C:G)

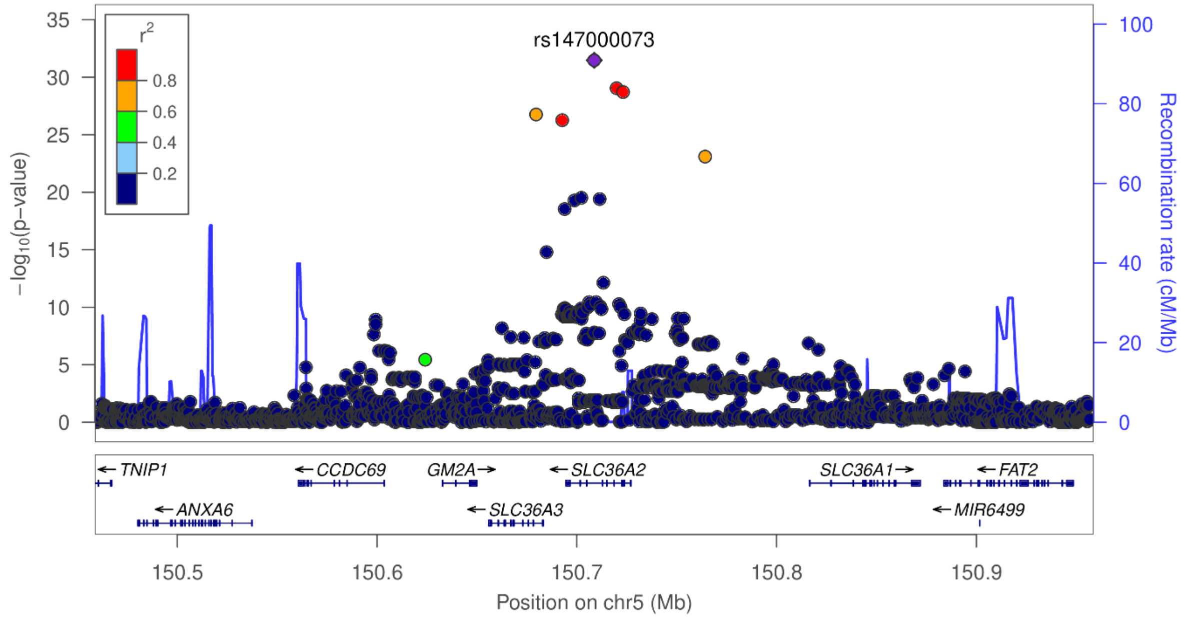

Tyrosine – rs241768 (chr6:111,492,119:T:C)

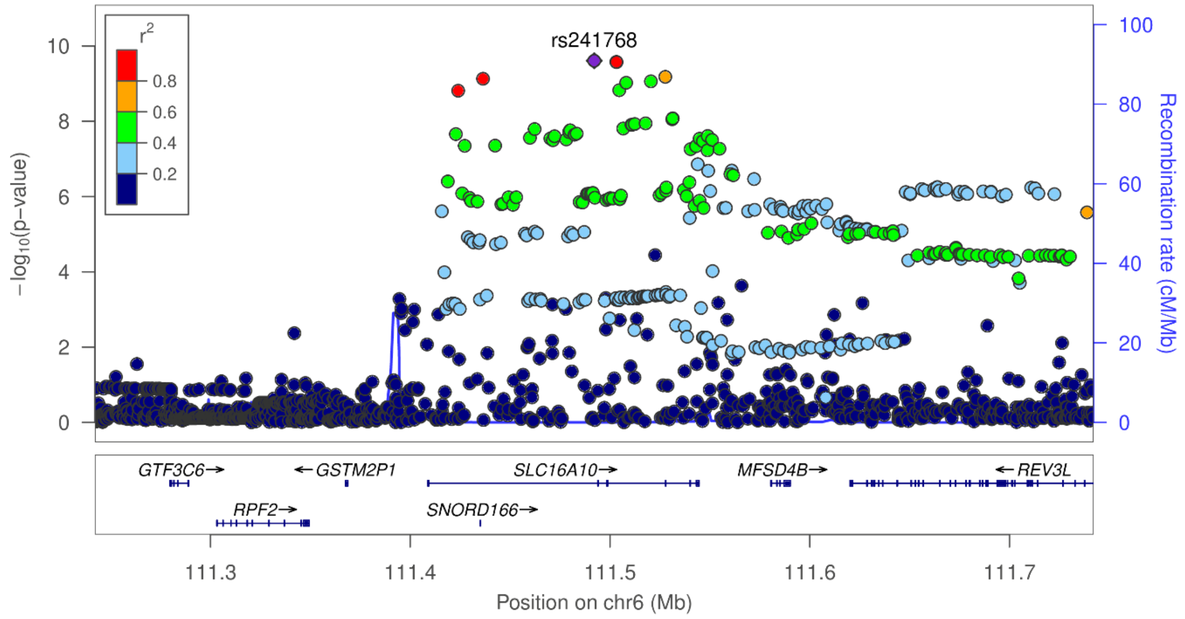

Quinic acid – rs2106727 (chr7:17,287,998:A:G)

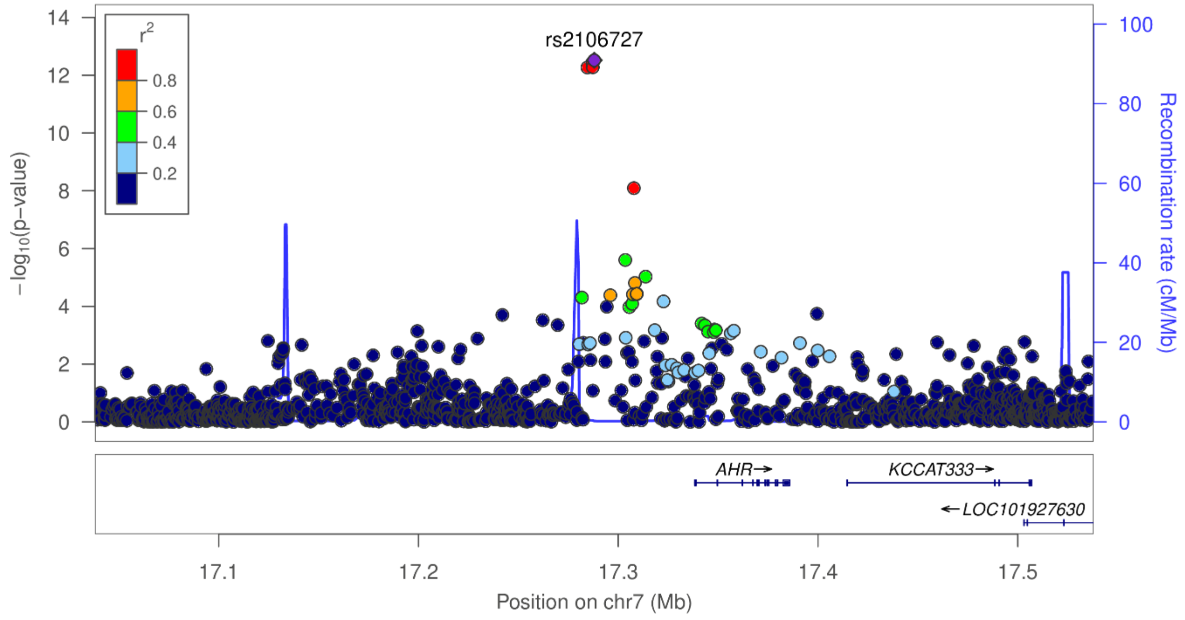

Trigonelline – rs2106727 (chr7:17,287,998:A:G)

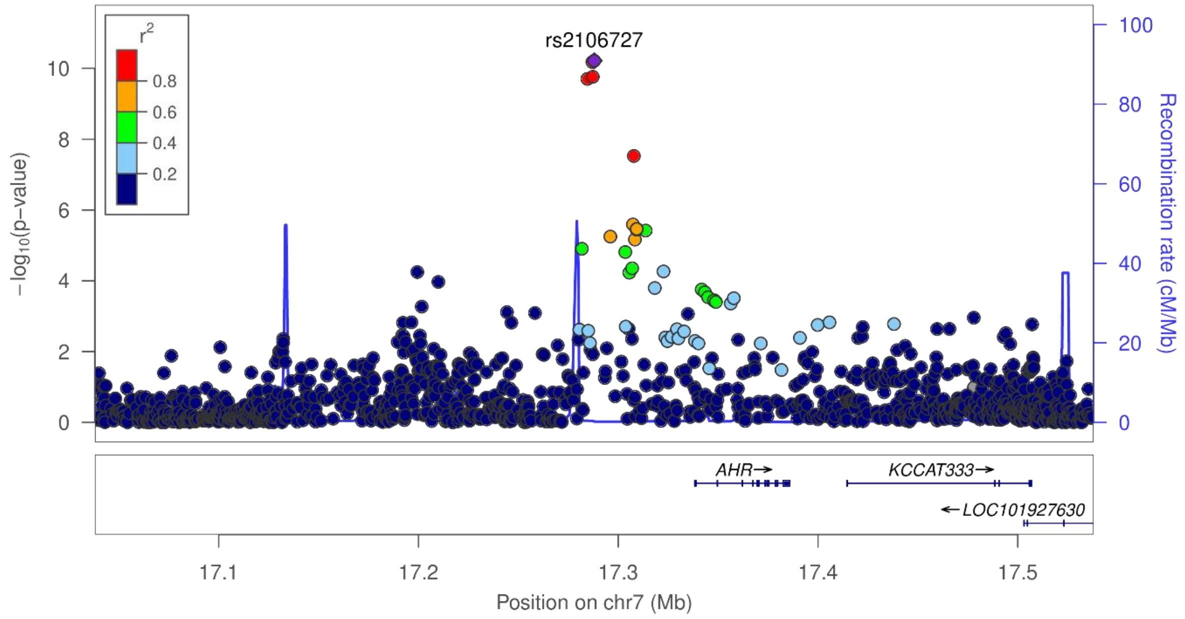

Formate – rs4921913 (chr8:18,272,377:T:C)

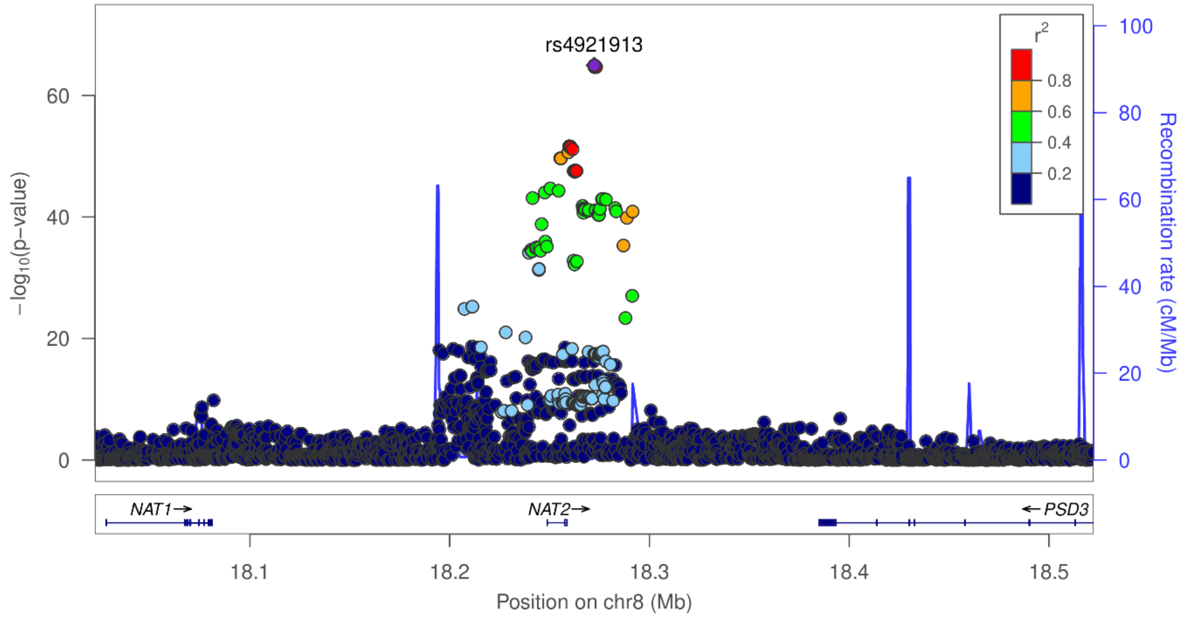

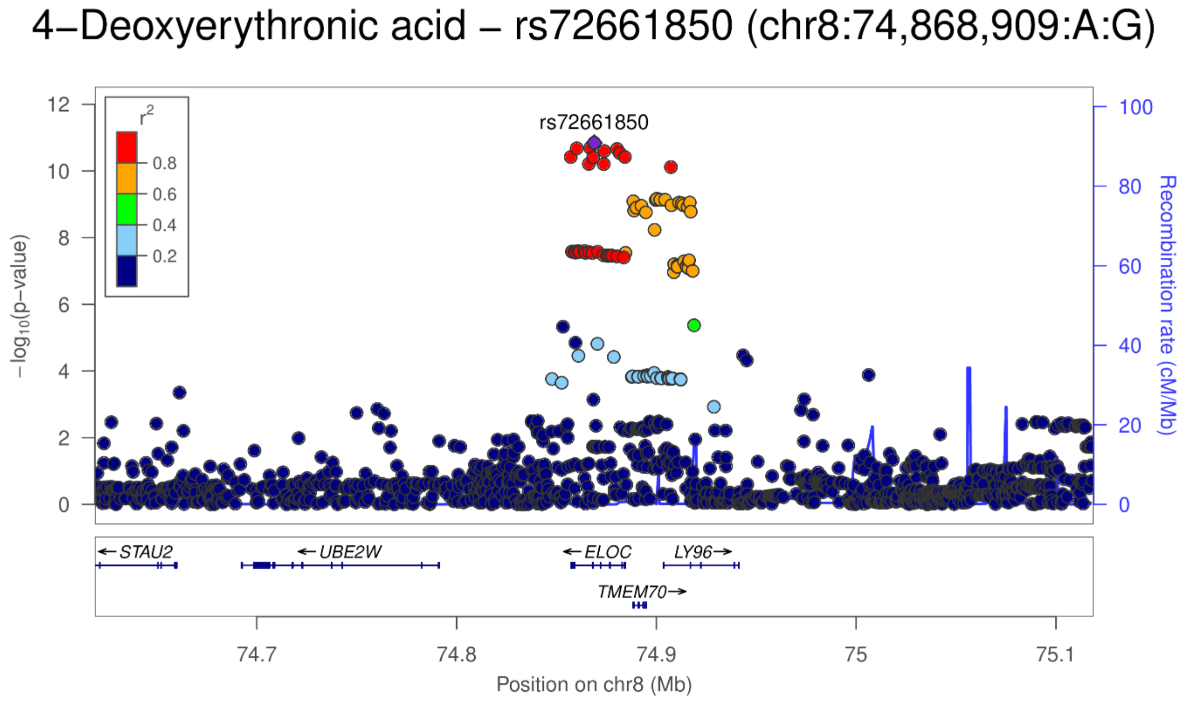
4−Deoxyerythronic acid − rs72661850 (chr8:74,868,909:A:G)
rs72661850
r2
0.8
0.6
0.4
0.2
−log10(p−value)
Recombination rate (cM/Mb)
STAU2
UBE2W
ELOC
LY96
TMEM70
74.7
74.8
74.9
75
75.1
Position on chr8 (Mb)

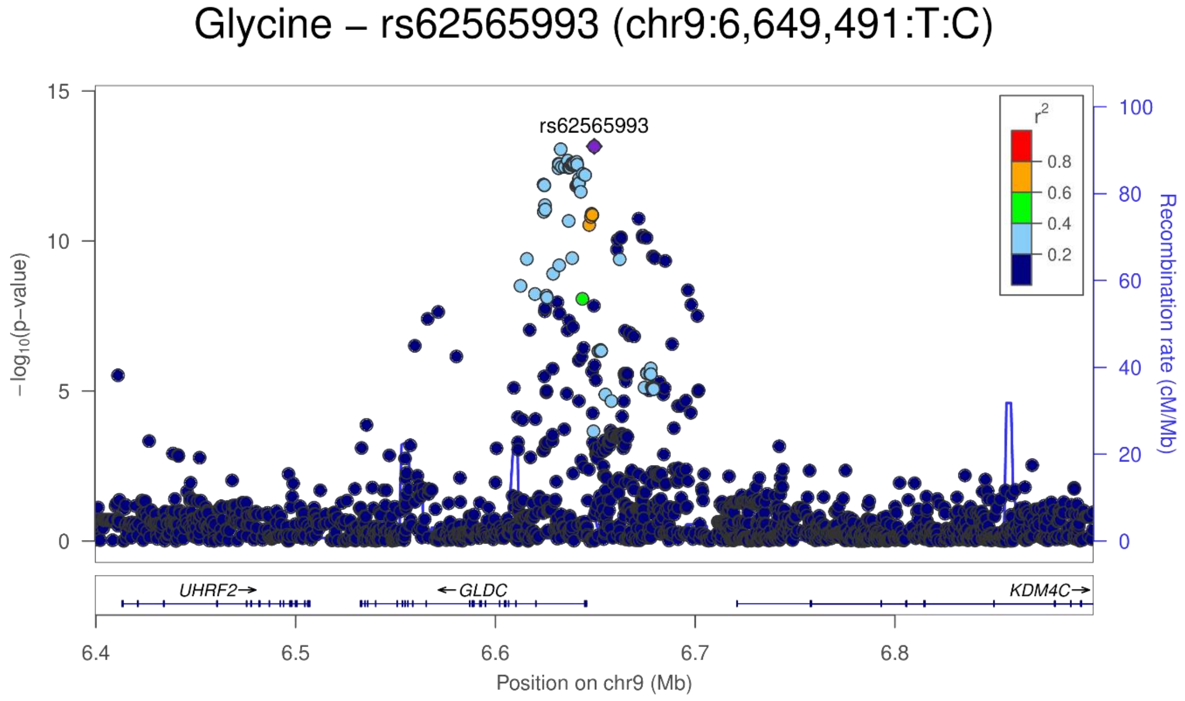
Glycine − rs62565993 (chr9:6,649,491:T:C)
rs62565993
r2
0.8
0.6
0.4
0.2
−log10(p−value)
Recombination rate (cM/Mb)
UHRF2
GLDC
KDM4C
6.4
6.5
6.6
6.7
6.8
Position on chr9 (Mb)

3−Hydroxyisobutyrate − rs2472479 (chr9:107,525,165:T:G)

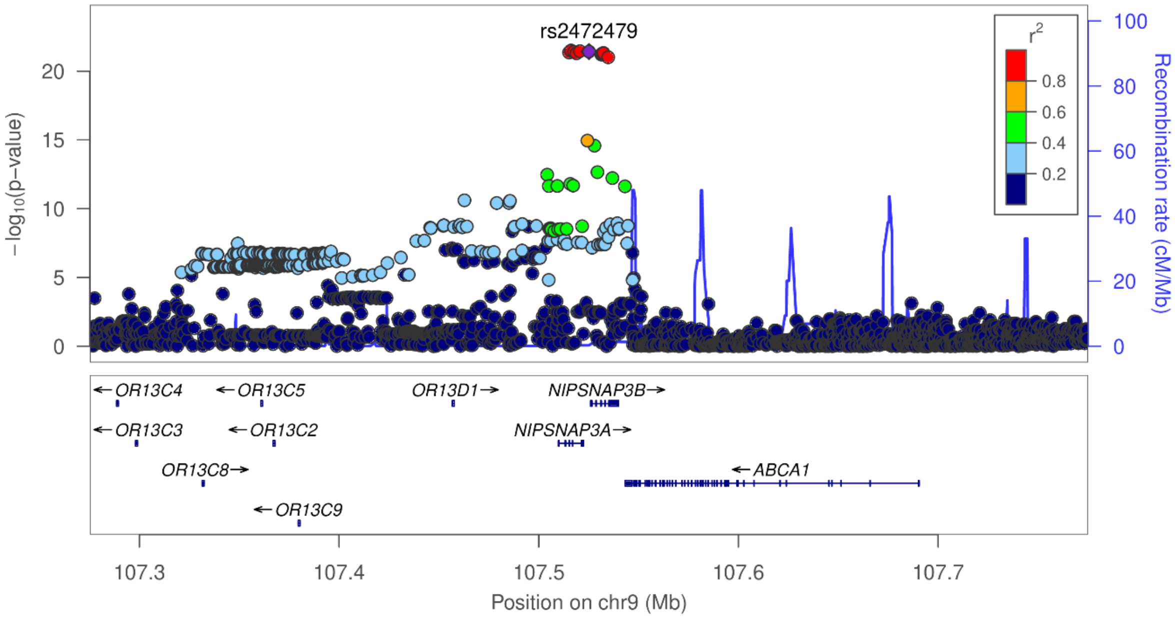

Xylose − rs550057 (chr9:136,146,597:T:C)

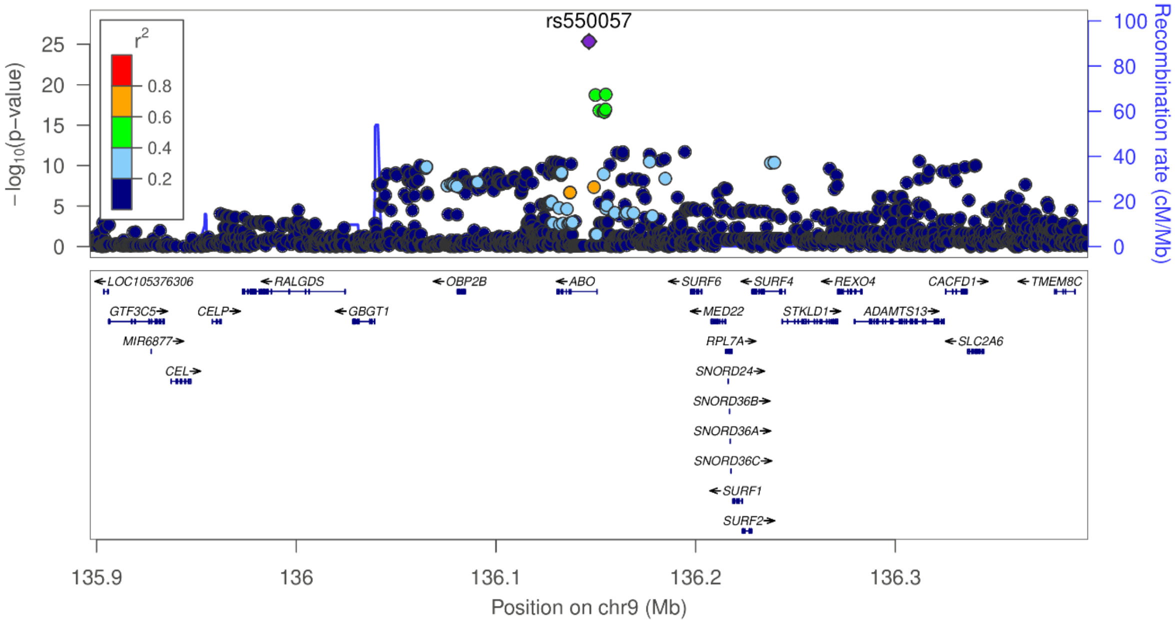

3−Aminoisobutyrate − rs2080403 (chr12:345,369:C:G)

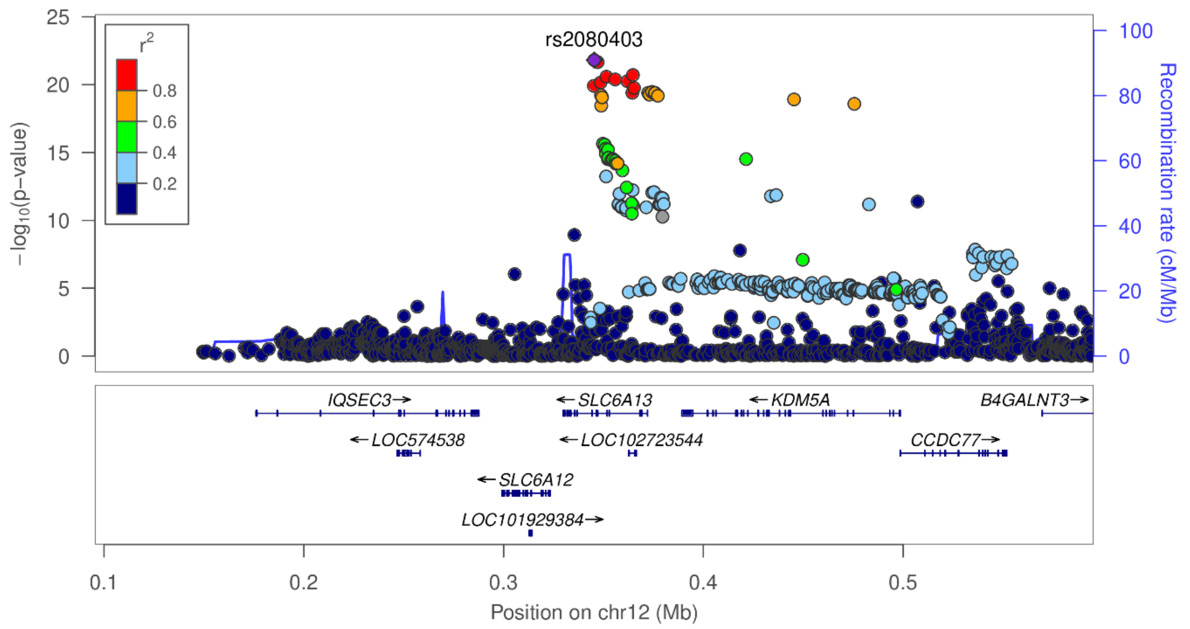

4−Deoxythreonate − rs78470967 (chr12:4,521,511:A:T)

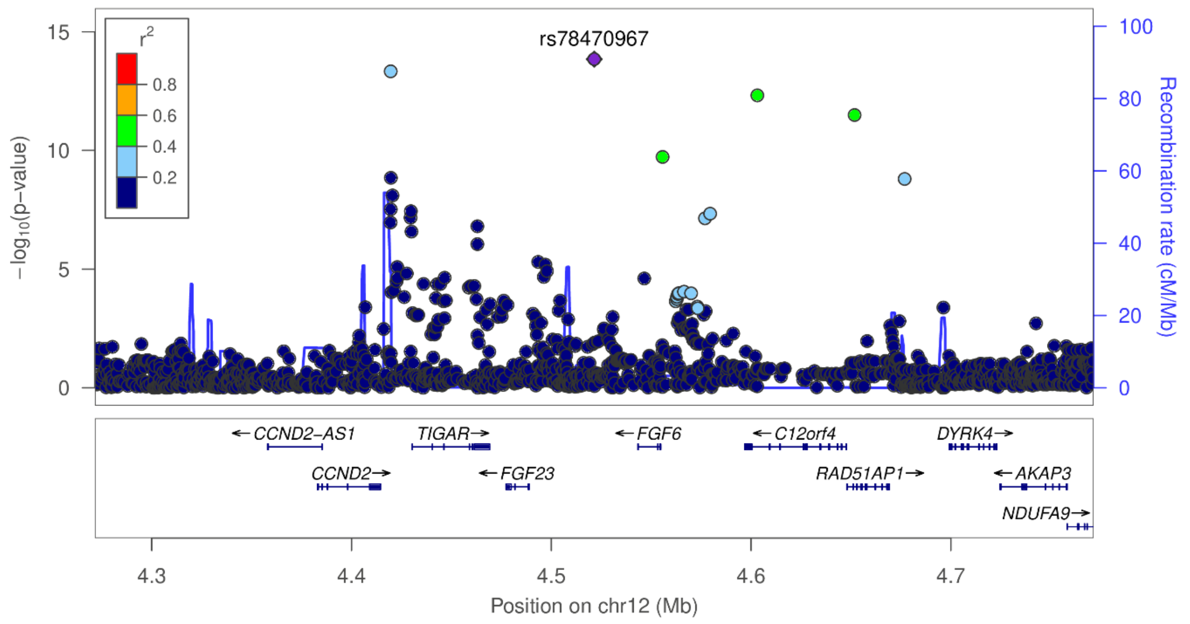

2−Hydroxyisobutyrate – rs1795967 (chr12:122,344,302:A:G)

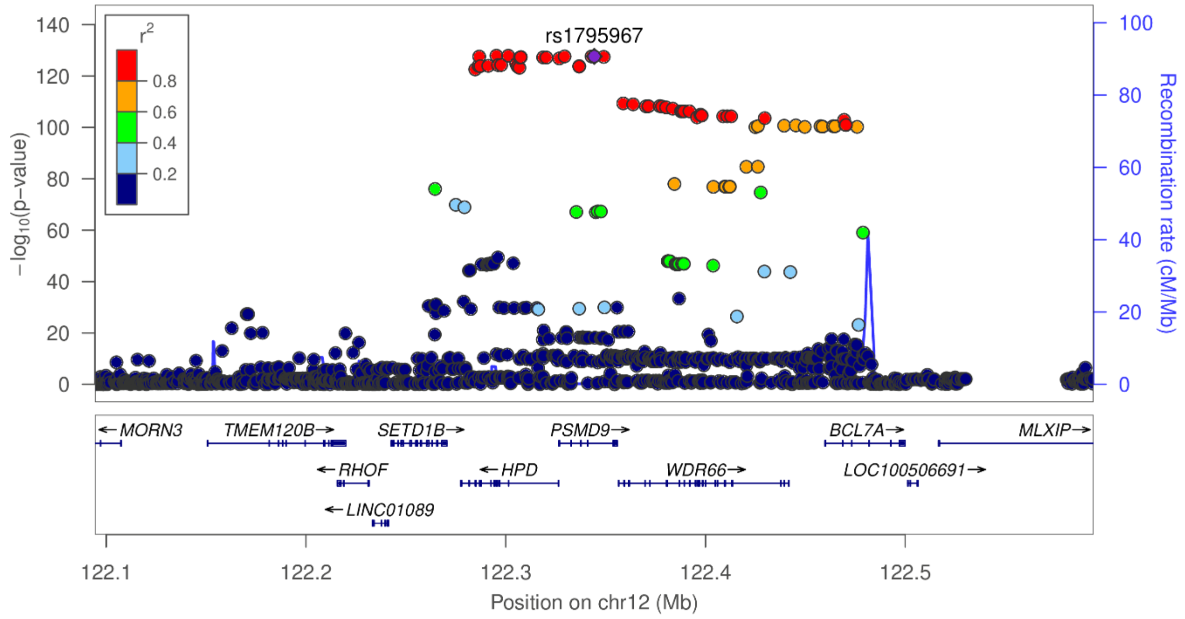

3−Hydroxyisovalerate – rs7499358 (chr16:20,557,634:A:T)

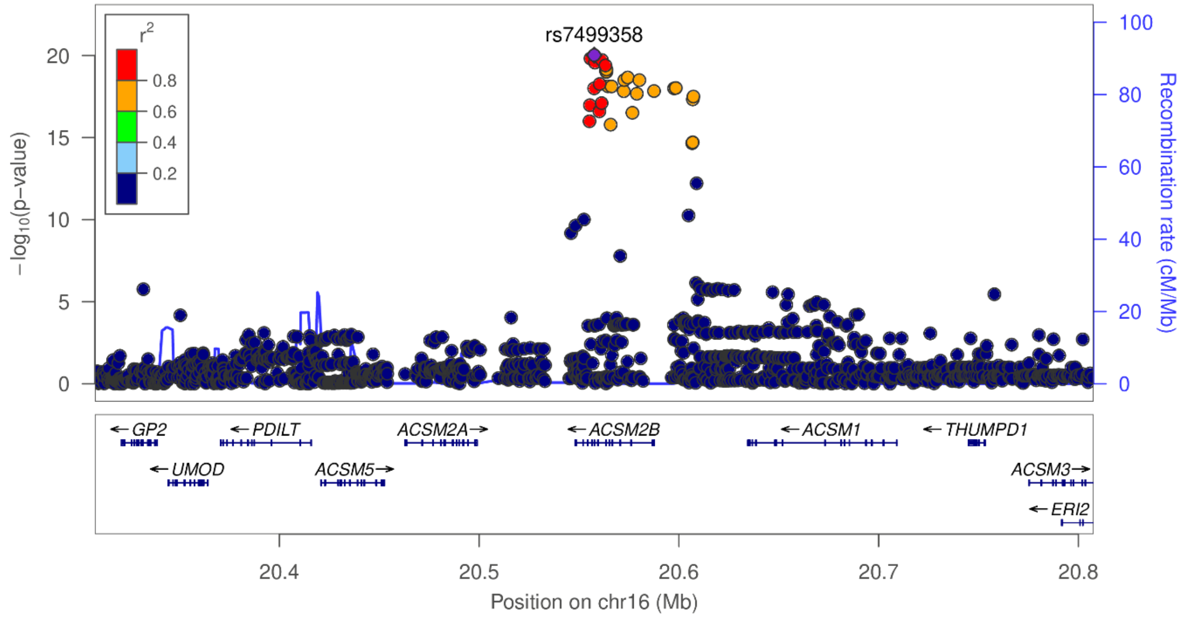

3−Hydroxyisovalerate – rs540815683 (chr16:20,608,891:C:G)

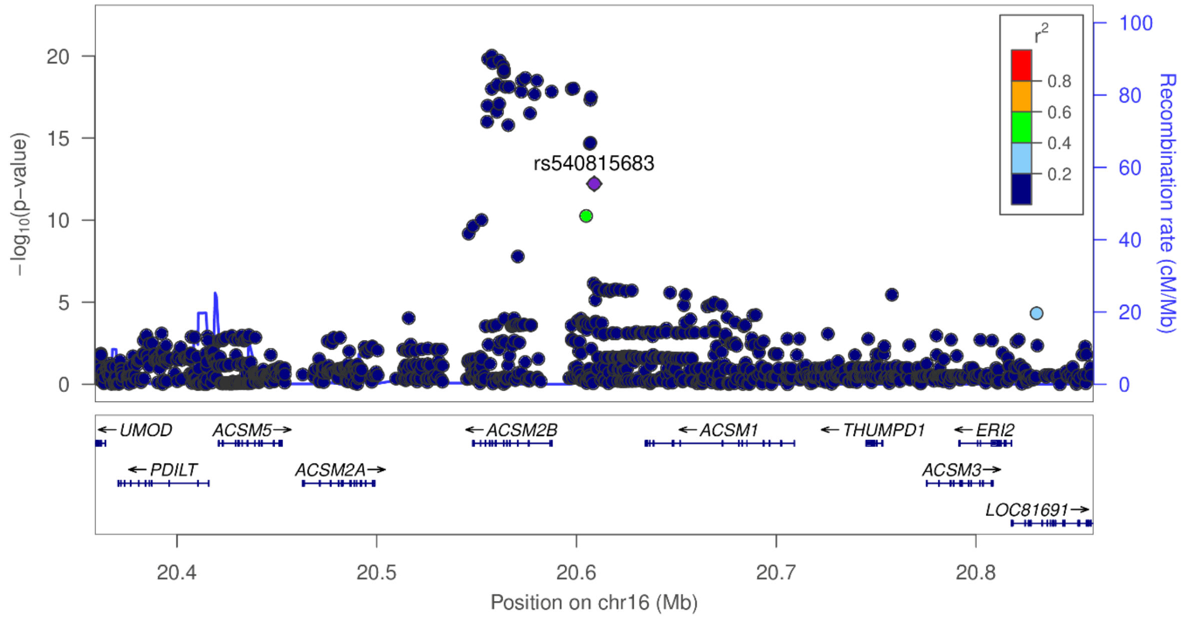

Citrate – rs11567842 (chr17:26,824,156:A:G)

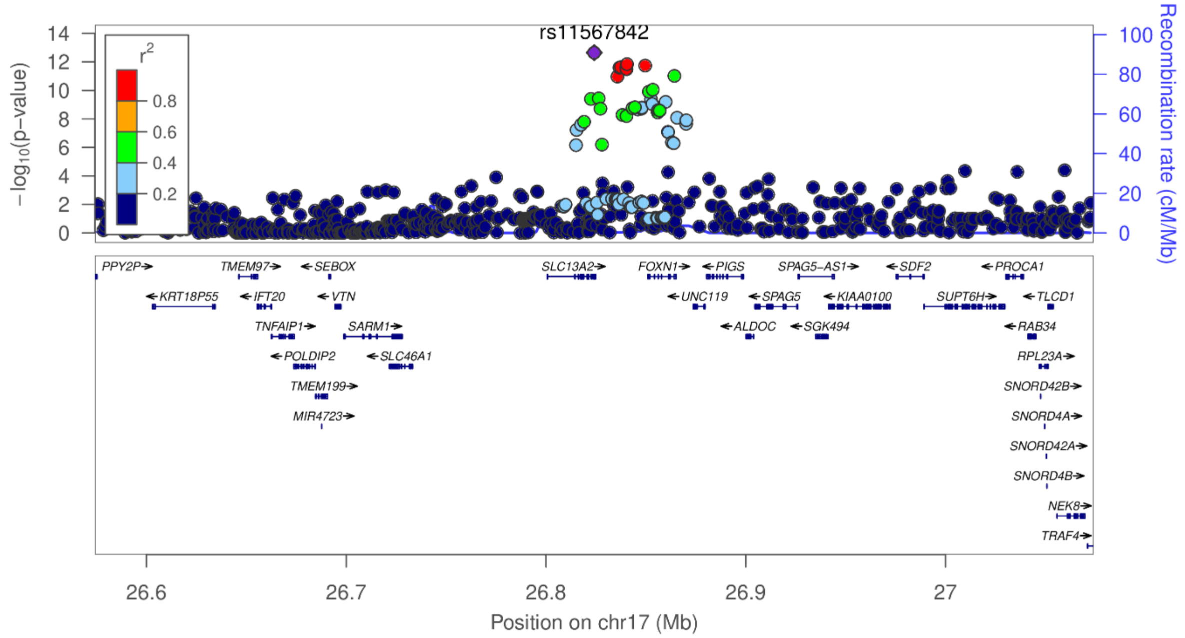

Threonine – rs11078902 (chr17:37,631,883:C:G)

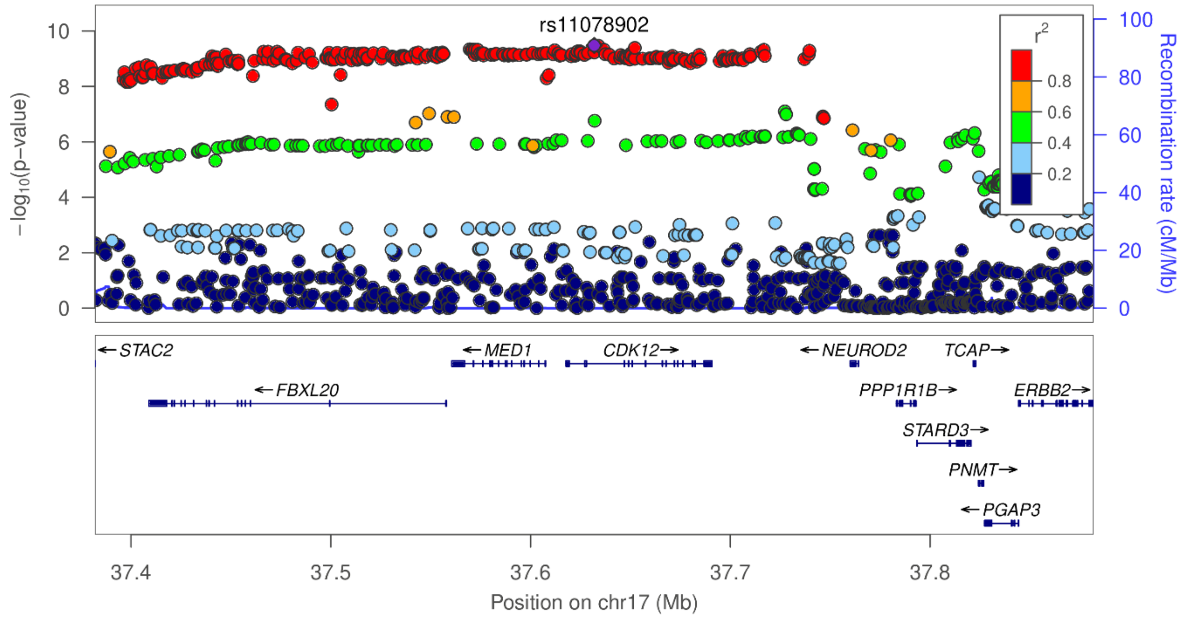

Tryptophan – rs12453397 (chr17:37,633,970:A:C)

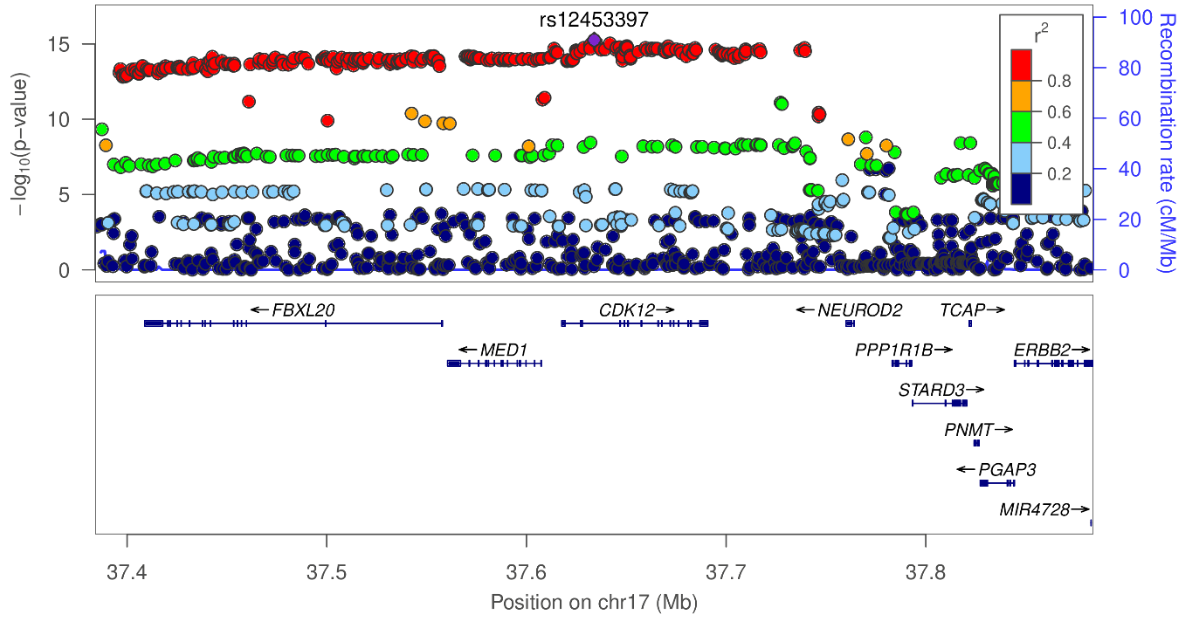

Tyrosine – rs12453397 (chr17:37,633,970:A:C)

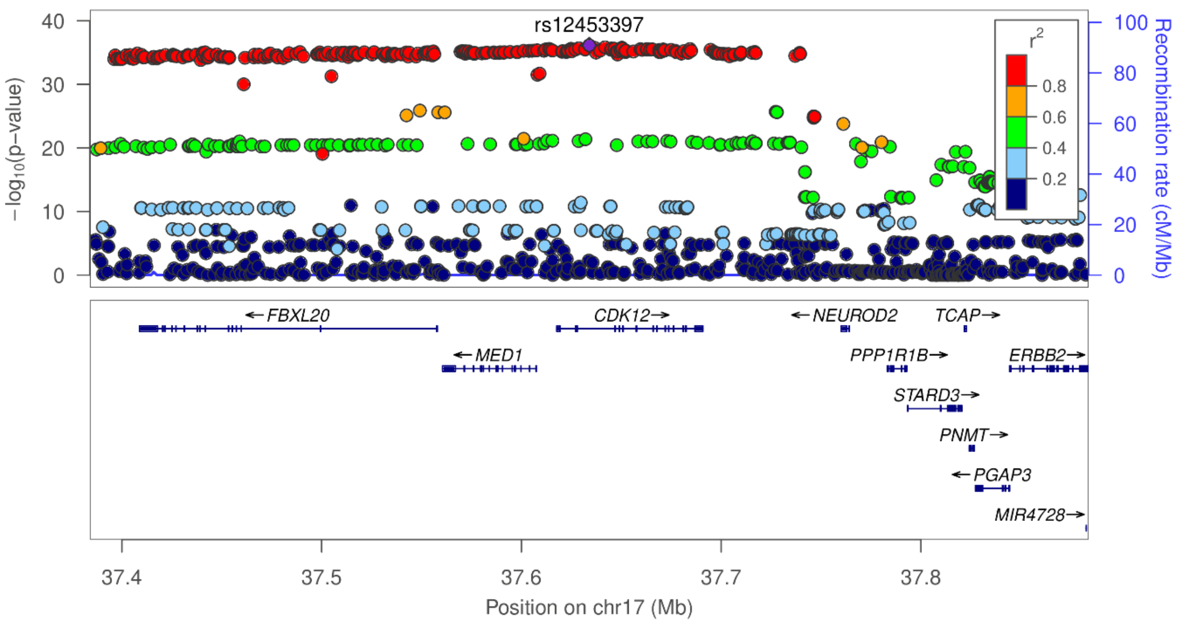

Histidine – rs4795371 (chr17:37,636,695:T:G)

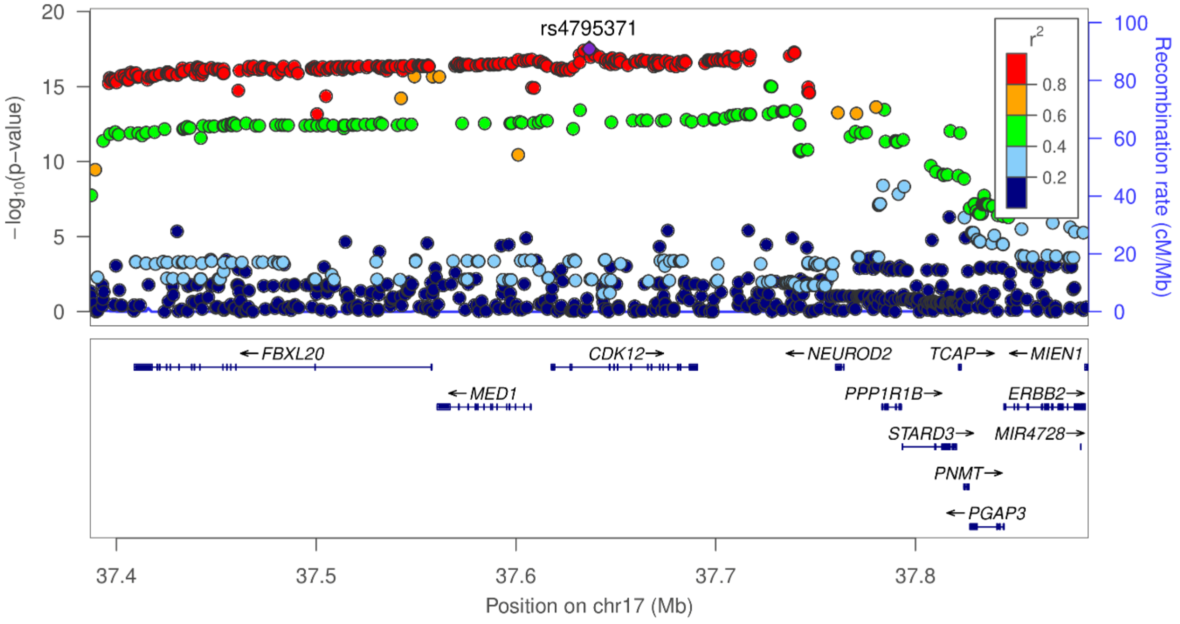

Supplementary Figure 3

Study specific effect estimates for urinary metabolite associations with between study heterogeneity. Effect estimates for urinary metabolite COJO association signals with $p<5\times10^{-8}$ and significant between study heterogeneity $p<0.05/86$ in the METAL meta-analysis (Supplementary Data 14). Full circle indicates $p<5\times10^{-8}$. Two-sided p-values calculated with METAL (meta-analysis) applying inverse variance weighted method and genomic control correction for the individual study level results, GCTA-COJO (meta-analysis) applying conditional and joint analysis of independently associated variants, SNPTest (FinnDiane) applying a score test with an additive model, or with RegScan (GS and VIKING).

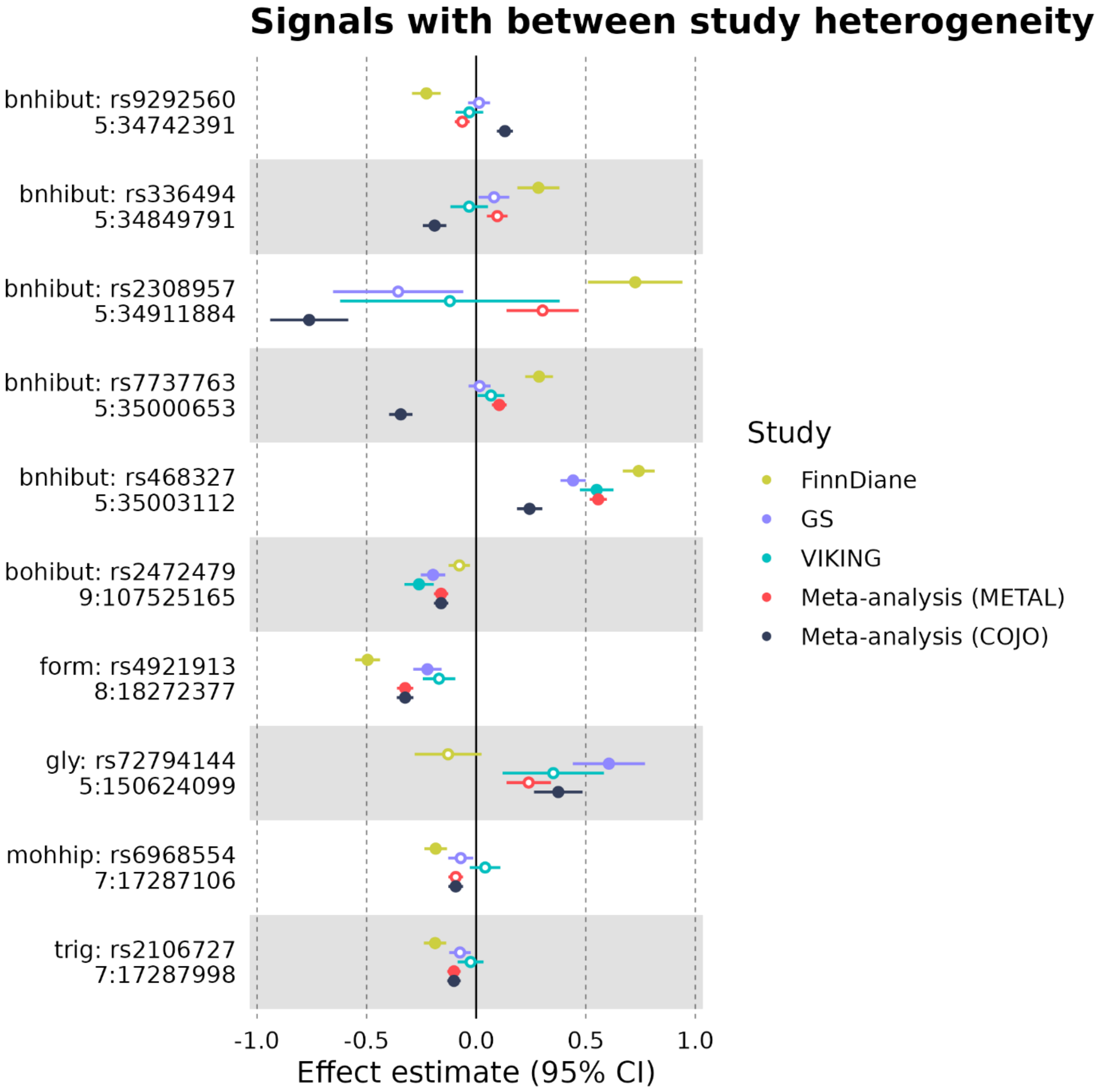

Supplementary Figure 4

METAL effect estimates at the COJO lead variants for the associated metabolite and creatinine. Two-sided p-values calculated with METAL (meta-analysis) applying inverse variance weighted method and genomic control correction for the individual study level results.

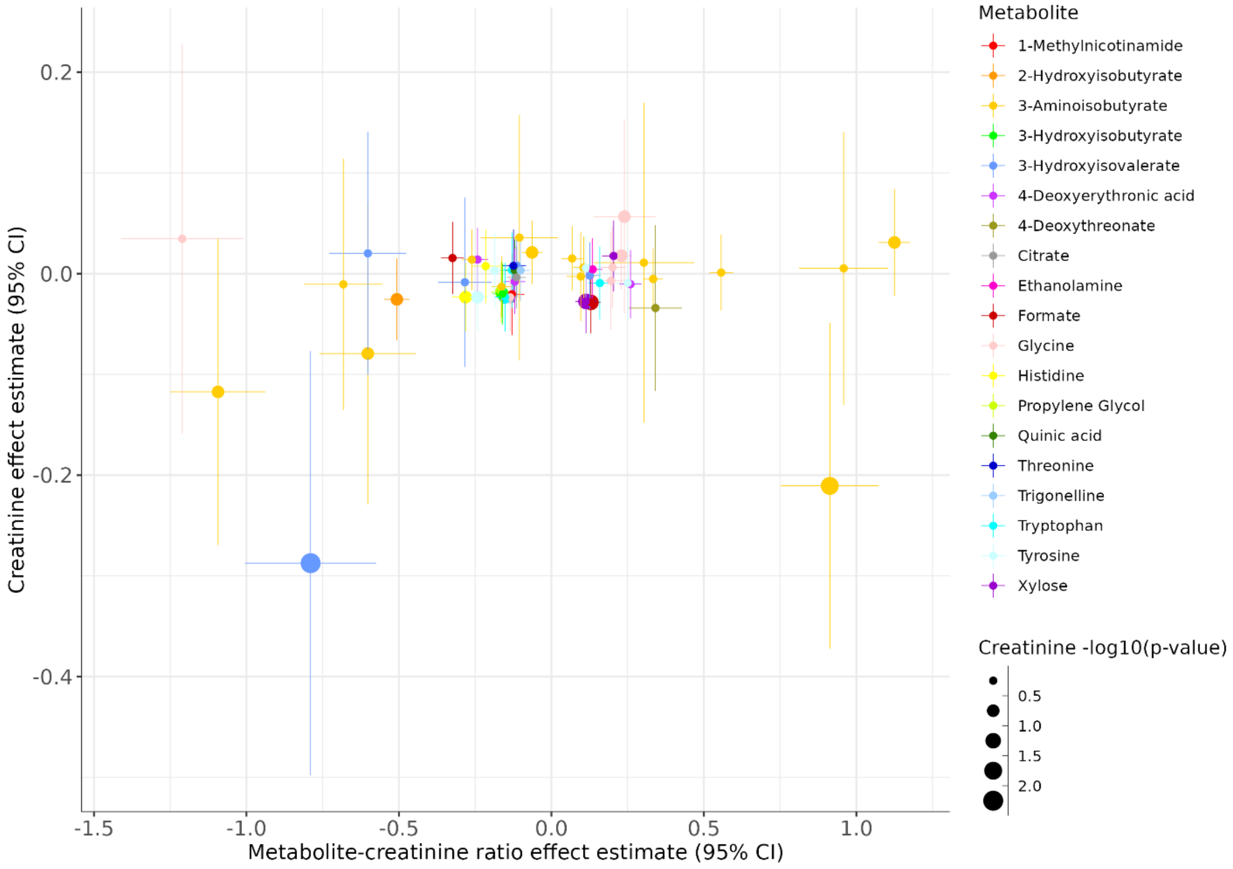

Supplementary Figure 5

***AGTX2* expression in the kidneys.** In single-cell RNA-Seq of human kidneys, *AGXT2* is expressed in the proximal convoluted tubules (PCT) in special. *AGXT2* encodes the alanine--glyoxylate aminotransferase 2. Data from Wilson et al.[10], figure obtained from http://humphreyslab.com/SingleCell/comparison.php.

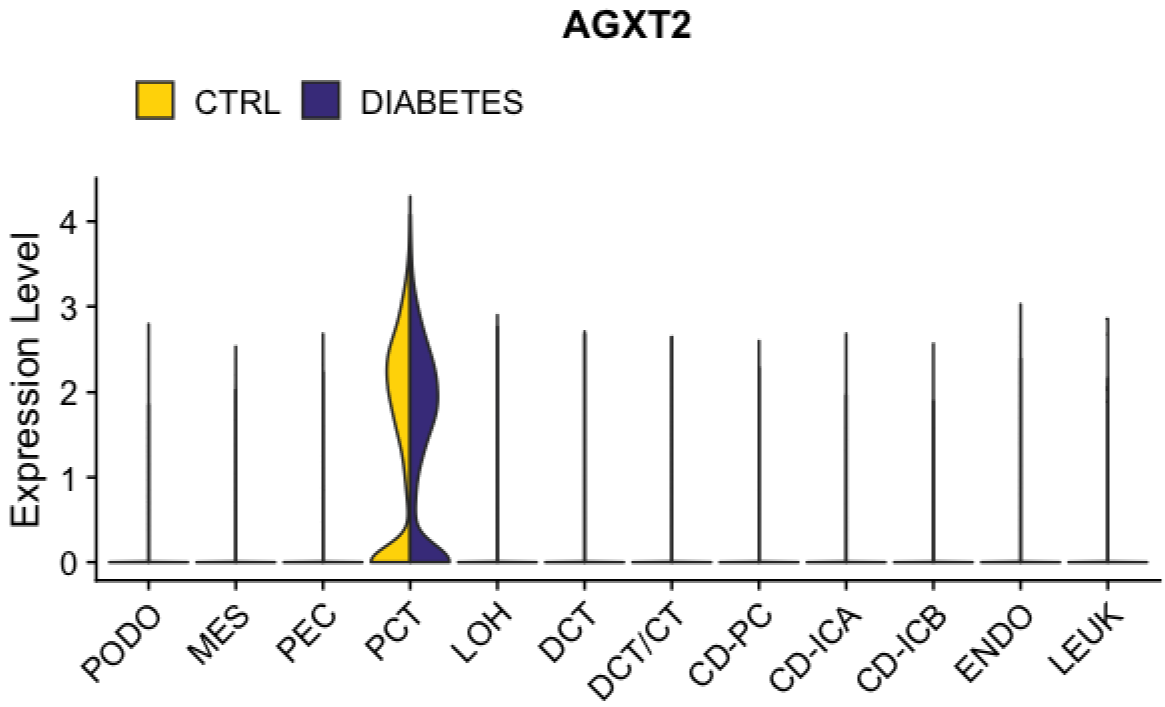

Supplementary Figure 6

Tyrosine GWAS signal vs. *SLC6A18* and *SLC6A19* kidney eQTL signals. Tyrosine signal p-values (two-sided p-values calculated with inverse variance weighted method, *n*=7,833 individuals) plotted against *SLC6A18* and *SLC6A19* eQTL p-values (downloaded from https://susztaklab.com/Kidney_eQTL/download.php) and coloured by r^2 values with either of the two independent tyrosine lead variants in the locus rs11133665 or rs7704882: A) *SLC6A18* rs11133665, B) *SLC6A18* rs7704882, C) *SLC6A19* rs11133665, and D) *SLC6A19* rs7704882. r^2 is the squared correlation coefficient between two variants.

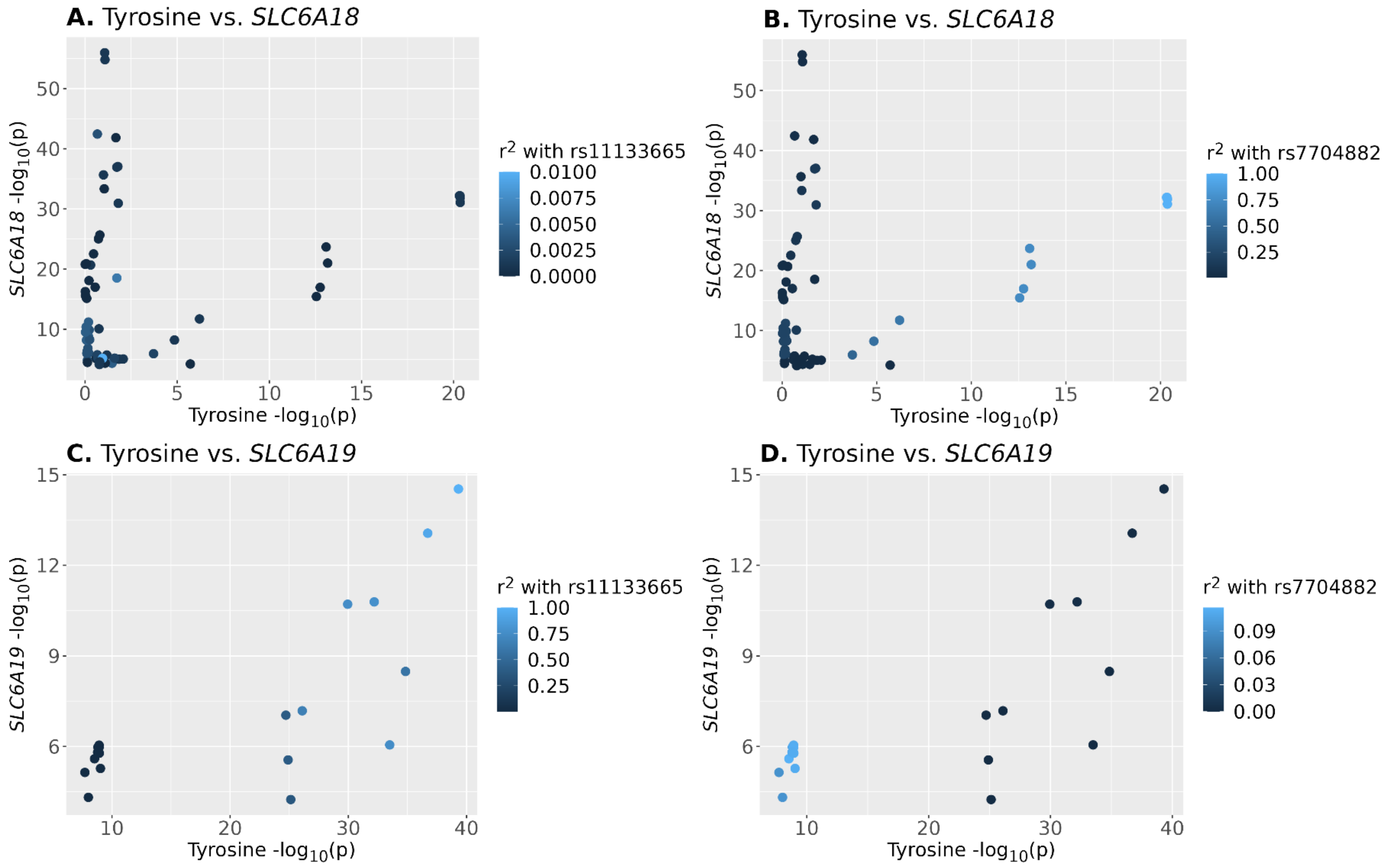

Supplementary Figure 7

Regional association with 3-aminoisobutyrate and blood and kidney eQTL target genes. A. LocusZoom plot of the 3-aminoisobutyrate signal (two-sided p-values calculated with inverse variance weighted method, *n*=6,347 individuals) centred around lead variant rs2080403. B. Blood eQTL association (queried from eQTLGen data set: https://eqtlgen.org/cis-eqtls.html and IEU GWAS database) for *SLC6A13* and *NINJ2* overlaid on top of the 3-aminoisobutyrate association signal. C. Kidney eQTL associations (downloaded from https://susztaklab.com/Kidney_eQTL/download.php) for *SLC6A13*, *AC007406.2,* and *CCDC77* overlaid on top of the 3-aminoisobutyrate association signal. The R2 (the squared correlation coefficient) values with rs11133665 are calculated based on 1000 Genomes phase 3 European population. Variants with no R2 information are not shown.

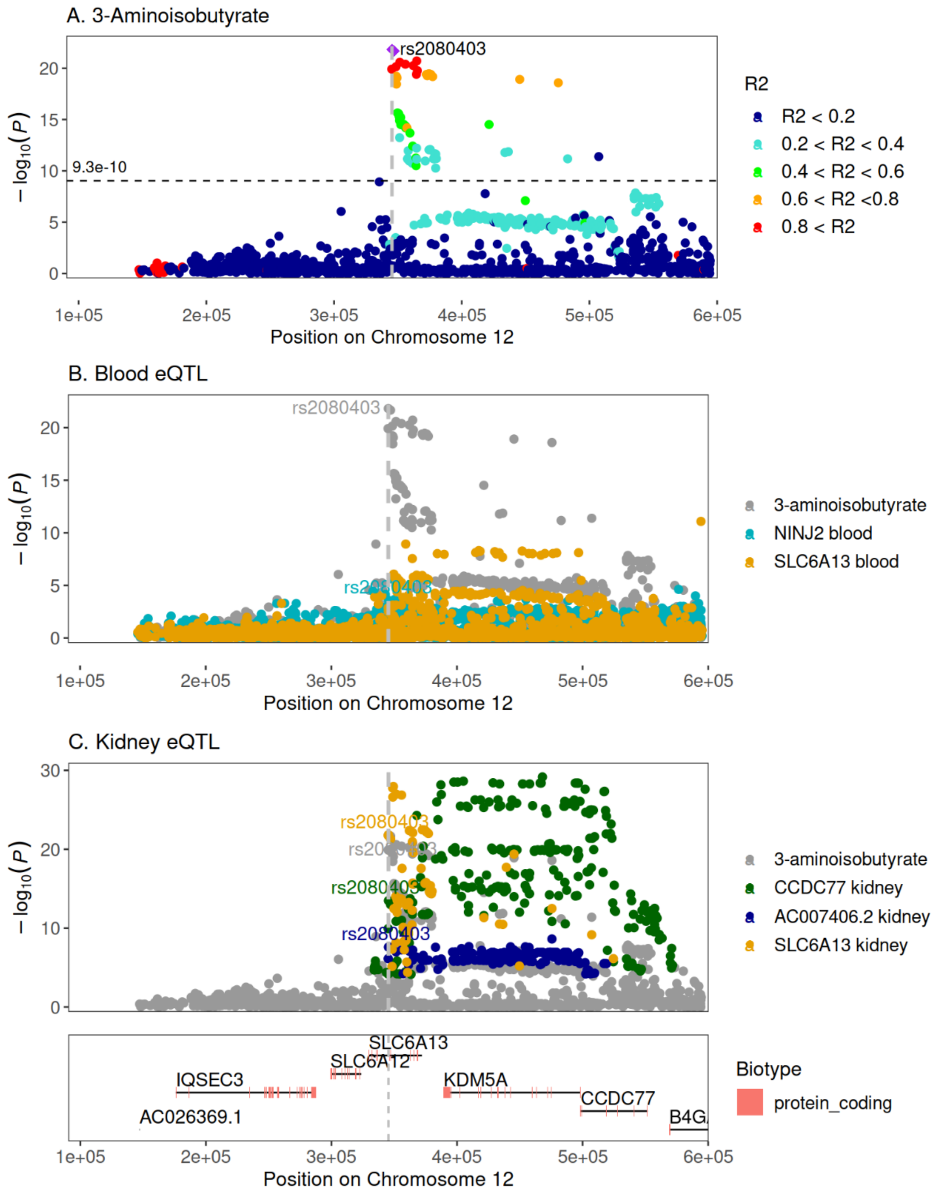

Supplementary Figure 8

Two-sample Mendelian Randomization results scatter plots for association of eGFR with urinary metabolites. Plots are shown for analysis in which the genetic instrument for eGFR was significantly associated ($p<4.6\times10^{-4}$, Supplementary Data 9) with a urinary metabolite. The plots include the eGFR effect estimate on the x-axis, the metabolite effect estimate on the y-axis, and lines showing the causal effect estimates for the different methods listed in the figure legend. The error bars give the standard errors of the estimates. Ala: alanine. Aohibut: 2-hydroxyisobutyrate. Bohival: 3-hydroxyisovalerate. Etnh: ethanolamine. Form: formate. Gln: glutamine. Gly: glycine. Glya: glycolic acid. Leu: leucine. Pglu: pyroglutamate. Pseur: pseudouridine. Ura: uracil. Val: valine.

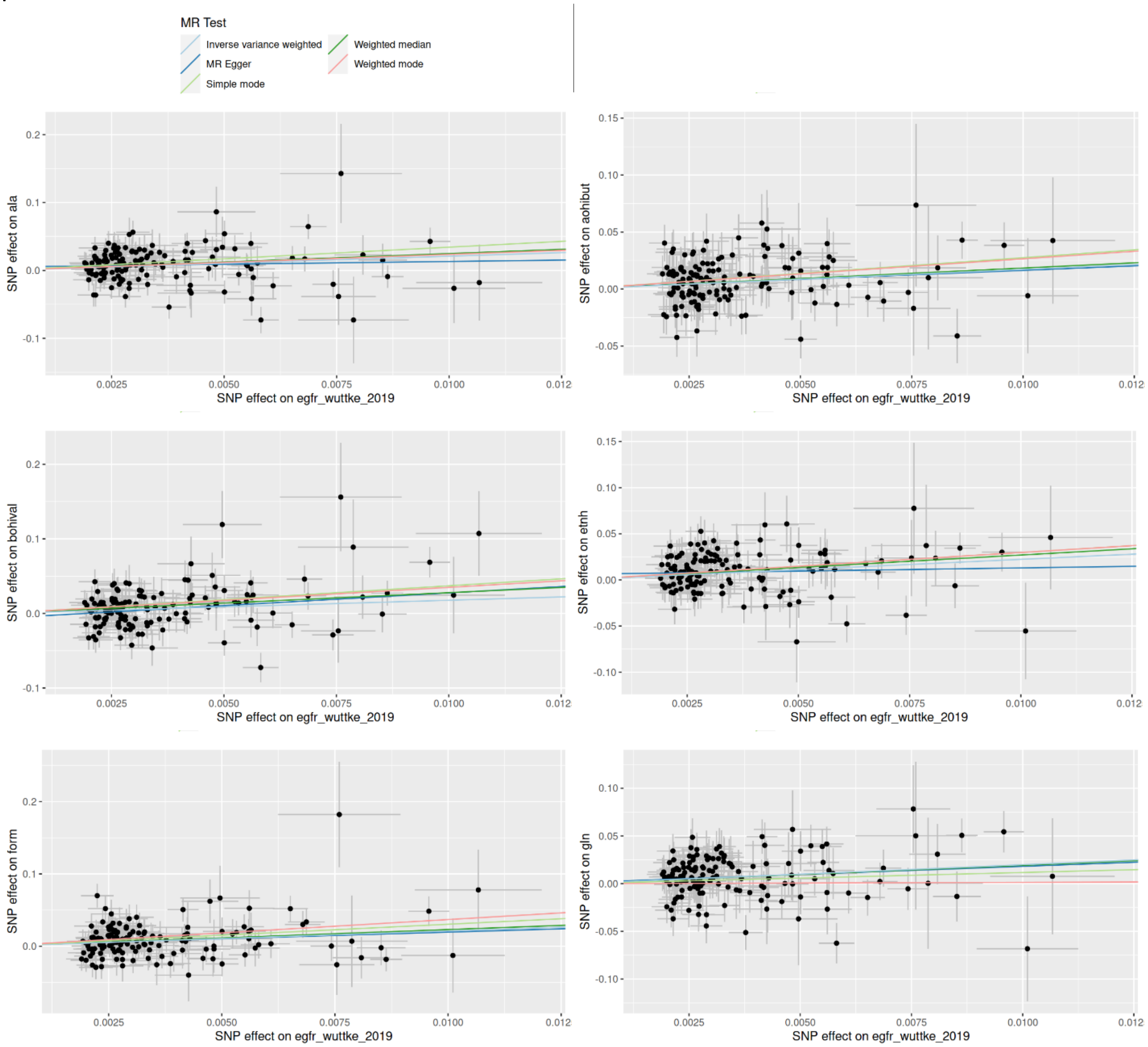

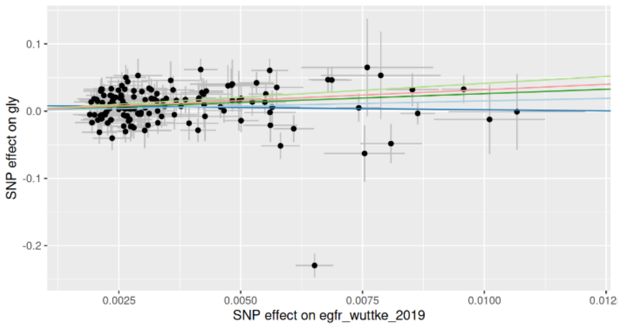
SNP effect on gly
0.1
0.0
-0.1
-0.2
0.0025
0.0050
0.0075
0.0100
0.012
SNP effect on egfr_wuttke_2019

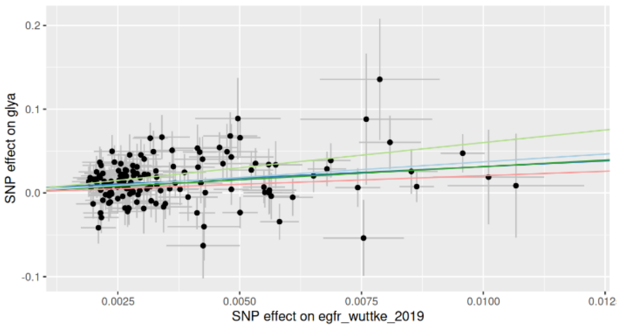
SNP effect on glya
0.2
0.1
0.0
-0.1
0.0025
0.0050
0.0075
0.0100
0.012
SNP effect on egfr_wuttke_2019

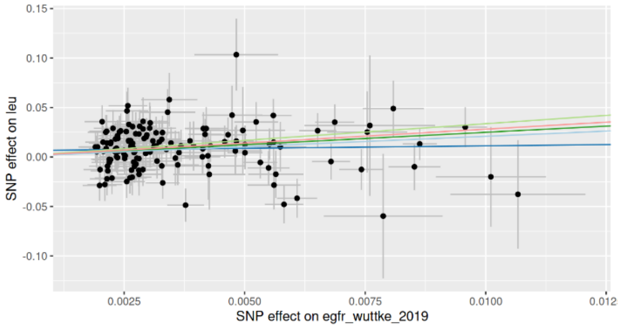
SNP effect on leu
0.15
0.10
0.05
0.00
-0.05
-0.10
0.0025
0.0050
0.0075
0.0100
0.012
SNP effect on egfr_wuttke_2019

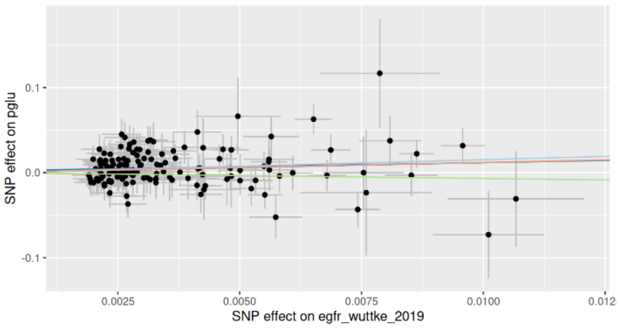
SNP effect on pglu
0.1
0.0
-0.1
0.0025
0.0050
0.0075
0.0100
0.012
SNP effect on egfr_wuttke_2019

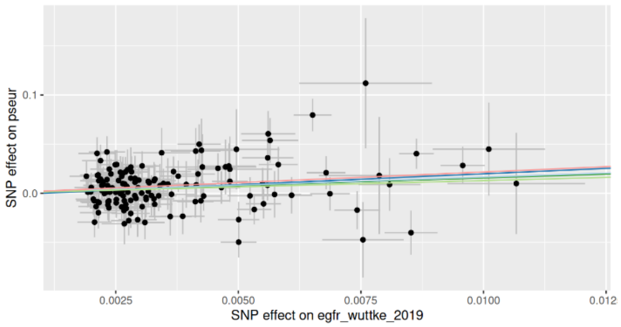
SNP effect on pseur
0.1
0.0
0.0025
0.0050
0.0075
0.0100
0.012
SNP effect on egfr_wuttke_2019

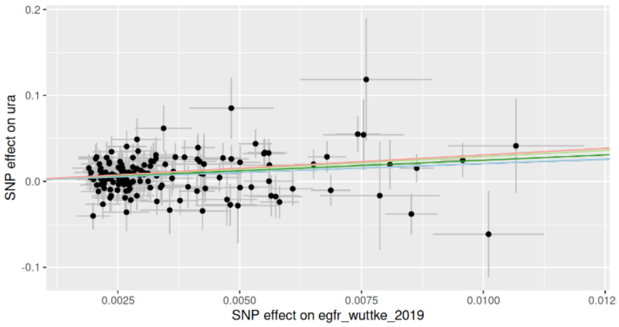
SNP effect on ura
0.2
0.1
0.0
-0.1
0.0025
0.0050
0.0075
0.0100
0.012
SNP effect on egfr_wuttke_2019

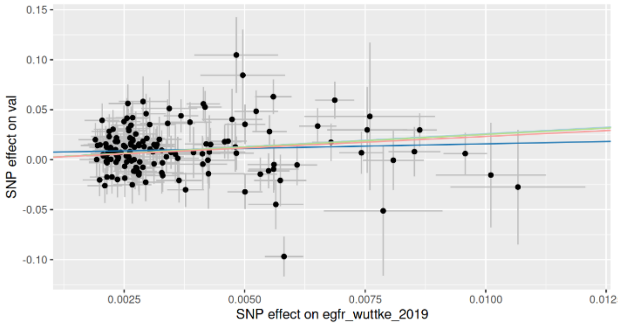
SNP effect on val
0.15
0.10
0.05
0.00
-0.05
-0.10
0.0025
0.0050
0.0075
0.0100
0.012
SNP effect on egfr_wuttke_2019

Supplementary Figure 9

Effect of covariate adjustment on the metabolite associations in FinnDiane. Effect estimates for the COJO lead signals in the FinnDiane GWAS data with imputation quality > 0.4 (n=53 variants). A. Effect of eGFR adjustment: original model (MIN, adjusted for age, sex, genotyping batch, and genetic principal components) vs. MIN model adjusted for eGFR (MIN + eGFR). B. Effect of additional age and sex adjustment: MIN model vs. MIN model adjusted for the second time for age and sex after the inverse normal transformation (MIN + age and sex). *R*: the Pearson correlation coefficient of the effect estimates. The effect estimates derived from a score test with an additive model.

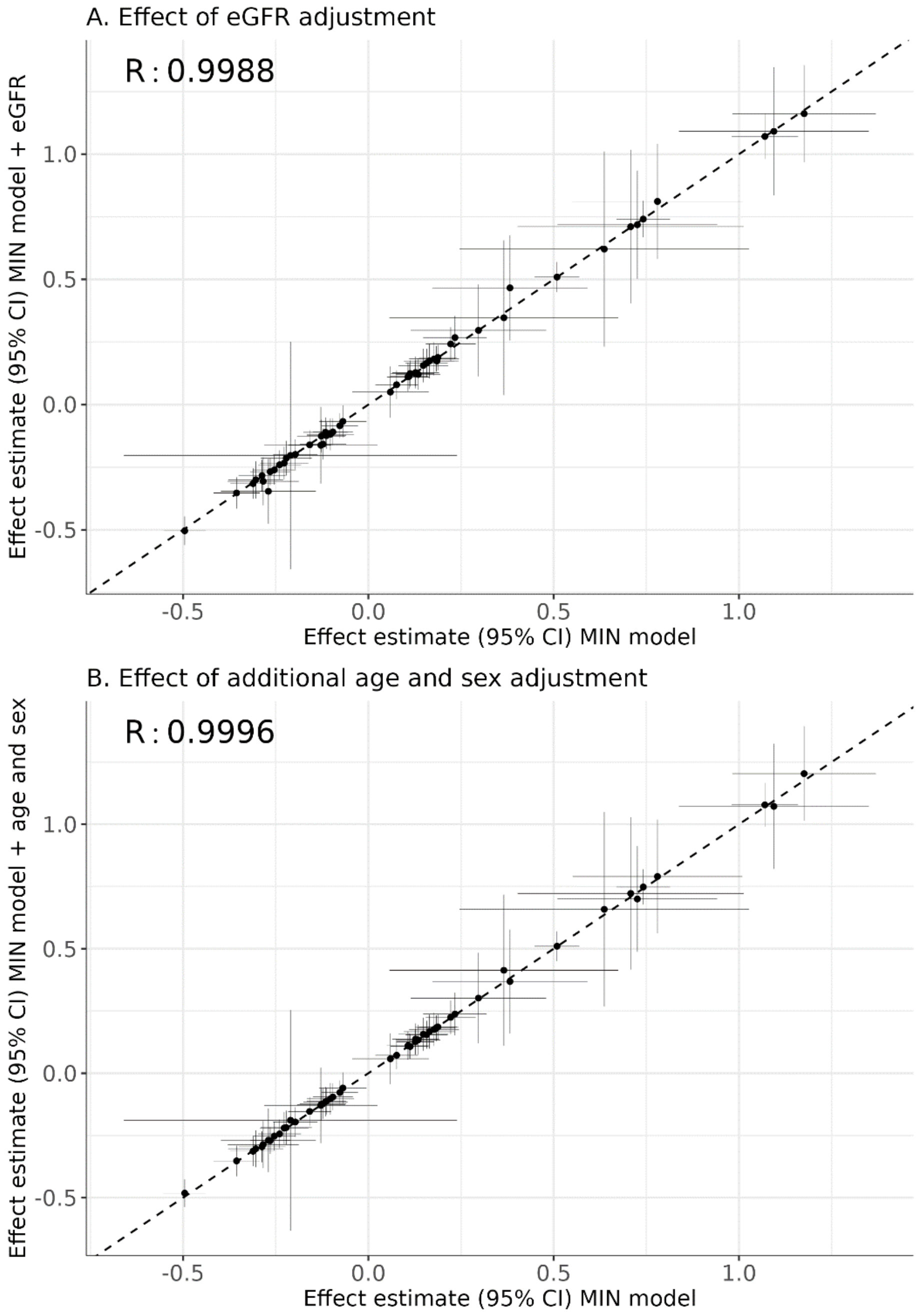

Supplementary Figure 10
Participant selection criteria in FinnDiane, VIKING and Generation Scotland studies. ESKD: end-stage kidney disease. T1D: type 1 diabetes.

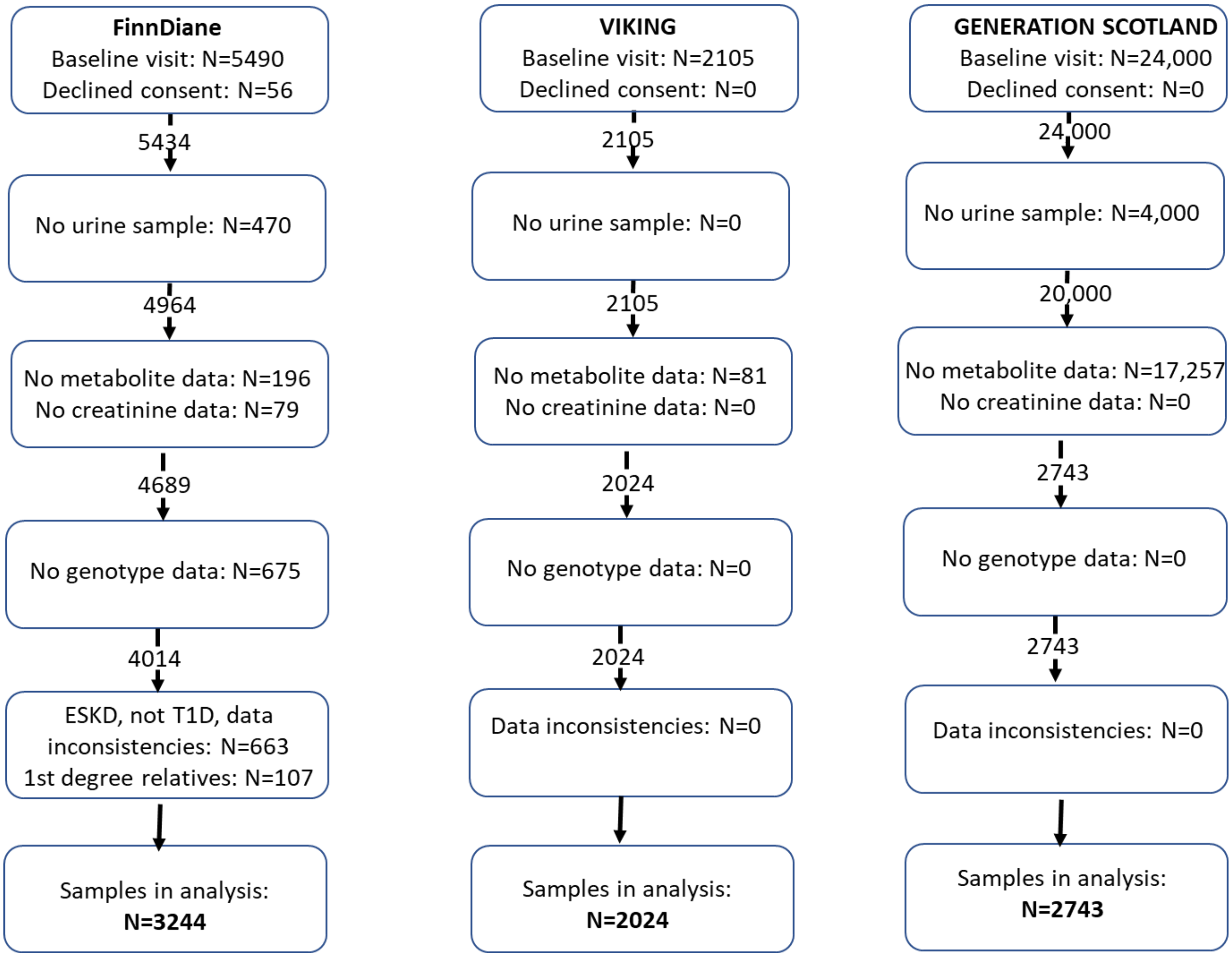

Supplement: Supplementary file 1 — Supplementary Information [file 41467_2024_55182_MOESM1_ESM.pdf]
