# Supplementary material for: Genome-wide characterization of 54 urinary metabolites reveals molecular impact of kidney function
Source: Nat Commun. 2025 Jan 2;16:325. doi: 10.1038/s41467-024-55182-1 (PMC11696681; doi:10.1038/s41467-024-55182-1)
Supplement: Supplementary file 2 — Description of Additional Supplementary Files [file 41467_2024_55182_MOESM2_ESM.pdf]

Description of Additional Supplementary Files

File Name: Supplementary Data

Description:

Supplementary Data 1: Urinary metabolite characteristics in FinnDiane, GS and VIKING. Metabolite values are given as metabolite to creatinine ratios for all metabolites except creatinine.

Supplementary Data 2: Urinary metabolite names and database ids.

Supplementary Data 3: Heritability estimates for the urinary metabolites. P-values were calculated for FinnDiane using the GCTA-GREML method, and for GS and VHS using a variance component model available within the RegScan GWAS pipeline. The meta-analysis two-sided p-value was calculated applying a random-effects model utilizing the inverse variance method and the between study heterogeneity p-value was calculated with a Q-test.

Supplementary Data 4: Genome-wide significant associations with urinary metabolites. Associations (n=54) of variants with metabolites with two-sided $p < 9.3\times10^{-10}$ from the COJO analysis. Previously reported associations fetched from the GWAS catalog (window size=-/+500kb, $r^2 > 0.8$, and $p < 5\times10^{-8}$).

Supplementary Data 5: Replication results for the metabolite associations not previously reported in GWAS catalog (Table 1 of the manuscript).

Supplementary Data 6: Blood and kidney eQTLs at the COJO lead variants. Expression quantitative trait loci (eQTL) target genes in whole blood (two-sided $p < 5.4\times10^{-05}$) and in kidney, glomeruli and tubule (two-sided $p < 5.3\times 10^{-4}$) at the COJO lead signals.

Supplementary Data 7: MAGMA v1.6 gene set analysis for “Curated gene sets” and “GO terms”. The analysis was performed using the MAGMA gene-set analysis Implemented in FUMA. Gene sets that remain significant after Bonferroni correction (one-sided $p<3\times10^{-6}$) for the metabolite-wise analysis are shown. Genes in bold cursive were nominally associated with the metabolite ($p<0.05$) in the MAGMA gene analysis.

Supplementary Data 8: FUMA GENE2FUNC gene set analysis results. The gene set enrichment was tested using a hypergeometric test implemented in the FUMA GENE2FUNC process. Gene sets were considered significant if FDR (Benjamini-Hochberg) adjusted one-sided $p<0.05$ and the gene set included at least 2 genes annotated to variants associated with the urinary metabolite.

Supplementary Data 9: Mendelian randomization analysis of kidney trait exposure and BMI exposure on urinary metabolites. Two sample Mendelian randomization analysis results using eGFR, UACR or BMI as exposures and urinary metabolites as outcomes. Exposure-metabolite pairs with two-sided $p < 3.1\times10^{-04}$ (0.05 / number of unique exposure-outcome pairs = 0.05 / 159) in at least one analysis are reported. For each analysed exposure-outcome pair all the performed analysis results are shown.

Supplementary Data 10: Two-sample Mendelian randomization analysis results using two different eGFR-IVs (egfr_bun and egfr_cys) and metabolites as outcomes. Egfr_bun and egfr_cys = all eGFR associated genetic variants (Wuttke et al) that also associated with BUN GWAS (egfr_bun, Wuttke et al. 2019) or eGFRcys GWAS (egfr_cys, Stanzick et al., 2021).

Supplementary Data 11: Two-sample Mendelian randomization analysis results using BMI as exposures and urinary metabolites as outcomes. Exposure-metabolite pairs with $p < 4.7\times10^{-04}$ (0.05 / number of unique exposure-outcome pairs = 0.05 / 106) regarded as significant.

Supplementary Data 12: Two-sample Mendelian randomization analysis results using metabolites as exposures and eGFR, BUN and eGFRcys as outcomes.

Supplementary Data 13: Genomic inflation factors for the urinary metabolite meta-analysis (METAL) results.

Supplementary Data 14: Instruments for the urinary metabolites in the two sample MR analysis. Variants (n=82) associated with metabolites in the COJO analysis with $p < 5.0\times10^{-08}$ and estimated F-statistic >10.
