# Supplementary material for: Genome-wide characterization of 54 urinary metabolites reveals molecular impact of kidney function
Source: Nat Commun. 2025 Jan 2;16:325. doi: 10.1038/s41467-024-55182-1 (PMC11696681; doi:10.1038/s41467-024-55182-1)
Supplement: Supplementary file 4 — Reporting Summary [file 41467_2024_55182_MOESM4_ESM.pdf]

nature portfolio

Corresponding author(s): Niina Sandholm, Caroline Hayward

Last updated by author(s): Oct 22, 2024

Reporting Summary

Nature Portfolio wishes to improve the reproducibility of the work that we publish. This form provides structure for consistency and transparency in reporting. For further information on Nature Portfolio policies, see our Editorial Policies and the Editorial Policy Checklist.

Statistics

For all statistical analyses, confirm that the following items are present in the figure legend, table legend, main text, or Methods section.

n/a	Confirmed	
☐	☒	The exact sample size (n) for each experimental group/condition, given as a discrete number and unit of measurement
☒	☐	A statement on whether measurements were taken from distinct samples or whether the same sample was measured repeatedly
☐	☒	The statistical test(s) used AND whether they are one- or two-sided<br>*Only common tests should be described solely by name; describe more complex techniques in the Methods section.*
☐	☒	A description of all covariates tested
☐	☒	A description of any assumptions or corrections, such as tests of normality and adjustment for multiple comparisons
☐	☒	A full description of the statistical parameters including central tendency (e.g. means) or other basic estimates (e.g. regression coefficient) AND variation (e.g. standard deviation) or associated estimates of uncertainty (e.g. confidence intervals)
☐	☒	For null hypothesis testing, the test statistic (e.g. F, t, r) with confidence intervals, effect sizes, degrees of freedom and P value noted<br>*Give P values as exact values whenever suitable.*
☐	☒	For Bayesian analysis, information on the choice of priors and Markov chain Monte Carlo settings
☒	☐	For hierarchical and complex designs, identification of the appropriate level for tests and full reporting of outcomes
☒	☐	Estimates of effect sizes (e.g. Cohen's d, Pearson's r), indicating how they were calculated

Our web collection on statistics for biologists contains articles on many of the points above.

Software and code

Policy information about availability of computer code

Data collection	GWAS data: Phasing: SHAPEIT (v2 r837), imputation: the Sanger Imputation Server (PBWT) and Minimac3 (v1.0.14)
Data analysis	GWAS analysis: RegScan (v 0.5) and SNPTest (v2.5.2), Meta-analysis study-level quality control: EasyQC R-package (v9.2), Meta-analysis: METAL (v 2011-03-25), Approximate conditional and joint GWAS analysis: GTCA-COJO (v1.93.2beta), LocusZoom plots: Locuszoom (v1.4), heritability analyses: GTCA tool (v1.93.2beta) and Regscan (v 0.5). Heritability heterogeneity analyses: R-package meta (v.6.5-0). Database (eQTL, GWAS catalogue) queries: R-package LDlinkR (v.1.2.3) and gwasrapidd (v.0.99.14), colocalization analyses: R-package coloc (v.5.1.0.1), enrichment analyses: FUMA (v.1.3.7) and MAGMA (v1.08). Mendelian Randomization: TwoSampleMR R package (v 0.6.4)

For manuscripts utilizing custom algorithms or software that are central to the research but not yet described in published literature, software must be made available to editors and reviewers. We strongly encourage code deposition in a community repository (e.g. GitHub). See the Nature Portfolio guidelines for submitting code & software for further information.

Data

Policy information about availability of data

All manuscripts must include a data availability statement. This statement should provide the following information, where applicable:

- Accession codes, unique identifiers, or web links for publicly available datasets
- A description of any restrictions on data availability
- For clinical datasets or third party data, please ensure that the statement adheres to our policy

The GWAS meta-analysis summary statistics for the 54 studied metabolites except glucose have been deposited in the GWAS Catalog under accessions codes GCST90451702–GCST90451754 (https://ebi.ac.uk/gwas).

For VIKING, the research data used in this study are available through managed access by application (accessQTL@ed.ac.uk), following approval by the QTL Data Access Committee with expected timeframe for response of about 2 months (https://viking.ed.ac.uk/). These data are available under managed access due to the consent given by the participants and Research Ethics Committee approvals. Each approved project is subject to a data or materials transfer agreement (D/MTA) or commercial contract. Data may be shared with academic or commercial recipients worldwide and may be used within the parameters of the Research Ethics Committee approvals. The individual-level GWAS and metabolite data for the Generation Scotland study are available under managed access for legal restrictions, access can be obtained by a reasonable proposal to the access committees of Generation Scotland; access@generationscotland.org; Each approved project is subject to a data or materials transfer agreement (D/MTA) or commercial contract. The raw individual-level data of the FinnDiane study are protected and are not available due to data privacy laws and participants written consent, which does not allow sharing of such data. The processed summary statistics for FinnDiane study can be obtained by a reasonable proposal to the corresponding author.

The GWAS summary statistics for general population kidney traits from the CKDGen consortium used in this study are available on the CKDGen project website (https://ckdgen.imbi.uni-freiburg.de); DKD phenotypes and T2D available in the T2D knowledge portal under "GENIE 2022 DKD GWAS" and "DIAMANTE T2D 2022 GWAS" (https://t2d.hugeamp.org/downloads.html); BMI available in the OpenGWAS database under accession code ukb-b-19953 (https://gwas.mrcieu.ac.uk/datasets/); asthma and coronary heart disease in the IEU GWAS database under finn-b-J10_ASTHMA and ieu-a-7, respectively (https://gwas.mrcieu.ac.uk/). The kidney eQTL data used in this study are available in the Human Kidney eQTL Atlas (https://susztaklab.com/Kidney_eQTL/download.php); and whole blood cis-eQTL data available on the eQTLGen website (https://eqtlgen.org/cis-eqtls.html).

Research involving human participants, their data, or biological material

Policy information about studies with human participants or human data. See also policy information about sex, gender (identity/presentation), and sexual orientation and race, ethnicity and racism.

Reporting on sex and gender	The genome-wide association study (GWAS) on metabolites was performed in three cohorts (n= 8,011) with a balanced number of men and women (3,816 men [47.6%] and 4,195 women[52.4%]). In total, 3,244 individuals were participants of the Finnish Diabetic Nephropathy Study (1,632 men/1,612 women) 2,743 of the Generation Scotland (1,373 men/1,370 women) and 2,207 of the VIKING study (811 men/ 1,213 women).Sex was assigned by the investigator at the baseline examination. Sex was included as a covariate in the GWAS analyses.
Reporting on race, ethnicity, or other socially relevant groupings	No socially constructed variables were used or included.
Population characteristics	The mean (standard error) age of the participants were 37.7 (12.2) years in FinnDiane, 55.8 (10.5) years in Generation Scotland and 49.8 (15.2) years in the VIKING study.
Recruitment	Generation Scotland (GS) study: family-based population study of 24,000 individuals from across Scotland: Individuals aged 35 to 65 years were recruited at random from 2006 to 2010 from collaborating medical practices. These participants then identified ≥first degree relatives who would also be able to participate (final age range: 18-98 years).<br><br>VIKING study: family-based population study of 2,108 individuals from the population isolate of the Shetland Isles in northern Scotland: volunteers aged ≥18 years with two or more grandparents born in the Shetland Isles were recruited from 2013 to 2015 by hearing of the study in a wide range of ways (events, broadcast, print ,media, word of mouth, social media, website etc).<br><br>FinnDiane study:ongoing (1997->) nation-wide multicentre study of >5,000 adults (aged ≥18 years) with typ 1 diabetes, focusing on diabetic complications. Individuals with type 1 diabetes were recruited from 93 centers all over Finland by physicians and nurses. The 93 centers included 5 university and 16 central hospitals, 33 district hospitals, and 26 primary health care centers across Finland and among the individuals asked to participate, the participation rate was 78% during the first years of the study.
Ethics oversight	The FinnDiane study protocol was approved by the Ethical Committee of the Helsinki and Uusimaa Hospital District (491/E5/2006, 238/13/03/00/2015, and HUS-3313-2018, July 3rd, 2019) and the participants gave their written informed consent before recruitment.<br><br>All the Viking Health Study – Shetland (VIKING) participants gave informed consent and the study was approved by the South East Scotland Research Ethics Committee, NHS Lothian (reference: 12/SS/0151).<br><br>All the GS:SFHS participant gave informed consent and the ethical approval for the study was obtained from the Tayside

Committee on Medical Research Ethics (on behalf of the National Health Service, reference 05/S1401/89).

Note that full information on the approval of the study protocol must also be provided in the manuscript.

Field-specific reporting

Please select the one below that is the best fit for your research. If you are not sure, read the appropriate sections before making your selection.

☒ Life sciences ☐ Behavioural & social sciences ☐ Ecological, evolutionary & environmental sciences

For a reference copy of the document with all sections, see nature.com/documents/nr-reporting-summary-flat.pdf

Life sciences study design

All studies must disclose on these points even when the disclosure is negative.

Sample size

Formal sample size calculation was not performed. Instead, sample sizes were determined based on data availability and resources.
We included available samples from participants who met our inclusion criteria; e.g. had both genotype information (GWAS) and a urine collection available. This approach ensures maximal use of existing resources.

From the Generation Scotland study (n=24,000) a subset of unrelated individuals with quality-controlled GWAS data and a spot urine sample stored at-80C° were included. Samples were randomly selected to the subset, but with an aim to balance the numbers of males and female participants (1,373 males, 1,370 females), resulting in 2,743 individuals.

From the VIKING study (n= 2,108) all samples with GWAS genotype data that passed genotyping quality control conditions and had a spot urine sample stored at -80C°, resulting in 2024 individuals. There were no other exclusions.

From the FinnDiane study (n>5,000) we included all participants that had GWAS data and a 24-hour urine collection (except for one overnight collection) stored at -20°C available. In addition, we only included individuals that met the meet the study-specific inclusion criteria regarding diabetes diagnosis and kidney function (see Data exclusions), resulting in 3,244 individuals.

Data exclusions

Generation Scotland: Participants without GWAS data and a spot urine sample were exlduded

VIKING study: Participants without GWAS data and a spot urine sample were exlduded.

FinnDiane: Participants without GWAS data and 24-hour urine collection were excluded. To ensure accurate diagnosis of Type 1 Diabetes (T1D), participants were required to have an age at onset of diabetes < 40 years and to have started insulin treatment within one year of diagnosis. Additionally, individuals with end-stage kidney disease (ESKD)—defined as those who had undergone kidney transplantation or dialysis—and those with an eGFR < 10 mL/min/1.73 m² on the day of urine collection were excluded.

Replication

We utilized external, publicly available summary data for replication. Specifically, we examined results from previously published urinary metabolite GWAS studies: Schlosser et al. (2023), which included 11 of the 16 metabolites tested in this study, and Raffler et al. (2015), which provided 2 additional metabolites missing from Schlosser et al. In total, we identified 24 out of 33 genetic variants for urinary metabolites present in either study. We found evidence of replication for 16 of the 24 signals in the replication data ($p < 0.0021$, adjusted for 24 variants, with concordant effect direction).

Randomization

The main study design was observational and therefore randomization is not relevant for this study

Blinding

The main study design was observational and therefore blinding is not relevant for this study

Behavioural & social sciences study design

All studies must disclose on these points even when the disclosure is negative.

Study description

Briefly describe the study type including whether data are quantitative, qualitative, or mixed-methods (e.g. qualitative cross-sectional, quantitative experimental, mixed-methods case study).

Research sample

State the research sample (e.g. Harvard university undergraduates, villagers in rural India) and provide relevant demographic information (e.g. age, sex) and indicate whether the sample is representative. Provide a rationale for the study sample chosen. For studies involving existing datasets, please describe the dataset and source.

Sampling strategy

Describe the sampling procedure (e.g. random, snowball, stratified, convenience). Describe the statistical methods that were used to predetermine sample size OR if no sample-size calculation was performed, describe how sample sizes were chosen and provide a rationale for why these sample sizes are sufficient. For qualitative data, please indicate whether data saturation was considered, and what criteria were used to decide that no further sampling was needed.

Data collection	*Provide details about the data collection procedure, including the instruments or devices used to record the data (e.g. pen and paper, computer, eye tracker, video or audio equipment) whether anyone was present besides the participant(s) and the researcher, and whether the researcher was blind to experimental condition and/or the study hypothesis during data collection.*
Timing	*Indicate the start and stop dates of data collection. If there is a gap between collection periods, state the dates for each sample cohort.*
Data exclusions	*If no data were excluded from the analyses, state so OR if data were excluded, provide the exact number of exclusions and the rationale behind them, indicating whether exclusion criteria were pre-established.*
Non-participation	*State how many participants dropped out/declined participation and the reason(s) given OR provide response rate OR state that no participants dropped out/declined participation.*
Randomization	*If participants were not allocated into experimental groups, state so OR describe how participants were allocated to groups, and if allocation was not random, describe how covariates were controlled.*

Ecological, evolutionary & environmental sciences study design

All studies must disclose on these points even when the disclosure is negative.

Study description	*Briefly describe the study. For quantitative data include treatment factors and interactions, design structure (e.g. factorial, nested, hierarchical), nature and number of experimental units and replicates.*
Research sample	*Describe the research sample (e.g. a group of tagged Passer domesticus, all Stenocereus thurberi within Organ Pipe Cactus National Monument), and provide a rationale for the sample choice. When relevant, describe the organism taxa, source, sex, age range and any manipulations. State what population the sample is meant to represent when applicable. For studies involving existing datasets, describe the data and its source.*
Sampling strategy	*Note the sampling procedure. Describe the statistical methods that were used to predetermine sample size OR if no sample-size calculation was performed, describe how sample sizes were chosen and provide a rationale for why these sample sizes are sufficient.*
Data collection	*Describe the data collection procedure, including who recorded the data and how.*
Timing and spatial scale	*Indicate the start and stop dates of data collection, noting the frequency and periodicity of sampling and providing a rationale for these choices. If there is a gap between collection periods, state the dates for each sample cohort. Specify the spatial scale from which the data are taken*
Data exclusions	*If no data were excluded from the analyses, state so OR if data were excluded, describe the exclusions and the rationale behind them, indicating whether exclusion criteria were pre-established.*
Reproducibility	*Describe the measures taken to verify the reproducibility of experimental findings. For each experiment, note whether any attempts to repeat the experiment failed OR state that all attempts to repeat the experiment were successful.*
Randomization	*Describe how samples/organisms/participants were allocated into groups. If allocation was not random, describe how covariates were controlled. If this is not relevant to your study, explain why.*
Blinding	*Describe the extent of blinding used during data acquisition and analysis. If blinding was not possible, describe why OR explain why blinding was not relevant to your study.*

Did the study involve field work? ☐ Yes ☐ No

Field work, collection and transport

Field conditions	*Describe the study conditions for field work, providing relevant parameters (e.g. temperature, rainfall).*
Location	*State the location of the sampling or experiment, providing relevant parameters (e.g. latitude and longitude, elevation, water depth).*
Access & import/export	*Describe the efforts you have made to access habitats and to collect and import/export your samples in a responsible manner and in compliance with local, national and international laws, noting any permits that were obtained (give the name of the issuing authority, the date of issue, and any identifying information).*
Disturbance	*Describe any disturbance caused by the study and how it was minimized.*

Reporting for specific materials, systems and methods

We require information from authors about some types of materials, experimental systems and methods used in many studies. Here, indicate whether each material, system or method listed is relevant to your study. If you are not sure if a list item applies to your research, read the appropriate section before selecting a response.

Materials & experimental systems

n/a	Involved in the study
☒	☐ Antibodies
☒	☐ Eukaryotic cell lines
☒	☐ Palaeontology and archaeology
☒	☐ Animals and other organisms
☒	☐ Clinical data
☒	☐ Dual use research of concern
☒	☐ Plants

Methods

n/a	Involved in the study
☒	☐ ChIP-seq
☒	☐ Flow cytometry
☒	☐ MRI-based neuroimaging

Antibodies

Antibodies used	*Describe all antibodies used in the study; as applicable, provide supplier name, catalog number, clone name, and lot number.*
Validation	*Describe the validation of each primary antibody for the species and application, noting any validation statements on the manufacturer's website, relevant citations, antibody profiles in online databases, or data provided in the manuscript.*

Eukaryotic cell lines

Policy information about cell lines and Sex and Gender in Research

Cell line source(s)	*State the source of each cell line used and the sex of all primary cell lines and cells derived from human participants or vertebrate models.*
Authentication	*Describe the authentication procedures for each cell line used OR declare that none of the cell lines used were authenticated.*
Mycoplasma contamination	*Confirm that all cell lines tested negative for mycoplasma contamination OR describe the results of the testing for mycoplasma contamination OR declare that the cell lines were not tested for mycoplasma contamination.*
Commonly misidentified lines (See ICLAC register)	*Name any commonly misidentified cell lines used in the study and provide a rationale for their use.*

Palaeontology and Archaeology

Specimen provenance	*Provide provenance information for specimens and describe permits that were obtained for the work (including the name of the issuing authority, the date of issue, and any identifying information). Permits should encompass collection and, where applicable, export.*
Specimen deposition	*Indicate where the specimens have been deposited to permit free access by other researchers.*
Dating methods	*If new dates are provided, describe how they were obtained (e.g. collection, storage, sample pretreatment and measurement), where they were obtained (i.e. lab name), the calibration program and the protocol for quality assurance OR state that no new dates are provided.*

☐ Tick this box to confirm that the raw and calibrated dates are available in the paper or in Supplementary Information.

Ethics oversight	*Identify the organization(s) that approved or provided guidance on the study protocol, OR state that no ethical approval or guidance was required and explain why not.*

Note that full information on the approval of the study protocol must also be provided in the manuscript.

Animals and other research organisms

Policy information about studies involving animals; ARRIVE guidelines recommended for reporting animal research, and Sex and Gender in Research

Laboratory animals	*For laboratory animals, report species, strain and age OR state that the study did not involve laboratory animals.*
Wild animals	*Provide details on animals observed in or captured in the field; report species and age where possible. Describe how animals were caught and transported and what happened to captive animals after the study (if killed, explain why and describe method; if released, say where and when) OR state that the study did not involve wild animals.*

Reporting on sex	*Indicate if findings apply to only one sex; describe whether sex was considered in study design, methods used for assigning sex. Provide data disaggregated for sex where this information has been collected in the source data as appropriate; provide overall numbers in this Reporting Summary. Please state if this information has not been collected. Report sex-based analyses where performed, justify reasons for lack of sex-based analysis.*
Field-collected samples	*For laboratory work with field-collected samples, describe all relevant parameters such as housing, maintenance, temperature, photoperiod and end-of-experiment protocol OR state that the study did not involve samples collected from the field.*
Ethics oversight	*Identify the organization(s) that approved or provided guidance on the study protocol, OR state that no ethical approval or guidance was required and explain why not.*

Note that full information on the approval of the study protocol must also be provided in the manuscript.

Clinical data

Policy information about clinical studies

All manuscripts should comply with the ICMJE guidelines for publication of clinical research and a completed CONSORT checklist must be included with all submissions.

Clinical trial registration	*Provide the trial registration number from ClinicalTrials.gov or an equivalent agency.*
Study protocol	*Note where the full trial protocol can be accessed OR if not available, explain why.*
Data collection	*Describe the settings and locales of data collection, noting the time periods of recruitment and data collection.*
Outcomes	*Describe how you pre-defined primary and secondary outcome measures and how you assessed these measures.*

Dual use research of concern

Policy information about dual use research of concern

Hazards

Could the accidental, deliberate or reckless misuse of agents or technologies generated in the work, or the application of information presented in the manuscript, pose a threat to:

No	Yes	
☒	☐	Public health
☐	☐	National security
☐	☐	Crops and/or livestock
☐	☐	Ecosystems
☐	☐	Any other significant area

Experiments of concern

Does the work involve any of these experiments of concern:

No	Yes	
☐	☐	Demonstrate how to render a vaccine ineffective
☐	☐	Confer resistance to therapeutically useful antibiotics or antiviral agents
☐	☐	Enhance the virulence of a pathogen or render a nonpathogen virulent
☐	☐	Increase transmissibility of a pathogen
☐	☐	Alter the host range of a pathogen
☐	☐	Enable evasion of diagnostic/detection modalities
☐	☐	Enable the weaponization of a biological agent or toxin
☐	☐	Any other potentially harmful combination of experiments and agents

Plants

Seed stocks	Not relevant to this study
Novel plant genotypes	Not relevant to this study
Authentication	Not relevant to this study

ChIP-seq

Data deposition

☐ Confirm that both raw and final processed data have been deposited in a public database such as GEO.

☐ Confirm that you have deposited or provided access to graph files (e.g. BED files) for the called peaks.

Data access links<br>*May remain private before publication.*	*For "Initial submission" or "Revised version" documents, provide reviewer access links. For your "Final submission" document, provide a link to the deposited data.*
Files in database submission	*Provide a list of all files available in the database submission.*
Genome browser session<br>(e.g. UCSC)	*Provide a link to an anonymized genome browser session for "Initial submission" and "Revised version" documents only, to enable peer review. Write "no longer applicable" for "Final submission" documents.*

Methodology

Replicates	*Describe the experimental replicates, specifying number, type and replicate agreement.*
Sequencing depth	*Describe the sequencing depth for each experiment, providing the total number of reads, uniquely mapped reads, length of reads and whether they were paired- or single-end.*
Antibodies	*Describe the antibodies used for the ChIP-seq experiments; as applicable, provide supplier name, catalog number, clone name, and lot number.*
Peak calling parameters	*Specify the command line program and parameters used for read mapping and peak calling, including the ChIP, control and index files used.*
Data quality	*Describe the methods used to ensure data quality in full detail, including how many peaks are at FDR 5% and above 5-fold enrichment.*
Software	*Describe the software used to collect and analyze the ChIP-seq data. For custom code that has been deposited into a community repository, provide accession details.*

Flow Cytometry

Plots

Confirm that:

☐ The axis labels state the marker and fluorochrome used (e.g. CD4-FITC).

☐ The axis scales are clearly visible. Include numbers along axes only for bottom left plot of group (a 'group' is an analysis of identical markers).

☐ All plots are contour plots with outliers or pseudocolor plots.

☐ A numerical value for number of cells or percentage (with statistics) is provided.

Methodology

Sample preparation	*Describe the sample preparation, detailing the biological source of the cells and any tissue processing steps used.*
Instrument	*Identify the instrument used for data collection, specifying make and model number.*
Software	*Describe the software used to collect and analyze the flow cytometry data. For custom code that has been deposited into a community repository, provide accession details.*

Cell population abundance | *Describe the abundance of the relevant cell populations within post-sort fractions, providing details on the purity of the samples and how it was determined.*

Gating strategy | *Describe the gating strategy used for all relevant experiments, specifying the preliminary FSC/SSC gates of the starting cell population, indicating where boundaries between "positive" and "negative" staining cell populations are defined.*

☐ Tick this box to confirm that a figure exemplifying the gating strategy is provided in the Supplementary Information.

Magnetic resonance imaging

Experimental design

Design type | *Indicate task or resting state; event-related or block design.*

Design specifications | *Specify the number of blocks, trials or experimental units per session and/or subject, and specify the length of each trial or block (if trials are blocked) and interval between trials.*

Behavioral performance measures | *State number and/or type of variables recorded (e.g. correct button press, response time) and what statistics were used to establish that the subjects were performing the task as expected (e.g. mean, range, and/or standard deviation across subjects).*

Acquisition

Imaging type(s) | *Specify: functional, structural, diffusion, perfusion.*

Field strength | *Specify in Tesla*

Sequence & imaging parameters | *Specify the pulse sequence type (gradient echo, spin echo, etc.), imaging type (EPI, spiral, etc.), field of view, matrix size, slice thickness, orientation and TE/TR/flip angle.*

Area of acquisition | *State whether a whole brain scan was used OR define the area of acquisition, describing how the region was determined.*

Diffusion MRI ☐ Used ☐ Not used

Preprocessing

Preprocessing software | *Provide detail on software version and revision number and on specific parameters (model/functions, brain extraction, segmentation, smoothing kernel size, etc.).*

Normalization | *If data were normalized/standardized, describe the approach(es): specify linear or non-linear and define image types used for transformation OR indicate that data were not normalized and explain rationale for lack of normalization.*

Normalization template | *Describe the template used for normalization/transformation, specifying subject space or group standardized space (e.g. original Talairach, MNI305, ICBM152) OR indicate that the data were not normalized.*

Noise and artifact removal | *Describe your procedure(s) for artifact and structured noise removal, specifying motion parameters, tissue signals and physiological signals (heart rate, respiration).*

Volume censoring | *Define your software and/or method and criteria for volume censoring, and state the extent of such censoring.*

Statistical modeling & inference

Model type and settings | *Specify type (mass univariate, multivariate, RSA, predictive, etc.) and describe essential details of the model at the first and second levels (e.g. fixed, random or mixed effects; drift or auto-correlation).*

Effect(s) tested | *Define precise effect in terms of the task or stimulus conditions instead of psychological concepts and indicate whether ANOVA or factorial designs were used.*

Specify type of analysis: ☐ Whole brain ☐ ROI-based ☐ Both

Statistic type for inference | *Specify voxel-wise or cluster-wise and report all relevant parameters for cluster-wise methods.*

(See Eklund et al. 2016)

Correction | *Describe the type of correction and how it is obtained for multiple comparisons (e.g. FWE, FDR, permutation or Monte Carlo).*

Models & analysis

n/a	Involved in the study
☐	☐ Functional and/or effective connectivity
☐	☐ Graph analysis
☐	☐ Multivariate modeling or predictive analysis

Functional and/or effective connectivity	*Report the measures of dependence used and the model details (e.g. Pearson correlation, partial correlation, mutual information).*
Graph analysis	*Report the dependent variable and connectivity measure, specifying weighted graph or binarized graph, subject- or group-level, and the global and/or node summaries used (e.g. clustering coefficient, efficiency, etc.).*
Multivariate modeling and predictive analysis	*Specify independent variables, features extraction and dimension reduction, model, training and evaluation metrics.*
